# Ubiquitin regulatory X (UBX) domain-containing protein 6 is essential for autophagy induction and inflammation control in macrophages

**DOI:** 10.1038/s41423-024-01222-1

**Published:** 2024-10-23

**Authors:** Young Jae Kim, Sung-Gwon Lee, So Young Park, Sang Min Jeon, Soo In Kim, Kyung Tae Kim, Taylor Roh, Sang-Hee Lee, Min Joung Lee, Jinyoung Lee, Hyeon Ji Kim, So Eui Lee, Jin Kyung Kim, Jun Young Heo, In Soo Kim, Chungoo Park, Seungwha Paik, Eun-Kyeong Jo

**Affiliations:** 1 Department of Microbiology, Chungnam National University College of Medicine, Daejeon, 35015 Republic of Korea; 2 Department of Medical Science, Chungnam National University College of Medicine, Daejeon, 35015 Republic of Korea; 3 Section of Genetics and Physiology, Laboratory of Molecular and Cellular Biology, National Institute of Diabetes and Digestive and Kidney Diseases (NIDDK), National Institutes of Health (NIH), Bethesda, MD 20892 USA; 4 School of Biological Sciences and Technology, Chonnam National University, Gwangju, 61186 Republic of Korea; 5 Division of Pulmonary, Allergy and Critical Care Medicine, Kangdong Sacred Heart Hospital, Hallym Medical Center, Seoul, 05355 Republic of Korea; 6 System Network Inflammation Control Research Center, Chungnam National University College of Medicine, Daejeon, 35015 Republic of Korea; 7 Center for Research Equipment, Korea Basic Science Institute, Cheongju, Chungbuk 28199 Republic of Korea; 8 Department of Biochemistry, Chungnam National University College of Medicine, Daejeon, 35015 Republic of Korea; 9 Department of Microbiology, Keimyung University School of Medicine, Daegu, 42601 Republic of Korea; 10 Department of Pharmacology, Chungnam National University College of Medicine, Daejeon, 35015 Republic of Korea

**Keywords:** UBXN6, Sepsis, Inflammation, Autophagy, Immunosuppression, Sepsis, Autophagy, Proteolysis, Cytokines, Innate immunity

## Abstract

Ubiquitin regulatory X (UBX) domain-containing protein 6 (UBXN6) is an essential cofactor for the activity of the valosin-containing protein p97, an adenosine triphosphatase associated with diverse cellular activities. Nonetheless, its role in cells of the innate immune system remains largely unexplored. In this study, we report that UBXN6 is upregulated in humans with sepsis and may serve as a pivotal regulator of inflammatory responses via the activation of autophagy. Notably, the upregulation of UBXN6 in sepsis patients was negatively correlated with inflammatory gene profiles but positively correlated with the expression of Forkhead box O3, an autophagy-driving transcription factor. Compared with those of control mice, the macrophages of mice subjected to myeloid cell-specific UBXN6 depletion exhibited exacerbated inflammation, increased mitochondrial oxidative stress, and greater impairment of autophagy and endoplasmic reticulum-associated degradation pathways. UBXN6-deficient macrophages also exhibited immunometabolic remodeling, characterized by a shift to aerobic glycolysis and elevated levels of branched-chain amino acids. These metabolic shifts amplify mammalian target of rapamycin pathway signaling, in turn reducing the nuclear translocation of the transcription factor EB and impairing lysosomal biogenesis. Together, these data reveal that UBXN6 serves as an activator of autophagy and regulates inflammation to maintain immune system suppression during human sepsis.

## Introduction

The innate immune system serves as a primary defense mechanism that detects and responds to pathogen- or damage-associated molecular patterns during infection and inflammation. Monocytes and macrophages, as principal cells in the innate immune system, can elicit detrimental inflammatory responses when their function becomes aberrant [1–3]. A delicate balance between inflammation and innate immunity is crucial for effectively orchestrating protective immune responses against various pathogenic invasions [2, 3]. Understanding the mechanisms by which monocytes/macrophages regulate inflammation and innate immunity is crucial for the development of novel therapeutics to combat infections and manage inflammatory diseases such as sepsis, a life-threatening systemic inflammatory condition. Despite the importance of this knowledge, the mechanisms underlying the delicate balance between inflammation and innate immunity remain incompletely understood.

Proteostasis is a dynamic and vital biological process for maintaining protein homeostasis and involves several pathways, such as autophagy, endoplasmic reticulum-associated protein degradation (ERAD), and proteasomal degradation [4]. Autophagy, a lysosomal catabolic pathway that degrades large protein aggregates and damaged organelles, plays a crucial role in the regulation of immune and inflammatory responses, thereby partially controlling the pathological processes associated with inflammation [5, 6]. The ERAD pathway is highly conserved in evolutionary terms and degrades misfolded or unfolded proteins of mammalian cells to maintain protein homeostasis [7, 8]. Valosin-containing protein (VCP)/p97 is a significant member of the ATPases associated with diverse cellular activities (AAA+) superfamily that facilitates the conversion of chemicals into mechanical energy in many organisms [9–11]. The p97 is an abundant cytosolic protein in mammalian cells that regulates autophagy, ERAD, gene expression, and organelle biogenesis, thereby maintaining cellular homeostasis [10, 12, 13].

Several cofactors ( > 40), including “ubiquitin regulatory X” (UBX) domain-containing proteins, interact with p97 and participate in its functional regulation by facilitating substrate recruitment, leading to the formation of various p97-cofactor complexes [10, 13]. UBXN6 is a novel p97 cofactor that associates with the p97 complex via both peptide:N-glycanase and ubiquitin-associated or UBX-containing proteins (PUB) [14, 15] and UBX domains [16]. A recent study revealed that UBXN6 contains p97-remodeling motifs that drive AAA+ remodeling and ring opening, thereby regulating p97 ATPase activity [17]. Both p97 and UBXN6 preserve lysosomal homeostasis by facilitating the clearance of damaged lysosomes and activating lysophagy [18–20]. In addition, both UBXN6 and p97 control the trafficking of ubiquitylated caveolin-1 within the endocytic pathway [21]. Moreover, p97 activity is essential for the production of the autophagy-inducing lipid phosphatidylinositol-3-phosphate and for autophagosome biogenesis [22]. A recent study demonstrated that p97, UBXN6, and ANKRD13A act cooperatively to target the parasitophorous vacuole, thereby restricting *Toxoplasma gondii* infection in interferon-stimulated human endothelial cells [23]. Nonetheless, the specific involvement of the p97 cofactor UBXN6 in the regulation of innate immune and inflammatory responses and its clinical relevance remain largely unexplored.

Here, we revealed significantly upregulated levels of UBXN6 in the peripheral blood mononuclear cells (PBMCs) of septic patients. Single-cell RNA sequencing (scRNA-seq) analysis revealed that UBXN6 was predominantly expressed in human primary monocytes/macrophages. In sepsis patients, UBXN6 levels were negatively correlated with inflammatory gene profiles but positively correlated with the levels of Forkhead box O3 (*FOXO3*) and several autophagy/mitophagy-related genes. Next, we established myeloid-specific UBXN6-deficient mice, which exhibited increased susceptibility to systemic inflammation. Mechanistically, myeloid UBXN6 plays a pivotal role in inducing both autophagy and ERAD by regulating mitochondrial and cellular oxidative stress, such as reactive oxygen species (ROS), thus influencing inflammatory responses in macrophages. Furthermore, myeloid UBXN6 deficiency shifted immunometabolic remodeling toward aerobic glycolysis and increased the levels of branched-chain amino acids (BCAAs), thus increasing mammalian target of rapamycin (mTOR) pathway activation and limiting both the nuclear translocation of transcription factor EB (TFEB) and lysosomal biogenesis. Thus, myeloid UBXN6 is an essential activator of autophagy and controller of inflammation. Our data also highlight the clinical relevance of UBXN6 in terms of human sepsis, particularly in the context of immunosuppression.

## Results

### Upregulation of UBXN6 in sepsis patients

**Fig. 1:**
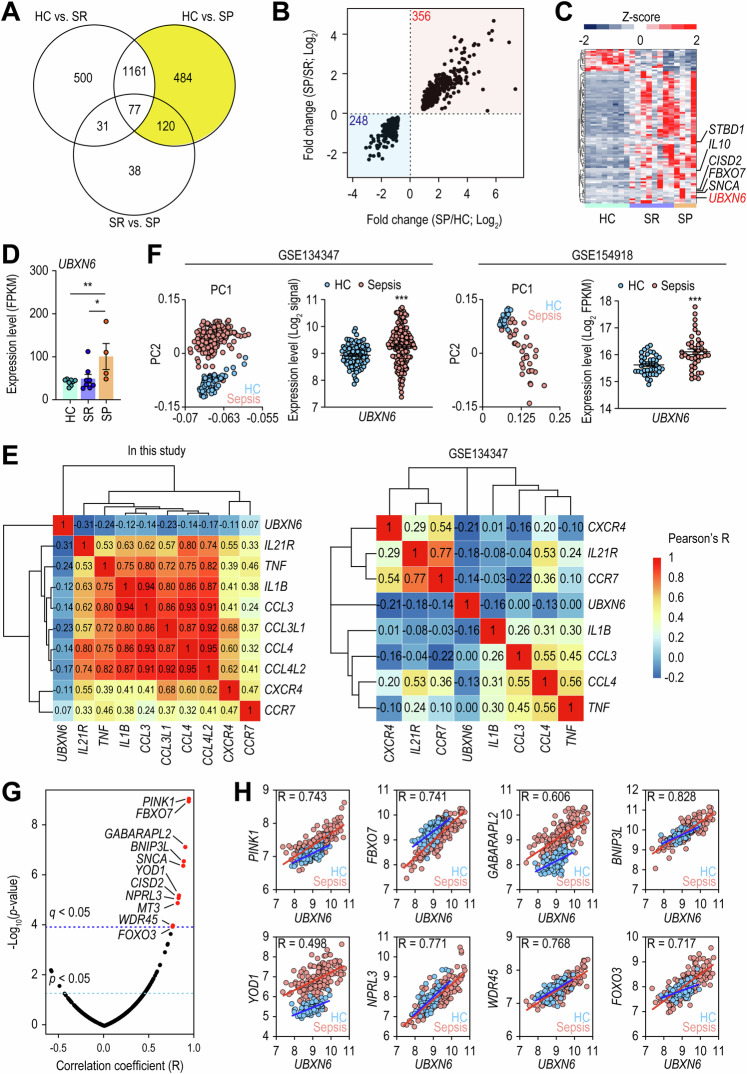
Compared with healthy controls, sepsis patients presented upregulated expression levels of UBXN6. **A** Diagram illustrating the number of DEGs identified by comparing HCs to SRs, HCs to SPs, and SRs to SPs. The yellow region highlights DEGs whose expression varies between HCs and SPs but not between HCs and SRs. **B** Graph depicting the fold changes in 604 genes from the yellow area of (**A**), with red indicating upregulated DEGs and blue indicating downregulated DEGs in SP. **C** Heatmap representing 91 ATG genes with differential expression. Hierarchical clustering of DEGs was performed on the basis of the Euclidean distance of relative expression. **D** Expression of *UBXN6* in human PBMCs from HCs, SRs, and SPs in our cohort. *q value < 0.05, **q value < 0.01. **E** Gene expression correlations between *UBXN6* and several inflammatory genes identified in our cohort and the publicly available GSE134347 dataset. The correlation coefficients are clustered hierarchically via the Euclidean distance method. **F** Principal component analysis of data from two publicly available cohorts, GSE134347 and GSE154918. The expression patterns of *UBXN6* between HCs and sepsis patients within these cohorts are depicted. For GSE134347, microarray data were used, with log_2_-transformed signal intensities employed as expression levels. For GSE154918, RNA-seq data were used, with log_2_-transformed FPKM values representing expression levels. **G** Correlation test results for *UBXN6* and 499 ATG genes. The 11 genes marked with red dots have a positive correlation with a statistically significant adjusted *p* value (*q* value) with a Bonferroni correction of less than 0.05. **H** Expression levels (log_2_-signal) and correlation values of *UBXN6* and the 8 ATG genes in the GSE134347 cohort. The blue and red lines represent linear regressions for HCs and sepsis patients, respectively. The Pearson correlation coefficient (R) between the *UBXN6* expression level and each gene was estimated via the correlation function implemented in R. All correlation tests yielded *p* values less than 2.2 × 10^−^^16^. HC, healthy controls; SP, patients with a poor prognosis; SR, patients who had recovered; FPKM, fragments per kilobase of transcript per million mapped reads. *p < 0.05, **p < 0.01, and ***p < 0.001

To examine the overall changes in gene expression in sepsis patients, we used deep RNA-seq to perform transcriptome analyses of PBMCs from patients with a poor prognosis (SP), patients who had recovered (SR), and healthy controls (HC). In total, 1.44 billion raw reads were generated from 8 HCs and 12 sepsis patients (8 SRs and 4 SPs) and trimmed to remove adapter and low-quality sequences. On average, 68.1 million clean reads were produced per sample and mapped to the reference human genome (Supplementary Table 1). From the resulting alignments, we identified differentially expressed genes (DEGs) between the SP, SR, and HC groups by performing comparative transcriptome analysis. After controlling for multiple comparisons and a false discovery rate (FDR) of 5%, we acquired 1,769, 1,840, and 266 DEGs via comparisons between the SR and HC, SP and HC, and SP and SR data (Fig. 1A). To identify potential PBMC biomarkers of SP, we investigated 604 (484 + 120) DEGs shared by HCs and SPs while excluding those shared by SRs and HCs. Among these DEGs, 356 and 248 DEGs were commonly upregulated or downregulated, respectively, in SP patients (Fig. 1B). The downregulated DEGs were significantly enriched in immune system-related biological processes, including the cell surface receptor signaling pathway (GO:0007166), positive regulation of interferon-γ production (GO:0032729), T-cell activation (GO:0008009), Th17 cell differentiation (hsa04659), and cytokine–cytokine receptor interaction (hsa04060) (Supplementary Fig. 1A, B), suggesting that SP patients exhibited immunosuppressive profiles.

Immunosuppression in sepsis is a complex and critical aspect of the pathophysiology of sepsis. Emerging research has shed light on the intricate relationship between immunosuppression and autophagy [24, 25]. Thus, we examined the expression profiles of 499 autophagy-related (ATG) genes obtained from GO resources (https://geneontology.org) and discovered 91 DEGs in sepsis patients (SR and SP) compared with HCs (Fig. 1C). In these patients, the expression of 85.7% (78 of 91) of the ATG DEGs was increased, indicating the activation of autophagy. Among these DEGs, *UBXN6/UBXD1*, *FBXO7*, *CISD2*, *STBD1*, *SNCA*, and *IL10* exhibited significant differences between SR and SP patients (Fig. 1C, D). We specifically focused on *UBXN6* given its association with the regulation of signaling pathways related to inflammatory cytokines, such as the Janus kinase-signal transducer and activator of transcription and nuclear factor (NF)-κB pathways [26, 27]. In line with previous findings, *UBXN6* expression was negatively correlated with the expression of DEGs associated with inflammatory genes such as cytokines/chemokines and their receptors, including *IL21R*, *TNF*, *IL1B*, *CCL3*, *CCL4*, *CXCR4*, and *CCR7* (Fig. 1E). An analysis of data from publicly available sepsis cohorts [28, 29] further revealed that, compared with HCs, sepsis patients in both cohorts presented significantly increased expression of *UBXN6*, which was inversely correlated with the expression levels of inflammatory genes (Fig. 1F).

Next, we investigated the relationships between UBXN6 and ATG genes (Fig. 1G). We found that 11 ATG genes were strongly correlated with *UBXN6* expression. Similarly, analysis of the GSE134347 cohort revealed a robust positive correlation between *UBXN6* expression and *FOXO3* expression and between UBXN6 expression and the expression of several ATG genes (*PINK1*, *FBXO7*, *GABARAPL2*, *BNIP3L*, *YOD1*, *NPRL3*, and *WDR45*) (Fig. 1H). These findings suggest that *UBXN6* is upregulated in human sepsis patients and that its level is negatively correlated with inflammatory gene expression but positively correlated with the expression of *FOXO3* and several ATG genes.

### Myeloid UBXN6 is required for the regulation of inflammatory responses triggered by innate immune stimuli

**Fig. 2:**
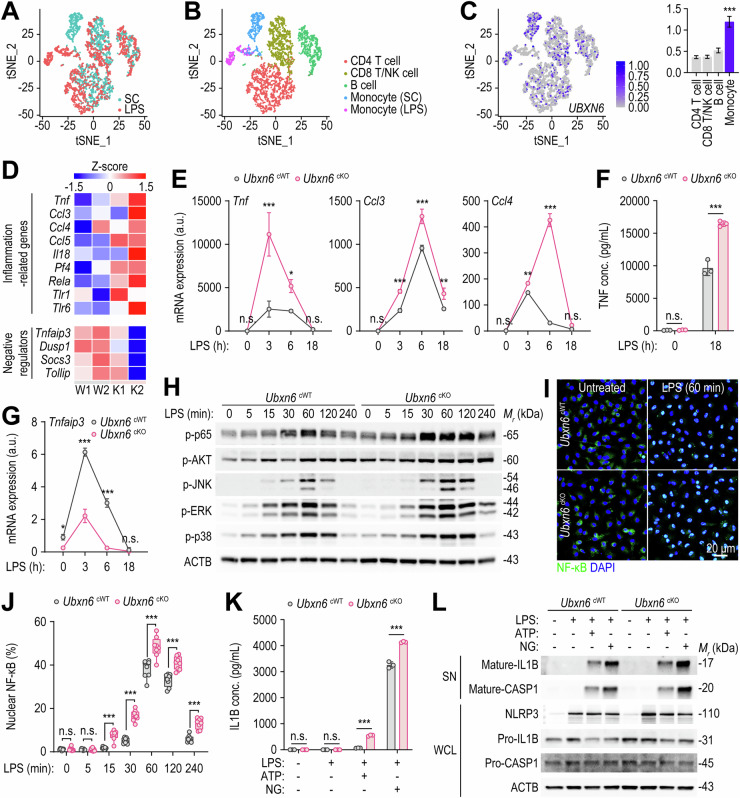
The myeloid expression of UBXN6 controls LPS-induced proinflammatory responses in vitro. **A** Scatter plot based on tSNE analysis, depicting cell populations from both the solvent control (SC) and the LPS-stimulated (LPS) groups of human PBMCs. **B** Five distinct clusters identified and annotated via known cell type markers (refer to Fig. S2). **C** The section visualizing the expression of *UBXN6*, with a bar graph on the right displaying normalized *UBXN6* read counts for each cluster. Statistical significance was determined via Wilcoxon’s rank sum test to compare the normalized read counts of *UBXN6* between the monocyte clusters (SC and LPS) and each CD4, CD8/NK, and B-cell cluster. The *p* values for each comparison were all less than 1 × 10^−^^10^. **D** Heatmap of representative inflammation-associated genes and negative regulatory genes related to inflammatory responses expressed in BMDMs after LPS stimulation (100 ng/mL) for 3 h. **E** Relative expression levels of *Tnf*, *Ccl3*, and *Ccl4* mRNAs in BMDMs. The cells were stimulated with LPS (100 ng/mL) for the indicated periods and then lysed for qRT‒PCR. **F** TNF protein levels in the culture supernatants of BMDMs primed with or without LPS (100 ng/mL) were analyzed via ELISA. **G** Relative mRNA expression levels of *Tnfaip3* in BMDMs stimulated with LPS (100 ng/mL) at the indicated times. **H** Western blotting of phosphorylated p65, AKT, JNK, ERK, and p38 protein levels in BMDMs treated with LPS (100 ng/mL) for the indicated times; ACTB served as the loading control. **I**, **J** Representative images (**I**) and quantification (**J**) of NF-κB nuclear translocation in BMDMs stimulated with LPS (100 ng/mL) for the indicated periods. The cells were stained with anti-NF-κB antibodies (green) for NF-kB and with DAPI (blue) for nucleic acid detection. Confocal microscopy was used to determine the fluorescence intensity of interest, which was then analyzed with FIJI software. **K**, **L** IL-1B protein levels in BMDMs were measured via ELISA (**K**) and Western blotting (**L**). BMDMs were primed with or without LPS (100 ng/mL) for 4 h, followed by stimulation with or without ATP (5 mM) or nigericin (10 μM) for 45 min. NLRP3 and the mature and precursor forms of IL1B and CASP1 were examined. ACTB represents the loading control (**L**). One-way ANOVA with Tukey’s multiple comparison test (**E**, **G**, and **K**), two-tailed Student’s *t* test (**F**), or two-way ANOVA with Sidak’s multiple comparison test (**J**) was used to determine statistical significance. SC, solvent control; LPS, lipopolysaccharide; tSNE, t-distributed stochastic neighbor embedding; n.s., not significant; a.u., arbitrary unit; ATP, adenosine triphosphate; NG, nigericin; SN, supernatant; WCL, whole-cell lysate. The data are presented as the means ± SD from at least three independent experiments (**E**–**G**, **J**, and **K**). *p < 0.05, **p < 0.01, and ***p < 0.001

To gain insight into the principal cell types expressing UBXN6, we conducted scRNA-seq experiments on PBMCs from HCs treated with lipopolysaccharide (LPS) or a solvent control (SC). Following data preprocessing and quality control, we successfully obtained single-cell transcriptomes from 3022 SC-treated cells and 2098 LPS-treated cells (Fig. 2A). Further analysis revealed five distinct clusters of CD4 T cells, CD8 and NK cells, B cells, control monocytes, and LPS-treated monocytes in the transcriptome profiles. The monocyte population expressing high levels of monocyte/macrophage markers such as CD14, FCGR3A, LYZ, and MS4A7 increased after LPS treatment (Fig. 2B and Supplementary Fig. 2). Notably, UBXN6 was expressed primarily in monocytes rather than in T or B cells (Fig. 2C). As UBXN6 expression is increased in monocyte/macrophage types, we generated myeloid-specific UBXN6-deficient (cKO) mice by crossing *Ubxn6*^flox/flox^ mice with *Lyz2*^Cre^ mice. Polymerase chain reaction (PCR) and Western blotting analysis revealed that the UBXN6 gene and protein expression levels in bone marrow-derived macrophages (BMDMs) from cKO mice were significantly lower than those in BMDMs from *Ubxn6*^flox/flox^ (cWT) mice (Supplementary Fig. 3A, B).

To investigate the role of UBXN6 in the inflammatory pathways of monocytes/macrophages, bulk RNA-seq analysis was performed on BMDMs from cWT and cKO mice exposed to LPS, a Toll-like receptor (TLR) 4 ligand. Heatmap analysis revealed that the relative expression levels of several proinflammatory cytokines, including *Tnf* and *Il18*, and chemokines, including *Ccl3*, *Ccl4*, and *Ccl5*, were greater in cKO BMDMs than in cWT BMDMs (Fig. 2D). Validation analysis via quantitative real‑time PCR (qRT‑PCR) revealed that the *Tnf*, *Ccl3*, and *Ccl4* expression levels were significantly increased in cKO BMDMs in response to both LPS and zymosan (a dectin-1 and TLR2 agonist) stimulation (Fig. 2E and Supplementary Fig. 4). TNF secretion was also significantly greater in the cKO BMDMs than in the cWT BMDMs after 18 h of LPS treatment (Fig. 2F). We further examined the immunomodulatory function of UBXN6 in human primary monocytes by transducing cells with short hairpin RNA (shRNA) specifically targeting *UBXN6* (sh*UBXN6*). Consistent with the findings in murine cKO BMDMs, knocking down *UBXN6* in human primary monocytes significantly increased the mRNA expression levels of *TNF*, *CCL3*, and *CCL4* (Supplementary Fig. 5A, B) as well as TNF secretion (Supplementary Fig. 5C) in response to LPS stimulation.

In addition, specific reductions in the levels of *Tnfaip3*/*A20*, *Dusp1*, *Socs3*, and *Tollip*, which are negative regulators of innate immune system signaling, were noted in cKO BMDMs (Fig. 2D). Interestingly, *Tnfaip3* was markedly downregulated in cKO BMDMs compared with that in cWT BMDMs, even without LPS treatment (Fig. 2G). Next, we focused on the inflammatory signaling pathways that orchestrate the innate immune response. As shown in Fig. 2H, compared with cWT BMDMs, cKO BMDMs presented significant upregulation of the NF-κB pathway and the levels of AKT and mitogen-activated protein kinases (MAPKs), such as Jun-amino-terminal kinase (JNK), extracellular-signal-regulated kinase (ERK), and p38. Consistent with these findings, NF-κB immunostaining revealed a significant increase in nuclear translocation, which was greater in cKO BMDMs than in cWT BMDMs from 15–240 min after the initiation of LPS treatment (Fig. 2I, J). Together, these data suggest that inflammatory cytokine/chemokine generation and the relevant signaling pathways are significantly upregulated in UBXN6-deficient BMDMs compared with those in cWT BMDMs in response to various stimuli of the innate immune system.

Next, we explored whether UBXN6 deficiency modulated the activation of the NLRP3 inflammasome, which is critical for the innate defense of patients with autoinflammatory and autoimmune diseases [30]. Compared with cWT BMDMs, cKO BMDMs secreted higher levels of IL-1B after exposure to LPS followed by ATP or nigericin treatment (Fig. 2K). Western blotting further revealed increased levels of mature IL1B and CASP1 in cKO BMDMs compared with those in cWT BMDMs in response to stimulation with the NLRP3 inflammasome (Fig. 2L). Notably, the NLRP3 protein level was significantly increased in the LPS-primed cKO BMDMs (Fig. 2L). We further investigated whether UBXN6 deficiency results in the upregulation of CASP11, a key factor in the detection of intracellular bacteria or cytosolic LPS, leading to noncanonical activation of the NLRP3 inflammasome [31, 32]. Our findings revealed that myeloid-specific UBXN6 deficiency significantly increased CASP11 expression in response to cytosolic LPS (Supplementary Fig. 6A, B). These results collectively suggest that UBXN6 plays a crucial role in regulating the canonical and noncanonical activation of the NLRP3 inflammasome in innate immune cells. In summary, UBXN6 is expressed predominantly in monocytes/macrophages and is essential for the regulation of inflammatory responses triggered by various innate immune signaling pathways.

### Myeloid UBXN6 is critical for inducing autophagy in response to diverse autophagic stimuli

**Fig. 3:**
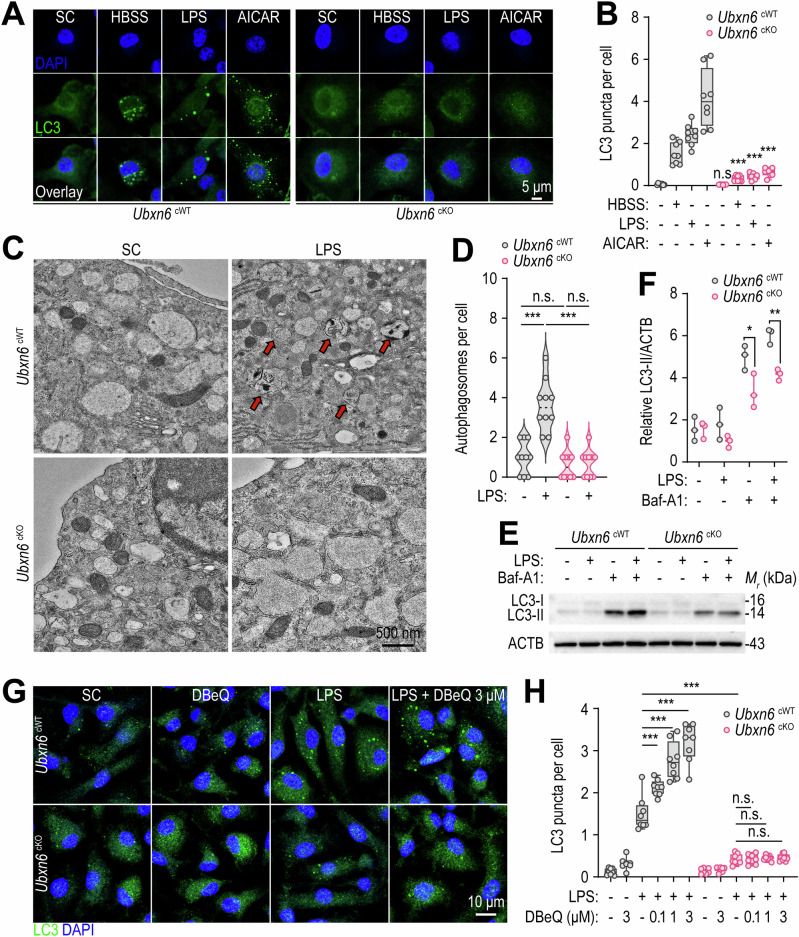
UBXN6 is required for the induction of autophagy in murine BMDMs. **A**, **B** Representative immunostaining images (**A**) and quantification (**B**) of LC3 puncta with anti-LC3 antibodies (green) for LC3 and DAPI (blue) for nucleic acid detection. BMDMs were starved with HBSS for 12 h, stimulated with LPS (100 ng/mL) for 18 h, or treated with or without AICAR (0.5 mM) for 24 h. **C** TEM images of BMDMs stimulated with or without LPS (100 ng/mL) for 18 h. The red arrows indicate autophagosomes. **D** The number of autophagosomes per cell quantified in the samples from (**C**). **E** LC3 conversion rates in BMDMs determined by Western blotting. The cells were treated with or without LPS (100 ng/mL) for 18 h in the presence or absence of Baf-A1 (100 nM). **F** Relative LC3-II levels normalized against ACTB in (**E**). **G**, **H** Immunofluorescence images (**G**) and quantification (**H**) of LC3 puncta formation in BMDMs via confocal microscopy. The macrophages were pretreated with or without DBeQ (0.1, 1, or 3 μM) for 1 h, followed by stimulation with or without LPS (100 ng/mL) for 18 h. Statistical significance was determined via two-tailed Student’s *t* test (**B** and **F**) or one-way ANOVA with Tukey’s multiple comparison test (**D** and **H**). SC, solvent control; LPS, lipopolysaccharide; AICAR, 5-aminoimidazole-4-carboxamide ribonucleoside; n.s., not significant; Baf-A1, bafilomycin A1. The data are presented as the means ± SD from at least three independent experiments (**B**, **D**, **F**, and **H**). *p < 0.05, **p < 0.01, and ***p < 0.001

Next, on the basis of observations in humans, we investigated the role of UBXN6 in autophagy, which protects against the effects of both exogenous and endogenous noxious stimuli [33]. We investigated whether UBXN6 affects autophagy in macrophages exposed to starvation conditions or stimulants such as LPS and 5-aminoimidazole-4-carboxamide ribonucleoside (AICAR). We found that cKO BMDMs and sh*UBXN6*-transduced human primary monocytes presented significant loss of LC3 puncta when exposed to various autophagic stimuli (Fig. 3A, B, for cKO BMDMs; Supplementary Fig. 7A‒C, for human primary monocytes). In addition, the number of autophagosomes observed via transmission electron microscopy (TEM) was drastically lower in cKO BMDMs than in cWT BMDMs after LPS stimulation (Fig. 3C, D). When treated with bafilomycin A1 (Baf-A1), an inhibitor of autophagic flux [34], cKO BMDMs presented a significantly reduced LC3-II/ACTB ratio, suggesting that autophagy induction was hampered (Fig. 3E, F).

In addition, the small molecule inhibitor of p97 N2,N4-dibenzylquinazoline-2,4-diamine (DBeQ), which reduces autophagosome maturation [35], induced significant accumulation of LC3 autophagosomes in cWT BMDMs in the presence of LPS (Fig. 3G, H). However, the number of LC3 puncta in the cKO BMDMs was significantly lower than that in the cWT BMDMs, which was largely unaffected by DBeQ treatment (Fig. 3G, H). Furthermore, the small-molecule enhancer of rapamycin 28 (SMER28) [36], which binds to p97 and enhances its activity [37], did not rescue autophagosome formation in UBXN6-deficient macrophages (Supplementary Fig. 8A, B). These data strongly suggest an essential role for UBXN6 in terms of autophagy induction, irrespective of its ability to modulate p97 activity.

We next investigated whether UBXN6 independently induces autophagy and inhibits TLR signaling or if the two pathways are interconnected. To examine this, we assessed the effects of Baf-A1 treatment on LPS-induced inflammatory responses in *Ubxn6* cWT and cKO BMDMs. As shown in Supplementary Fig. 9, Baf-A1 treatment increased LPS-induced inflammatory cytokine production in cWT BMDMs, whereas the same treatment failed to enhance the inflammatory response in cKO BMDMs. These data strongly suggest that UBXN6-induced autophagy negatively regulates LPS-induced inflammation.

### The UBXN6-FOXO3 axis is required for inducing autophagy and mitophagy in macrophages

*FOXO3*, an ATG gene whose expression correlates with that of *UBXN6* (Fig. 1G, H), is a major transcription factor that directly regulates the autophagic process [38–40]. Thus, we investigated the mechanistic role of the UBXN6-FOXO3 axis in terms of activating autophagy. Initially, we measured the expression levels of *Foxo3* and its downstream genes, including *Gabarapl2*, *Pink1*, and *Bnip3l*, in cWT and cKO BMDMs in response to LPS stimulation or starvation. Notably, the levels of mRNAs encoding *Foxo3*, *Gabarapl2*, *Pink1*, and *Bnip3l* were significantly decreased by LPS stimulation of cKO BMDMs (Supplementary Fig. 10A). The mRNA expression level of *Gabarapl2*, *but not Pink1* or *Bnip3l*, was significantly downregulated in cKO BMDMs subjected to starvation (Supplementary Fig. 10B). These data suggest that the UBXN6‒FOXO3 axis is required for the expression of FOXO3 downstream genes in response to autophagic stimuli.

Next, we studied the impact of FOXO3 on autophagy activation in cWT and cKO BMDMs following LPS stimulation. *Foxo3* knockdown significantly reduced LC3 puncta formation in cWT BMDMs following LPS stimulation, whereas UBXN6 deficiency markedly compromised LC3-positive autophagosome formation in cKO BMDMs, regardless of *Foxo3* knockdown status (Supplementary Fig. 10C, D). Moreover, the levels of both *Pink1* and *Bnip3l*, which are critical factors in mitophagy activation [41, 42], were significantly reduced in cKO BMDMs following LPS stimulation (Supplementary Fig. 10A), suggesting impaired regulation of mitophagy in UBXN6-deficient cells. To further investigate this, we assessed the colocalization levels of LC3 and MitoTracker Red, a mitochondrial marker, and found that colocalization was significantly reduced in cKO BMDMs (Supplementary Fig. 11A, B). Furthermore, the accumulation of cytosolic mitochondrial DNA (mtDNA), indicative of defective mitophagy [43], was significantly elevated in cKO BMDMs post-LPS stimulation (Supplementary Fig. 11C). These findings suggest that UBXN6-mediated FOXO3 activation is crucial for autophagy and mitophagy induction.

### Myeloid UBXN6 plays an important role in activating ERAD and controlling mtROS-induced inflammation

**Fig. 4:**
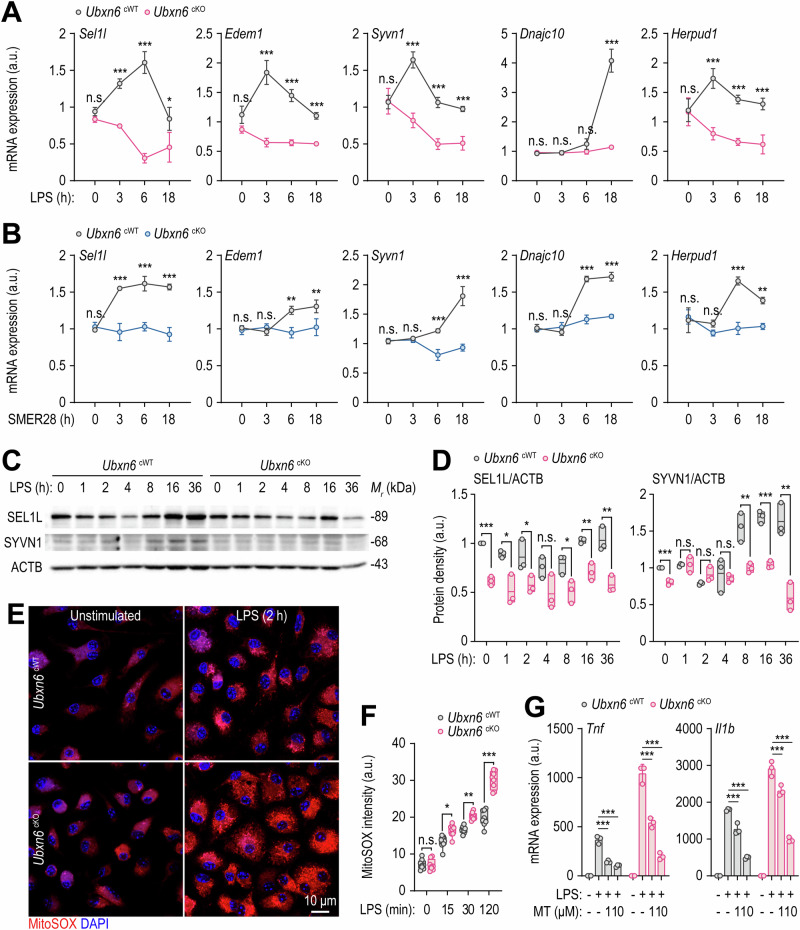
Myeloid UBXN6 is essential for enhancing ERAD and inhibiting inflammation induced by damaged mitochondria in LPS-primed macrophages. **A**, **B** Expression levels of *Sel1l*, *Edem1*, *Syvn1*, *Dnajc10*, and *Herpud1* mRNAs in BMDMs measured by qRT‒PCR after treatment with LPS (100 ng/mL) (**A**) or SMER28 (20 μM) (**B**) for the indicated times. **C** Representative immunoblots showing the time course of SEL1L and SYVN1 protein induction in BMDMs. ACTB represents the loading control. **D** Relative quantitative analysis of SEL1 or SYVN1 levels normalized to ACTB in (**C**). **E**, **F** Representative images (**E**) and quantification (**F**) of mtROS levels in BMDMs stained with MitoSOX (1 μM) for 20 min after stimulation with LPS (100 ng/mL) for the indicated times. **G** The expression levels of *Tnf* and *Il1b* mRNAs in BMDMs after 1 h of pretreatment with or without MitoTEMPO (1 or 10 μM). The BMDMs were then stimulated with or without LPS (100 ng/mL) for 6 h. Statistical significance was determined via one-way ANOVA with Tukey’s multiple comparison test (**A**, **B**, **F**, and **G**) or two-tailed Student’s *t* test (**D**). SC, solvent control; LPS, lipopolysaccharide; n.s., not significant; a.u., arbitrary unit; MT, MitoTEMPO. The data represent the means ± SD (**A**, **B**, **D**, **F**, and **G**) from at least three independent experiments. *p < 0.05, **p < 0.01, and ***p < 0.001

The defective induction of autophagy in cKO BMDMs prompted us to investigate its involvement in the regulation of the ERAD machinery, another cellular process that is essential for proteostasis [44]. To investigate whether myeloid UBXN6 regulates the ERAD pathway, the expression levels of key genes associated with ERAD, including *Sel1l*, *Edem1*, *Syvn1/Hrd1*, *Dnajc10*, and *Herpud1*, were analyzed in both cWT and cKO BMDMs in response to both LPS and SMER28 via qRT‒PCR. Compared with those in cWT BMDMs, the expression levels of all genes in cKO BMDMs were significantly lower in response to LPS (Fig. 4A) and SMER28 (Fig. 4B). Additionally, Western blotting revealed that SEL1L and SYVN1 protein levels were significantly reduced in the *Ubxn6* cKO BMDMs before and after LPS stimulation (Fig. 4C, D). Furthermore, we investigated whether UBXN6 is involved in the transcriptional activation of ERAD gene expression in response to starvation. As shown in Supplementary Fig. 12, the expression levels of genes involved in ERAD were significantly downregulated in cKO BMDMs in response to starvation. These data indicate that UBXN6 plays an important role in activating ERAD in response to innate immune stimuli or starvation.

Defective autophagy and ERAD are associated with mitochondrial dysfunction, leading to the accumulation of mtROS [45, 46]. Thus, we further investigated mtROS levels in both cWT and cKO BMDMs. The absence of UBXN6 significantly increased LPS-induced mtROS generation in cKO BMDMs compared with that in cWT cells, even after only 15 min of LPS treatment (Fig. 4E, F). To explore whether elevated mtROS levels contribute to increased inflammatory responses in cKO BMDMs, we treated both cWT and cKO BMDMs with MitoTEMPO, a mtROS inhibitor. Pretreatment with MitoTEMPO markedly reduced the expression levels of inflammatory cytokines and chemokines, such as *Tnf*, *Il1b*, *Ccl3*, and *Ccl4*, in both cWT and cKO BMDMs in a dose-dependent manner (Fig. 4G and Supplementary Fig. 13).

The ER plays a critical role in maintaining proteostasis, and ER stress is triggered when misfolded and unfolded proteins accumulate in the ER [47]. We investigated whether the gene expression levels of ER stress sensors differ between cWT and cKO BMDMs. Notably, the ER stress-related gene expression levels were significantly greater in the cKO BMDMs than in the cWT BMDMs (Supplementary Fig. 14A). In addition to the mitochondria, the ER also significantly contributes to the generation of ROS, primarily as a byproduct of protein folding [48]. We next examined how myeloid UBXN6 impacts cellular ROS levels and found that ROS levels were significantly elevated in cKO BMDMs both before and after LPS stimulation (Supplementary Fig. 14B). These findings highlight the specific role of UBXN6 in regulating ERAD and ER stress while controlling ROS levels, thereby modulating excessive inflammatory responses in macrophages.

### UBXN6 deficiency induces immunometabolic remodeling toward aerobic glycolysis and alters amino acid levels

**Fig. 5:**
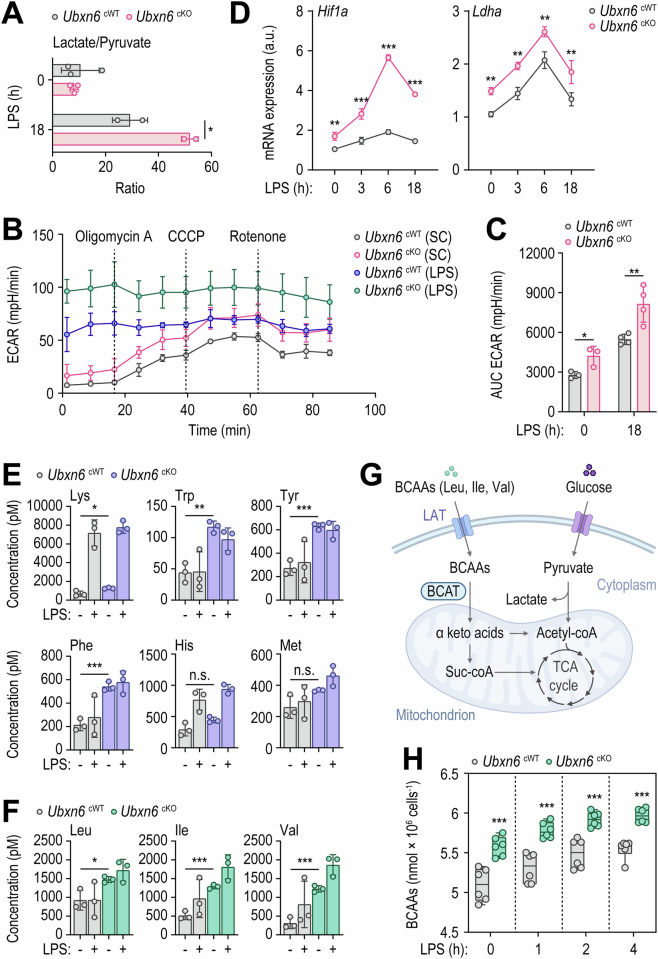
Compared with WT macrophages, UBXN6-deficient macrophages presented elevated levels of essential amino acids and increased aerobic glycolysis. **A**–**C** Lactate/pyruvate ratio (**A**), ECAR data (**B**), and AUC of the ECAR (**C**) evaluated in BMDMs stimulated with or without LPS (100 ng/mL) for 18 h. **D** Relative mRNA expression levels of *Hif1a* and *Ldha* in BMDMs after treatment with LPS (100 ng/mL) for the indicated times via qRT‒PCR. **E**, **F** Concentrations of essential amino acids (**E** and **F**), including BCAAs (**F**). Untargeted metabolomics analysis was conducted on the samples shown in (**A**). **G** Schematic pathways of BCAAs in cellular metabolism. **H** Intracellular levels of BCAAs were measured in BMDMs stimulated with LPS (100 ng/mL) for the indicated times. Statistical significance was determined via two-tailed Student’s *t* test (**A**, **C**, **E**, and **F**), one-way ANOVA with Tukey’s multiple comparison test (**D**), or two-way ANOVA with Sidak’s multiple comparison test (**H**). LPS, lipopolysaccharide; ECAR, extracellular acidification rate; a.u., arbitrary unit; SC, solvent control; AUC, area under curve; BCAAs, branched chain amino acids; LAT, L-type amino acid transporter; BCAT, branched-chain amino acid transaminase; n.s., not significant. The data are presented as the means ± SD from at least three independent experiments (**A**, **C**–**F**, and **H**). *p < 0.05, **p < 0.01, and ***p < 0.001

Autophagy, a pivotal pathway of cellular homeostasis, involves context-dependent interplay with metabolism [49]. In addition, immune cells undergo metabolic reprogramming in response to the demands imposed by infection or inflammation, in turn influencing innate immune responses [50, 51]. To explore whether UBXN6 impacts immunometabolism during inflammation, we conducted an untargeted metabolomics analysis to investigate metabolic differences between cWT and cKO BMDMs. Initially, myeloid UBXN6 deficiency significantly elevated the lactate/pyruvate ratio (Fig. 5A). Consistently, the extracellular acidification rate (ECAR) was notably increased in cKO BMDMs following LPS stimulation (Fig. 5B, C). The levels of mRNAs encoding *Hif1a* and *Ldha*, key genes of aerobic glycolysis [52], were significantly greater in cKO BMDMs than in WT cells under LPS stimulation (Fig. 5D). These findings suggest that UBXN6 deficiency in macrophages prompts an immunometabolic shift toward aerobic glycolysis.

Untargeted metabolomics analysis revealed that the levels of most essential amino acids were significantly greater in cKO BMDMs than in cWT cells under untreated conditions (Fig. 5E, F). Specifically, under untreated conditions, cKO BMDMs presented notable increases in the levels of BCAAs, including leucine (Leu), isoleucine (Ile), and valine (Val) (Fig. 5F). BCAAs are essential amino acids that have been extensively studied because of their role in protein synthesis and metabolism regulation [53]. BCAAs can accumulate in cells through either increased uptake or impaired catabolism (Fig. 5G), resulting in a metabolic imbalance associated with pathological conditions [54]. Interestingly, the concentration of total BCAAs was significantly elevated in cKO BMDMs before and after LPS treatment at all time points (Fig. 5H). However, the levels of many nonessential amino acids did not differ significantly between cWT and cKO BMDMs before or after LPS stimulation (Supplementary Fig. 15).

Amino acids are transported into cells by solute carriers (SLCs) [55]. L-type amino acid transporters (LATs) primarily deliver neutral amino acids. LAT2/SLC7A8 is prevalent in normal tissues [56]. LAT1/SLC7A5 forms a complex with CD98/SLC3A2, facilitating the plasma membrane trafficking of large neutral amino acids, such as BCAAs and other essential amino acids [57, 58]. To further investigate the mechanisms underlying the changes in specific amino acid profiles in cKO BMDMs, we conducted qRT‒PCR analysis to assess the mRNA expression of *Slc7a5* and *Slc7a8* in cWT and cKO BMDMs treated with LPS for the indicated times. As shown in Supplementary Fig. 16, the mRNA expression levels of *Slc7a5* and *Slc7a8* were significantly greater in the *Ubxn6* cKO BMDMs than in the *Ubxn6* cWT BMDMs, both before and after LPS treatment. These findings suggest that depletion of myeloid UBXN6 drives aerobic glycolysis and BCAA accumulation, likely through dysregulated expression of LATs in macrophages.

### Myeloid UBXN6 is essential for lysosomal biogenesis via the activation of TFEB nuclear translocation

**Fig. 6:**
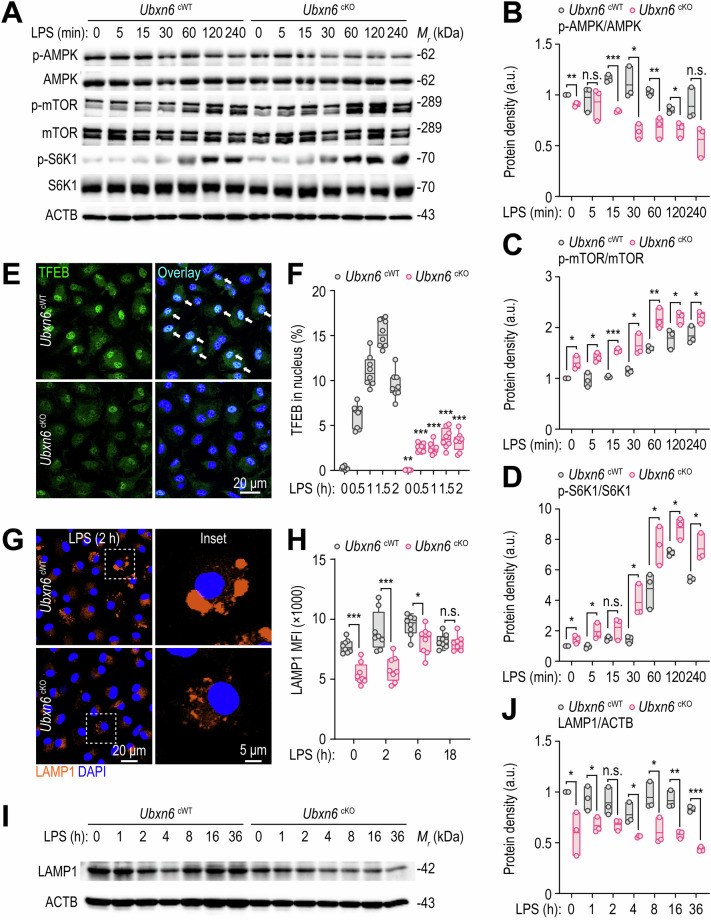
UBXN6 promotes TFEB nuclear translocation-mediated lysosomal activation in macrophages during LPS stimulation. **A**–**D** Phosphorylated and total protein levels associated with the AMPK and mTOR signaling pathways in BMDMs stimulated with LPS (100 ng/mL) for the indicated times; ACTB was used as a loading control (**A**). Relative quantifications are shown for phospho-AMPK normalized to total AMPK (**B**), phospho-mTOR normalized to total mTOR (**C**), and phospho-S6K1 normalized to total S6K1 (**D**). **E**, **F** Confocal microscopy images of immunostained TFEB (green) and DAPI (blue, for nuclei) (**E**) and the percentage of TFEB nuclear translocation (**F**) obtained from BMDMs treated with LPS (100 ng/mL) for the indicated times. The white arrows indicate TFEB in the nucleus. **G** Representative images of BMDMs immunostained with LAMP1 (orange) and DAPI (blue, for nuclei) after stimulation with LPS (100 ng/mL) for 2 h. **H** Mean fluorescence intensities of LAMP1 in BMDMs stimulated with LPS (100 ng/mL) for the indicated periods determined by FIJI software. **I**, **J** Western blot analysis of LAMP1 proteins (**I**) and their relative levels normalized to those of ACTB (**J**) in BMDMs primed with LPS (100 ng/mL) for the indicated periods. Two-tailed Student’s *t* tests (**B**–**D**, **F**, **H**, and **J**) were used to determine statistical significance. LPS, lipopolysaccharide; n.s., not significant; MFI, mean fluorescence intensity. The data represent the means ± SD (**B–D**, **F**, **H**, and **J**) from at least three independent experiments. *p < 0.05, **p < 0.01, and ***p < 0.001

Previous studies have demonstrated that elevated BCAA levels in cells promote the inflammatory response via mTOR complex 1 (mTORC1) activation [59–62]. Given the observed increases in aerobic glycolysis and the accumulation of BCAAs (Fig. 5A–E), we further examined the activation status of the AMP-activated protein kinase (AMPK) and mTOR signaling pathways. Crosstalk between these two pathways is critical in terms of regulating host cell metabolism during infection and inflammation [63, 64]. Under LPS stimulation, phosphorylated AMPK was significantly lower in cKO BMDMs than in cWT BMDMs (Fig. 6A, B). In addition, the levels of phosphorylated mTOR kinase (Fig. 6A, C) and ribosomal protein S6 kinase 1 (S6K1) (Fig. 6A, D) were markedly elevated in cKO BMDMs compared with those in cWT BMDMs following LPS exposure.

mTOR activation triggers the phosphorylation of several critical proteins, including AKT, S6K1, and TFEB [65]. TFEB is a widely studied regulator of lysosome regeneration and biogenesis, influencing various physiological and pathological responses [66]. Given the elevated mTOR kinase-S6K1 level in cKO BMDMs, we investigated whether the nuclear translocation of TFEB was impaired in these cells. Notably, the nuclear translocation of TFEB and the expression of downstream autophagy genes, including *Vps11*, *Uvrag*, *Becn1*, *Dram2*, *Rab7a*, *Lamp1*, *Lamp2*, and *Tfeb*, were significantly reduced in cKO BMDMs following LPS treatment (Fig. 6E, F and Supplementary Fig. 17). Furthermore, LAMP1 protein expression levels were notably decreased in cKO BMDMs both before and after LPS stimulation, as observed via confocal microscopy (Fig. 6G, H) and Western blotting (Fig. 6I, J). These findings suggest that UBXN6 promotes lysosomal biogenesis by activating the mTOR-TFEB signaling pathway.

### Myeloid UBXN6 is required for inflammation control in vivo

**Fig. 7:**
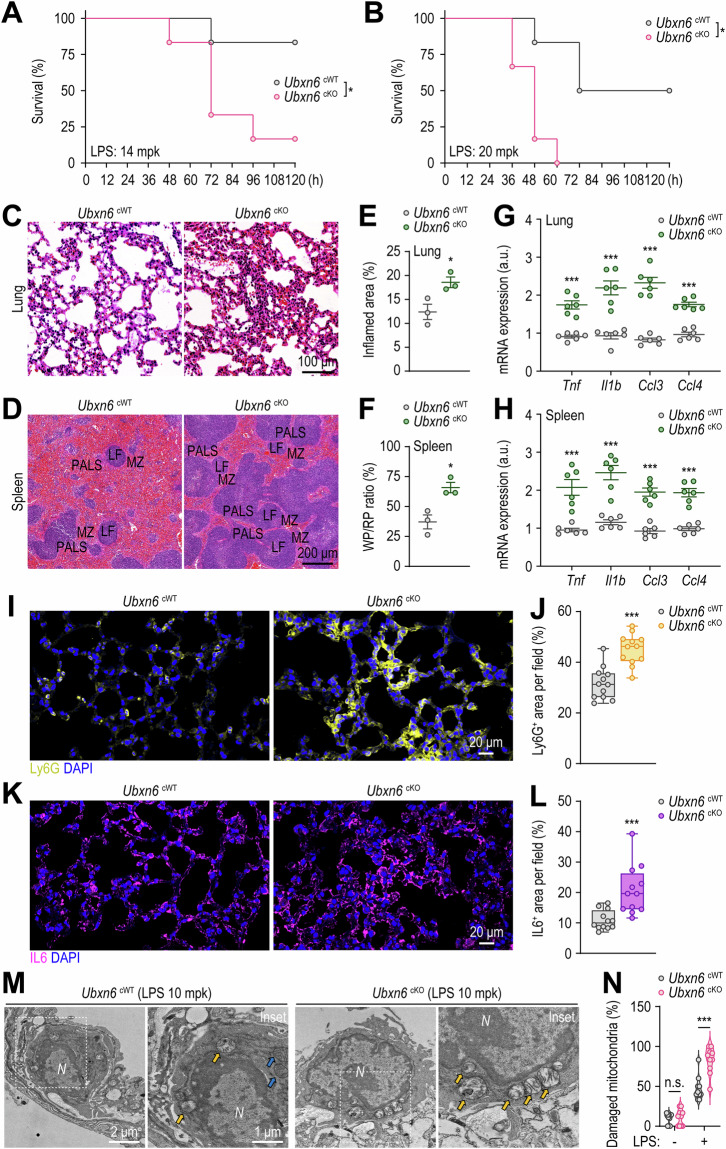
UBXN6 attenuates sepsis-induced mortality and systemic inflammation in vivo. **A**, **B** Survival of *Ubxn6* cWT and cKO mice assessed for 120 h after the administration of LPS (14 mg/kg, *n* = 6) (**A**) or LPS (20 mg/kg, *n* = 6) (**B**). **C**–**F** Hematoxylin and eosin-stained sections of lungs (**C**) and spleen tissues (**D**), with relative quantification of inflamed areas in the lungs (**E**) and the red-to-white pulp ratio in the spleens (**F**) of mice challenged with LPS (14 mg/kg) for 24 h. **G**, **H** Relative mRNA levels of *Tnf*, *Il1b*, *Ccl3*, and *Ccl4* in the lungs (**G**) and spleens (**H**) of mice injected with LPS (14 mg/kg) for 6 h. **I**–**L** Representative confocal microscopy images of Ly6G (**I**)- and IL6 (**K**)-stained cells, with positive areas per field quantified for Ly6G (**J**) and IL6 (**L**), respectively. Paraffin sections of lung tissues from the mice used in (**C**) were immunostained with Ly6G (yellow), IL6 (purple), and DAPI (blue for nuclei). **M**, **N** TEM images (**M**) and quantification of damaged mitochondria (**N**) from alveolar macrophages in ALI model mouse lung tissues. The mice were treated with or without LPS (10 mg/kg) for 24 h before their lungs were analyzed via TEM. The yellow or blue arrows indicate swollen or intact mitochondria, respectively. Statistical significance was determined via either the log-rank (Mantel‒Cox) test (**A**, **B**) or two-tailed Student’s *t* test (**E**–**H**, **J**, **L**, and **N**). PALS, periarteriolar lymphoid sheaths; LF, lymphoid follicle; MZ, marginal zone; WP, white pulp; RP, red pulp; a.u., arbitrary unit; *N*, nuclei; n.s., not significant. The data represent the means ± SEM from 3‒6 biological replicates (**E**–**N**). *p < 0.05 and ***p < 0.001

To determine whether UBXN6 controls inflammation in vivo, both cWT and cKO mice were subjected to LPS challenge. As anticipated, compared with cWT mice, cKO mice displayed increased susceptibility to LPS-induced mortality (Fig. 7A, B). These findings underscore the potentially fatal outcomes of severe inflammation caused by myeloid UBXN6 deficiency. Moreover, compared with those of cWT mice, the lungs and spleens of cKO mice presented increased inflammatory responses. Histochemical staining revealed that UBXN6 deletion resulted in the accumulation of immune cells in the lungs (Fig. 7C) and lymphoid follicles (LFs) of the spleen (Fig. 7D), as well as significant increases in the overall inflamed lung area (Fig. 7E) and the ratio of red-to-white pulp in the spleen (Fig. 7F). The transcriptional levels of genes encoding inflammatory cytokines and chemokines, such as *Tnf*, *Il1b*, *Ccl3*, and *Ccl4*, were significantly elevated in the lungs (Fig. 7G) and spleen (Fig. 7H) of the LPS-injected cKO mice but not in the liver (Supplementary Fig. 18), highlighting the importance of myeloid UBXN6 in controlling inflammation in specific organs. Furthermore, confocal microscopic analysis was used to measure Ly6G and IL6 levels in lung tissues, revealing that, compared with cWT mice, cKO mice presented significantly greater numbers of Ly6G-positive neutrophils (Fig. 7I, J) and IL6-positive cells (Fig. 7K, L). Like LPS, zymosan-induced septic conditions also significantly reduced the survival rate of cKO mice compared with that of cWT mice (Supplementary Fig. 19). We then investigated whether UBXN6 is required to prevent mitochondrial damage caused by acute inflammation in vivo. TEM analysis revealed that, compared with that in cWT mice, the percentage of swollen mitochondria in the lung tissues of the cKO mice with acute lung injury (ALI) was significantly greater (Fig. 7M, N). These findings suggest that UBXN6 in innate immune cells is essential for controlling inflammation in response to various inflammatory stimuli, including dectin-1/TLR2 and TLR4 agonizts.

To further assess the in vivo role of UBXN6 in the context of immunosuppression, a significant immunological feature of severe sepsis [67, 68], we established a murine model of immunosuppression via a two-hit approach involving cecal ligation and puncture (CLP) and secondary bacterial infection [69, 70]. This model is related to increased susceptibility to secondary infections and increased long-term mortality in human sepsis [71]. As shown in Supplementary Fig. 20A and B, upon secondary infection with *Pseudomonas aeruginosa*, CLP-operated cKO mice presented a significantly lower mortality rate and bacterial load than did cWT mice. We also examined the production of proinflammatory cytokines and found that, compared with cWT mice, cKO mice subjected to CLP surgery following *P. aeruginosa* infection presented significantly higher levels of TNF, IL6, and IL1B in the serum, lungs, and spleen (Supplementary Fig. 20C). These findings suggest that myeloid UBXN6 is required for the suppression of inflammatory responses in vivo, thereby increasing susceptibility to secondary bacterial infection during immunosuppression.

## Discussion

“Immunosuppression-related research” has been a popular research topic in the field of sepsis over the last two decades [72]. Unresolved opportunistic infections, which are caused mainly by immunosuppression, trigger high mortality in sepsis patients [73, 74]. Through transcriptome analysis of sepsis patients, we identified significant immune suppression in the SP subgroup, as evidenced by the reduced levels of key cytokines such as *IL1B* and *CCL4* in PBMCs. Sepsis-induced immunosuppression manifests via diverse mechanisms, including upregulation of regulatory T cells, negative modulation of TLR signaling pathways, apoptosis of immune cells, and increased expression of negative costimulatory molecules [75]. When we compared the ATG DEGs between SR patients and SP patients, we observed a notable increase in *UBXN6* expression in the latter. Notably, *UBXN6* expression was negatively correlated with the expression levels of inflammatory genes but positively correlated with the expression levels of *FOXO3*, *PINK1*, and *BNIP3L*. Furthermore, there was a significant increase in *UBXN6* expression across two additional cohorts sourced from public databases. These findings suggest that increased expression of *UBXN6* in SP patients may exacerbate immunosuppression, thereby impairing innate immune responses during sepsis.

Our data also reveal the negative regulatory role of UBXN6 in response to inflammatory signals and its impact on the expression of *Tnfaip3*, a deubiquitinating enzyme, which in turn suppresses inflammatory responses [76]. Although our data shed light on the regulatory function of UBXN6 in terms of modulating the expression of genes involved in inflammatory signaling pathways, a previous study revealed that UBXN1 suppressed TNF-mediated NF-κB activation through interactions with cellular inhibitors of apoptosis-related proteins [77]. These results underscore the notion that several p97 cofactors play crucial roles as negative regulators within NF-κB-mediated inflammatory signaling pathways. This may reflect interactions among cofactors or the regulation of gene expression levels of various signaling partners downstream of innate immune receptor engagement or cytokine receptor activation.

Our scRNA-seq analysis revealed that monocytes/macrophages were the primary cell types expressing UBXN6. Notably, UBXN6 plays a crucial role in the induction of autophagy in macrophages in response to various autophagic stimuli, including inflammatory signals, mTOR inhibition, and AMPK activation. Specifically, LC3 puncta formation was significantly compromised in UBXN6-deficient murine macrophages and human primary monocytes. Additionally, mitophagy induction was impaired in cKO BMDMs. A recent study highlighted the involvement of UBXN6 in Parkin-dependent mitophagy; UBXN6 is translocated to mitochondria and facilitates the recruitment of p97 via bipartite binding of a motif composed of the N-terminal VIM and PUB domains, in turn activating mitophagic flux [78]. In addition, UBXN6, which serves as a promitophagic cofactor for p97, mitigated impaired Parkin recruitment in UBXN1-deficient HeLa cells [79]. Importantly, we found that LC3 puncta formation, induced by either a small molecule inhibitor or an enhancer of p97, was significantly compromised in cKO BMDMs. These findings suggest that UBXN6-mediated autophagy induction occurs independently of p97 activity.

UBXN6-mediated autophagy is associated with the transcriptional activation of both autophagic and lysosomal genes via the nuclear translocation of TFEB and the FOXO3 signaling pathway. TFEB, a key transcription factor involved in lysosomal biogenesis and autophagy activation, undergoes nuclear translocation after mTOR pathway inhibition, intracellular calcium influx, and the suppression of ERK1/2 [80, 81]. There is substantial evidence connecting AMPK activation with FOXO3 signaling. AMPK activation directly enhances FOXO3 transcriptional activity [82]. Additionally, AMPK promotes the dephosphorylation and nuclear translocation of FOXO3 by inhibiting AKT activation [83]. The AMPK‒FOXO3 axis is also recognized for its role in the nutrient-sensing pathway [84]. Considering the critical roles of both mTOR and AMPK in regulating energy metabolism [85], along with our findings of decreased AMPK and increased mTOR signaling in cKO BMDMs, our results strongly suggest that UBXN6-mediated autophagy induction is closely linked to immunometabolic regulation by controlling the AMPK‒mTOR axis in macrophages.

We observed significant reductions in the expression levels of SEL1L and SYVN1, which are ERAD genes and proteins, in UBXN6-deficient macrophages compared with those in cWT macrophages after inflammatory stimulation. The well-known ERAD process plays a crucial role in eliminating misfolded ER protein substrates. These substrates are retrotranslocated to the cytosol, undergo polyubiquitination, and are degraded in the proteasome [86]. Specifically, the p97 ATPase complex extracts ERAD substrates and transports them to the proteasome for disposal [86]. In addition, ERAD deficiency exacerbates the permeability and dysfunction of the mitochondrial outer membrane, thereby increasing mtROS accumulation [46]. In addition to the compromised induction of canonical autophagy, the decrease in ERAD observed in UBXN6-deficient macrophages may contribute to increased mtROS production, thereby promoting the upregulation of proinflammatory responses. One study demonstrated that the silencing of UBXN6 did not affect the overall level of protein degradation or the activity of the ERAD pathway [14]. Consequently, the role of UBXN6 in regulating ERAD appears to be unique to macrophages and could modulate the exaggeration of inflammatory processes by influencing mitochondrial homeostasis. Furthermore, cellular ROS levels and ER stress were significantly elevated in cKO BMDMs during LPS-induced inflammation. Defective ERAD in cKO macrophages may impair the control of protein quality and quantity, thereby increasing ER stress in these cells. Given that cellular ROS levels are increased in cKO BMDMs, UBXN6 deficiency alters redox homeostasis, disrupting the protein folding pathway and exacerbating ER stress.

Our metabolomic analysis revealed increased aerobic glycolysis and specific changes in the levels of certain amino acids in UBXN6-deficient macrophages. Notably, the levels of several amino acids, including BCAAs, were elevated, even in untreated UBXN6-deficient macrophages, compared with those in cWT cells. These findings partially support previous research indicating that pharmacological depletion of p97 activity induces proteotoxic stress in various cancer cells, disrupting intracellular amino acid levels [87]. Increased amino acid levels may increase signaling that triggers mTOR kinase activation, thereby suppressing TFEB nuclear translocation in macrophages [88, 89]. We also found that the mRNA expression levels of *Slc7a5* and *Slc7a8* were significantly greater in cKO BMDMs than in cWT BMDMs. These data strongly suggest that the elevated levels of BCAA in cKO BMDMs may be linked to the aberrant expression of LATs, which are known to absorb BCAAs into cells and activate mTORC1 pathways [60, 61, 90]. Future studies are warranted to elucidate how UBXN6-dependent signals may regulate the gene expression of specific amino acid transporter subunits in macrophages.

In summary, our data reveal a previously unidentified role for myeloid UBXN6 in orchestrating autophagy, ERAD, and immunometabolism, thus influencing mitochondrial homeostasis and inflammation in macropahges. In addition, our findings underscore the importance of the role of UBXN6 in terms of immunosuppression during sepsis, suggesting the potential efficacy of novel therapeutics targeting UBXN6 in patients with systemic inflammation.

## Materials and methods

### Patient characteristics

Twelve patients with sepsis or septic shock were included in the study. Community-acquired pneumonia was the most common cause in this study, accounting for eight cases (66.7%). Supplementary Table 2 summarizes the patients’ demographic and laboratory characteristics. There were four septic shock patients (33.3%). Eight patients (66.7%) required mechanical ventilation, and one had a tracheostomy. The 28-day mortality rate was 25%. Eight patients had comorbidities, six of whom had more than two. The comorbidities had no significant effect on ICU mortality. Eight patients (66.7%) had causative pathogens, with *Escherichia coli* being the most common. Supplementary Table 3 compares the characteristics of patients who recovered within 48 h to those of patients who did not recover within 48 h of septic shock. Univariate analysis revealed significant differences in survival rates between the two groups.

### Extraction of RNA or mitochondrial DNA (mtDNA) contents

Total RNA was extracted via TRIzol reagent (Invitrogen, Waltham, MA, USA, 15596026) according to the manufacturer’s instructions. cDNA synthesis was carried out via reverse transcriptase premix (ELPIS Biotech, Daejeon, South Korea, EBT-1515) following the manufacturer’s instructions. To determine the mtDNA copy number, total DNA was extracted from BMDMs via a DNeasy blood and tissue kit (Qiagen, Hilden, Germany, 69504) following previous methods [91, 92].

### Bulk RNA-seq of human-derived materials

After total RNA was extracted from each sample, the mRNA was enriched with oligo(dT) magnetic beads, and the rRNA was removed. After the addition of fragmentation buffer, the mRNA was fragmented into short fragments (approximately 200 bp), and the first-strand cDNA was subsequently synthesized via random hexamer primers with the mRNA fragments used as templates. Buffer, dNTPs, RNase H, and DNA polymerase I were added to synthesize the second strand. The double-strand cDNA was purified with a QIAquick Gel Extraction Kit & PCR Purification Kit (Qiagen, 28704 and 28104, respectively) and washed with EB buffer (elution buffer; Qiagen, 19086) for end repair and poly(A) addition. Finally, sequencing adapters were ligated to the fragments. The required fragments were purified via agarose gel electrophoresis and enriched via PCR amplification. The library products were assessed with a BioAnalyzer 2100 (Agilent Technologies, Santa Clara, CA, USA). The resulting cDNA libraries were sequenced via the HiSeq 4000 platform (Illumina, San Diego, CA, USA), generating approximately 1.44 billion paired-end reads of 151 nucleotides in length. The raw RNA-seq data have been deposited in the NCBI SRA database with the following accession numbers: SRX8138400--SRX8138419 under BioProject PRJNA625581 and SRR28762191--SRR28762194 under BioProject PRJNA1102979.

### Bulk RNA-seq analysis of human-derived materials

To obtain high-quality clean reads for transcript analysis, all raw sequence reads were preprocessed via Trimmomatic (v0.36) [93] to trim the adapter sequences and remove low-quality sequences. The remaining clean reads for each sample were aligned independently to the human reference genome (hg38) via HISAT2 (v2.1.0) [94]. To assemble and quantify the transcripts, the resulting aligned reads and human annotation data were input into Cufflinks (v2.2.1) [95]. We subsequently merged the transcriptome assemblies from each sample via the Cuffmerge script (v2.2.1) implemented in Cufflinks and applied Cuffdiff (v2.2.1) [96] with default parameters for the identification of DEGs. In this study, we defined genes as differentially expressed genes with an FDR of less than 5%. The human reference genome and annotation data were obtained from the UCSC genome browser (https://genome.ucsc.edu), and the data for visualization were generated via R (R Development Core Team, Vienna, Austria). Unless otherwise stated, the unit of expression level in our analysis was fragments per kilobase of exon per million fragments mapped (FPKM). Gene Ontology (GO) enrichment analysis was performed with the DAVID (v6.8) functional annotation analysis tool (https://david.ncifcrf.gov) [97].

### Single-cell RNA-seq (scRNA-seq)

We performed library construction via 10× Chromium Single Cell 3’ reagent kits v3.1 and sequenced the libraries on the NovaSeq 6000 platform (Illumina). The initial sequencing data were processed and converted into FASTQ files via the Cell Ranger pipeline. We adhered to the standard sequence protocol recommended by 10× Genomics, which involves trimming the barcode and unique molecular identifier (UMI) ends at 26 base pairs and the mRNA ends at 98 base pairs. Following this preprocessing step, the resulting FASTQ files were aligned to the human reference genome (GRCh38). We subsequently employed Cell Ranger for preliminary data analysis, resulting in the generation of a data file comprising a barcode table, a gene table, and a gene expression matrix. To analyze the scRNA-seq data, we utilized R with Seurat (v.4.0.5) [98] to process the single-cell read counts obtained from each sample. To ensure data quality, we filtered out cells on the basis of the following criteria: cells with UMI counts per cell less than 500, cells with genes detected per cell less than 300, and cells with a mitochondria ratio exceeding 10%. Subsequently, filtered Seurat objects were integrated, and data normalization was performed via SCTransform to correct for batch effects originating from different samples. Next, highly variable genes were identified via the FindVariableFeatures function within Seurat. These identified genes were then subjected to principal component analysis for linear dimension reduction. Subsequently, cell clusters were identified via the FindClusters function, with the resolution parameter set to 0.05. To visualize the cell clusters, we employed t-distributed stochastic neighbor embedding (tSNE). The scRNA-seq data and analysis script are available at https://github.com/tjdrnjsqpf/UBXN6_scRNA-seq.

### Mice

The mice used in individual experiments were age- (6–8 weeks) and sex-matched. *Ubxn6*^flox/flox^ mice were purchased from Cyagen Biosciences (Jiangsu, China; CKOCMP-66530-Ubxn6-B6J-VA). *Lys2*^Cre^ mice were kindly provided by Dr. C.-H. Lee (Korea Research Institute of Bioscience and Biotechnology). The mice were maintained under specific pathogen-free conditions. The animal experiments and handling were performed following the ethical guidelines of the Chungnam National University College of Medicine and were approved by the Institutional Animal Care and Use Committee (202109A-CNU-180; Daejeon, South Korea) and the South Korean Food and Drug Administration.

### Genotyping

Homozygous targeted mice from heterozygous breeding pairs were generated. Semiquantitative PCR was conducted by using Power S Taq Premix (HKGenomics, Daejeon, South Korea, 11201). The tissue-specific gene deletion was confirmed by the following primer sequences (5 to 3 primers): *Ubxn6* conditional wild-type (*Ubxn6* cWT); forward, 5-GAG GAA CAT GGA GGT TCA AAG GA-3; reverse, 5- CAG TGC AGT TCA GAG GCA GGT T-3. *Ubxn6* conditional knockout (*Ubxn6* cKO); forward, 5-GAG GAA CAT GGA GGT TCA AAG GA-3; reverse, 5- AAG TCT CGT GTT GAA CTC CTT ACA-3. The fragment size was 431 bp for the *Ubxn6* cWT allele and 363 bp for the *Ubxn6* cKO allele.

### Isolation, cultivation, and treatment of cells

Human PBMCs were isolated from heparinized venous blood by density sedimentation over Ficoll-Hypaque (Lymphoprep; Alere Technologies, Oslo, Norway, 07851). The cells were incubated for 1 h at 37 °C, and nonadherent cells were removed by pipetting off the supernatant. Adherent monocytes were then resuspended in Roswell Park Memorial Institute 1640 medium (Corning, 1 Riverfront Plaza, NY, USA, 10-040-CVRC) containing 5% human serum (Sigma‒Aldrich, St. Louis, MO, USA, H3667) and 1% L-glutamine. Primary BMDMs were obtained as follows. Bone marrow cells were harvested from the femurs and tibias of 6–8-week-old mice and cultured in Dulbecco’s modified Eagle’s medium (DMEM; Lonza, Walkersville, MD, USA, BE12–604 F) supplemented with 10% fetal bovine serum (Gibco, Grand Island, NY, USA, 16000–044) and a penicillin‒streptomycin‒amphotericin B mixture (Lonza, 17--745E) containing 25 ng/mL macrophage colony-stimulating factor (R&D Systems, Minneapolis, MN, USA, 416--ML-050) at 37 °C in 5% CO_2_ for 4‒5 days for differentiation. LPS (InvivoGen, San Diego, CA, USA, tlrl-eblps), zymosan (InvivoGen, tlrlzyn), ATP (Sigma‒Aldrich, A5394), nigericin (Sigma‒Aldrich, SML1779), AICAR (Sigma‒Aldrich, A9978), Baf-A1 (Sigma‒Aldrich, b1793), DBeQ (Selleckchem, Houston, TX, USA, S7199), SMER28 (Tocris Bioscience, Bristol, UK, 307538-42-7), and MitoTEMPO (Sigma‒Aldrich, SML0737) were added at the indicated concentrations and times for individual experiments.

### Bulk RNA-seq and analysis of mouse-derived macrophages

Total RNA from mouse BMDMs was extracted via TRIzol according to the manufacturer’s instructions. An Agilent TapeStation 4000 system (Agilent Technologies) was used to assess RNA quality, and an ND-2000 spectrophotometer (Thermo Fisher Scientific, Waltham, MA, USA) was used for RNA quantification. The library was constructed following the guidelines provided by the manufacturer and involved the use of the QuantSeq 3’ mRNA-Seq Library Prep Kit (Lexogen, Vienna, Austria, 225.96). Reverse transcription was performed using an oligo-dT primer that contained an Illumina-compatible sequence at the 5’ end, which hybridized to the RNA. Following the degradation of the RNA template, the initiation of second-strand synthesis occurred via a random primer with an Illumina-compatible linker sequence attached to its 5’ end. The double-stranded library was cleansed via magnetic beads. The library was amplified by incorporating the full adapter sequences necessary for cluster creation. The completed library was purified to remove PCR components. The high-throughput sequencing process was performed as follows: 75 single-end sequencing was performed at 10 M. The sequencing machine used was a NextSeq 550 (Illumina). The QuantSeq 3’ mRNA-Seq reads were aligned via Bowtie2, as described by Langmead and Salzberg in 2012. Bowtie2 mapped the reads to the reference genomes mm10 and UCSC, and the read counts were calculated via Bedtools (Quinlan AR, 2010). The read count data were normalized via the TMM + CPM normalization approach via EdgeR in R (R Development Core Team, Vienna, Austria) via Bioconductor [99]. The normalized counts were used for Z score calculation.

### Quantitative real-time polymerase chain reaction (qRT‑PCR)

qRT‒PCR was performed via SYBR Green Master Mix (Qiagen, 204074) in the Rotor-Gene Q 2plex system (Qiagen). The 2^ΔΔ^ threshold cycle method was used for data analysis. Glyceraldehyde-3-phosphate dehydrogenase (*Gapdh*) was used for normalization. The sequences of primers used in this study are listed in Supplementary Table 4.

### Enzyme-linked immunosorbent assay (ELISA)

Cell supernatants from BMDMs were stored at -80 °C until ELISA. A mouse TNF-α ELISA kit (BD Biosciences, Franklin Lakes, NJ, USA, 558534), an IL-6 ELISA kit (BD Biosciences, 555240), an IL-1B ELISA kit (Invitrogen, 88-7013-88), and a human TNF-α ELISA kit (BD Biosciences, 555212) were used for the ELISA. Both the ELISA experiment and data analysis were conducted in accordance with the manufacturer’s protocols.

### Western blotting

The cells were washed with cold phosphate-buffered saline (PBS) and lysed in Laemmli’s 5× Sample Buffer (ELPIS Biotech, EBA-1052) diluted with radioimmunoprecipitation assay (RIPA; LPS solution, Daejeon, South Korea, CRB002) buffer supplemented with protease inhibitor cocktail (Roche, Basel, Switzerland, 11836153001) and phosphatase inhibitor cocktail (Roche, 4906837001) to obtain protein samples. Equal amounts of protein were boiled for 10 min on a heating block and cooled on ice for 10 min. Denatured protein samples were then subjected to sodium dodecyl sulfate‒polyacrylamide gel electrophoresis. The separated proteins were transferred to polyvinylidene difluoride (PVDF; Millipore, Billerica, MA, USA, IPVH0001) or nitrocellulose (NC; Pall Corporation, NY, USA, 66485) membranes. The membranes were incubated with primary antibodies at 4 °C overnight, followed by incubation with the corresponding horseradish peroxidase-conjugated secondary antibodies. The SuperSignal West Femto Maximum Sensitivity Substrate (Thermo Fisher Scientific, 34095) was used for the visualization of appropriate signals in the iBright 750 imaging system (Thermo Fisher Scientific, CL750). The primary antibodies used were as follows: anti-phospho-NF-κB p65 (1:2000; Cell Signaling Technology, Danvers, MA, USA, 3033), anti-phospho-AKT (1:1000; Cell Signaling Technology, 9271), anti-phospho-JNK (1:0000; Cell Signaling Technology, 4668), anti-phospho-p44/42 MAPK ERK1/2 (1:2000; Cell Signaling Technology, 9101), anti-phospho-p38 (1:2000; Cell Signaling Technology, 4511), anti-beta-ACTB (1:2000; Santa Cruz Biotechnology, Dallas, TX, USA, sc-47778), anti-mature-IL1B (1:1000; Cell Signaling Technology, 12507), anti-mature-CASP1 (1:2000; Cell Signaling Technology, 2225), anti-NLRP3 (1:1000; Adipogen Life Sciences, San Diego, CA, USA, AG-20B-0014), anti-pro-IL1B (1:1000; Cell Signaling Technology, 12242), anti-pro-CASP1 (1:2000; Santa Cruz Biotechnology, sc-56036), anti-UBXN6 (1:1000; Abcam, ab103651), anti-CASP11 (1:1000; Cell signaling Technology, 14340), anti-LC3 (1:1000; Sigma‒Aldrich, L8918), anti-SEL1L (1:1000; Abcam, Cambridge, UK, ab78298), anti-SYVN1 (1:1000; Thermo Fisher Scientific, pa5-100081), anti-phospho-AMPK (1:1000; Cell Signaling Technology, 2535), anti-AMPK (1:1000; Cell Signaling Technology, 2532), anti-phospho-mTOR (1:1000; Cell Signaling Technology, 5536), anti-mTOR (1:1000; Cell Signaling Technology, 2983), anti-phospho-S6K1 (1:1000; Cell Signaling Technology, 9205), anti-S6K1 (1:1000; Cell Signaling Technology, 2708), and anti-LAMP1 (1:1000; Cell Signaling Technology, 99437). The secondary antibodies used were as follows: anti-rabbit IgG-horseradish peroxidase (HRP) linked (1:2000; Cell Signaling Technology, 7074), anti-mouse IgG-HRP linked (1:2000; Cell Signaling Technology, 7076), and anti-rat IgG-HRP linked (1:2000; Cell signaling Technology, 7077).

### Immunofluorescence staining and confocal microscopic analysis

The cells were fixed in 4% paraformaldehyde (PFA) at room temperature (RT) overnight and permeabilized with 0.25% (v/v) Triton X-100 (Sigma‒Aldrich, T8787) in PBS for 10 min at RT. The cells were washed with PBS and then incubated with secondary antibodies for 2 h at RT. The primary antibodies used were as follows: anti-LC3 (1:400; MBL Co., LTD., Tokyo, Japan, PM036), anti-LAMP1 (1:400; Santa Cruz Biotechnology, sc-19992), anti-RELA/NF-κB (1:400; Santa Cruz Biotechnology, sc-8008), and anti-TFEB (1:400; Bethyl Laboratories, Montgomery, TX, USA, A303--673A). The secondary antibodies used were as follows: Alexa Fluor 488-conjugated anti-rabbit IgG (1:400; Invitrogen, A11006), Alexa Fluor 488-conjugated anti-rat IgG (1:400; Invitrogen, A11008), and Alexa Fluor 484-conjugated anti-mouse IgG (1:400; Invitrogen, A11029). For mitophagy analysis, the cells were stained with 100 nM MitoTracker Deep Red (Thermo Fisher Scientific, M22426) in prewarmed DMEM for 30 min at 37 °C, fixed in 4% PFA, permeabilized with 0.25% (v/v) Triton X-100, and then stained with anti-LC3 polyclonal antibodies. For the measurement of mtROS or cellular ROS, the cells were incubated with 1 μM MitoSOX Red (Thermo Fisher Scientific, M36008) or 20 μM DCF-DA (Calbiochem, Darmstadt, Germany, 287810) in DMEM for 20 min at 37 °C, fixed in 4% PFA, and permeabilized with 0.25% (v/v) Triton X-100. These cells were then mounted with Fluoromount-G, with DAPI (Invitrogen, 00-4595-52). For the immunostaining of in vivo paraffin sections, mouse lung tissues were harvested, fixed with 10% formalin, and embedded in paraffin wax. Paraffin sections (3 μm) were cut and immunostained with anti-Ly6G monoclonal antibodies (1:400; Bio X Cell, Lebanon, NH, USA, BE0075), anti-IL6 monoclonal antibodies (1:400; Santa Cruz Biotechnology, sc-57315), and appropriate secondary antibodies. ProLong™ Gold Antifade Mountant with DAPI (Invitrogen, P36931) was used for mounting. After 2 days of mounting, the images were visualized and captured via confocal microscopy (Carl Zeiss A.G., Baden-Württemberg, Germany, LSM 900 with Airyscan 2) and accompanying software (Zen blue edition; Carl Zeiss A.G.). The image capture parameters, such as excitation, emission, and exposure time, were kept constant. Each condition was assayed in quadruplicate, and at least 50–100 cells per field were counted. FIJI software was used to quantify LC3 puncta, fluorescence intensities, colocalization tests, and nuclear translocation levels in images via plugins, such as measurements, Pearson correlation coefficients, or EzColocalization.

### Inflammasome analysis

BMDMs were primed with LPS (100 ng/mL) in Opti-MEM (Gibco, 31985-070) for 4 h and then stimulated with 5 mM ATP (Sigma‒Aldrich, A5394) or 10 µM nigericin sodium salt (Sigma‒Aldrich, SML1779) for 45 min to activate the canonical inflammasome or transfected with 2 μg/mL LPS using Xfect polymer (Clontech Laboratories, Mountain View, CA, USA, 631318) according to the manufacturer’s instructions for the indicated times to activate the noncanonical inflammasome. The supernatants were collected and centrifuged at 4 °C to eliminate debris. Proteins were precipitated from the supernatant via StrataClean Resin (Agilent Technologies, 400724). Pull-down proteins were resuspended in 1× sample buffer diluted in RIPA buffer, boiled for 10 min, and subjected to Western blotting.

### Transmission electron microscopy (TEM) analysis

The samples were sequentially fixed in 3% glutaraldehyde and 1% osmium tetroxide, cooled on ice for 1 h, washed with 0.1 M cacodylate buffer (pH 7.2) containing 0.1% CaCl_2_, and dehydrated in an ethanol and propylene oxide series. Next, the samples were embedded in the Epon 812 mixture and polymerized at 60 °C for 36 h. Using a ULTRACUT UC7 ultramicrotome (Leica Biosystems, Wetzlar, Germany), 70 nm thick sections were cut and mounted on 75-mesh copper grids. The sections were counterstained with uranyl acetate and lead citrate for 10 min and 7 min, respectively, and examined via KBSI Bio-High Voltage EM (JEM1400 Plus at 120 kV and JEM-1000BEF at 1000 kV; JEOL Ltd., Tokyo, Japan).

### Production and transduction of lentiviral short hairpin RNA (shRNA)

shRNA was produced via pLKO.1-based target shRNA plasmids. The plasmids pRSV-Rev (Addgene, Watertown, MA, USA, 12253), pMDLg/pRRE (Addgene, 12251), pMD2. G (Addgene, 12259), and *UBXN6* or *Foxo3* shRNA plasmids (Santa Cruz Biotechnology, sc-97428-SH or sc-37888-SH, respectively) were purchased for viral packaging. To produce the lentivirus, all the above plasmids were transfected into human embryonic kidney 293 T (HEK293T) cells via the Lipofectamine 2000 (Invitrogen, 11668-019) system for 72 h. Finally, the media supernatant containing the lentivirus was collected, centrifuged, and filtered before being stored at -80 °C. For lentiviral infection, BMDMs or human primary monocytes cultured in 48- or 96-well plates were infected with a lentiviral vector at a multiplicity of infection of 10 for 36 h, followed by subsequent treatment.

### Untargeted metabolomics analysis

Intracellular metabolic extracts were prepared from 2 × 10^6^ cells with methanol containing internal standard solution (Human Metabolome Technologies, H3304-1002) and analyzed via capillary electrophoresis (CE)-connected electrospray ionization (ESI)-time-of-flight mass spectrometry (TOFMS) and a CE-tandem mass spectrometry (MS/MS) system (Human Metabolome Technologies, CARCINO-SCOPE). The culture medium was removed from the 60-mm dish, and the cells were washed twice in 5% mannitol solution (10 mL first and then 2 mL). The cells were then treated with 800 μL of methanol and 550 μL of Milli-Q water containing an internal standard solution. The metabolite extract was transferred into a microfuge tube and centrifuged at 2300 × g and 4 °C for 5 min. Next, the upper aqueous layer was centrifugally filtered through a Millipore 5-kDa cutoff filter at 9100 × g and 4 °C for 120 min to remove proteins. The filtrate was centrifugally concentrated and resuspended in 50 μL of Milli-Q water for CE-MS analysis. The concentrations of the metabolites were calculated by normalizing the peak area of each metabolite with respect to the area of the internal standard and by using standard curves, which were obtained via three-point calibrations.

### Extracellular acidification rate (ECAR) analysis

ECAR measurements were performed via a Seahorse Bioscience XF24 Analyzer (Agilent Technologies). BMDMs were seeded at 2.5 × 10^5^ cells per well in an XF24 cell culture microplate (Agilent Technologies, 100777-004), incubated overnight at 37 °C, and subsequently treated. Before analysis, 590 μL of assay medium (XF base medium containing 1 mM L-glutamine, 1 mM sodium pyruvate and 25 mM glucose [pH 7.4]) was added to each well, and the plate was incubated in a non-CO_2_ incubator for 1 h at 37 °C. The XF24 Biosensor Cartridge was activated for 24 h in XF calibrant solution (1 mL/well; Agilent Technologies, 100840--000) at 37 °C in a non-CO_2_ incubator. The basal ECAR was measured, and sequential injections of the following reagents were performed at 37 °C: the ATPase inhibitor oligomycin A (2 μg/mL; Sigma‒Aldrich, 75351), the uncoupler carbonyl cyanide 3-chlorophenylhydrazone (CCCP, 5 µM; Sigma‒Aldrich, C2759) and the mitochondrial complex I inhibitor rotenone (2 µM; Sigma‒Aldrich, 557368).

### Measurement of branched-chain amino acids (BCAAs)

BMDMs were seeded at 5 × 10^5^ cells per well in 24-well plates (SPL Life Sciences, Pocheon, South Korea, 30024), incubated overnight at 37 °C, and stimulated with vehicle or LPS (100 ng/mL) for the indicated times. BCAAs were extracted via a BCAA assay kit (Abcam, ab83374) according to the manufacturer’s instructions, and the optical density (OD) at 450 nm was measured via a microplate reader (BMG Labtech, Ortenberg, Germany; LUMIstar Omega).

### Experimental mouse models of sepsis and acute lung injury (ALI)

To establish the LPS- or zymosan-induced sepsis model, the mice were intraperitoneally injected with LPS (Sigma‒Aldrich, L3755) at a dose of 14 or 20 mg/kg or with zymosan (InvivoGen, tlrlzyn) at a dose of 300 mg/kg. The mice were then observed for survival at 12 h intervals, and the overall survival rate was calculated until 5 days postinjection. To collect the tissues, the LPS-injected mice (14 mg/kg) were euthanized after 6 h. For the ALI model, the mice were anesthetized and intranasally administered LPS (10 mg/kg). Twenty-four hours after the injection, the lung tissues were collected and processed. To establish an immunosuppression model via a two-hit approach, the mice were subjected to CLP or a sham operation. After 24 h, the mice were intravenously injected with the *P. aeruginosa* reference strain PAO1 (3 × 10^6^ CFU/head). At 4 h postinfection, blood, lung, and spleen tissues were collected to assess the bacterial burden and the levels of proinflammatory cytokines and chemokines. The remaining mice were then observed for survival at 12 h intervals, and the overall survival rate was calculated until 5 days post infection.

### Histology and immunofluorescence

Lung and spleen tissues were harvested and fixed in 10% formalin. After fixation, the tissues were embedded in paraffin wax. The paraffin-embedded tissues were cut into 4 μm thick sections. These sections were then stained with hematoxylin and eosin to visualize the tissue morphology. Images of the stained tissue sections were captured via light microscopy. Whole fields of tissue were scanned, and the inflamed areas in the lungs, as well as the ratio of red-to-white pulp in spleen tissues, were quantified. The percentage of inflamed area per whole field of tissue was determined via FIJI software.

### Statistical analysis

Statistical analysis was conducted via Prism 8.0 for Windows (GraphPad Software Inc., San Diego, CA, USA). Two-tailed Student’s *t* test was used to compare two groups, and one-way ANOVA with Tukey’s multiple comparison test or two-way ANOVA with Sidak’s multiple comparison test was used for three or more groups. The log-rank (Mantel‒Cox) test was used to determine the survival rate. The data are presented as the means ± standard deviations (SDs) or ± standard errors of the means (SEMs). Statistical significance is indicated as *p < 0.05, **p < 0.01, and ***p < 0.001.

## Supplementary information

Supplementary Figure

Supplementary Table 1

Supplementary Table 2

Supplementary Table 3

Supplementary Table 4

Uncropped Western blots

## Data Availability

The raw RNA-seq data have been deposited in the NCBI SRA database with the following accession numbers: SRX8138400-SRX8138419 under BioProject PRJNA625581 and SRR28762191-SRR28762194 under BioProject PRJNA1102979.
