# Supplementary material for: Ubiquitin regulatory X (UBX) domain-containing protein 6 is essential for autophagy induction and inflammation control in macrophages
Source: Cell Mol Immunol. 2024 Oct 23;21(12):1441–58. doi: 10.1038/s41423-024-01222-1 (PMC11606977; doi:10.1038/s41423-024-01222-1)
Supplement: Supplementary file 1 — Supplementary Figure [file 41423_2024_1222_MOESM1_ESM.docx]

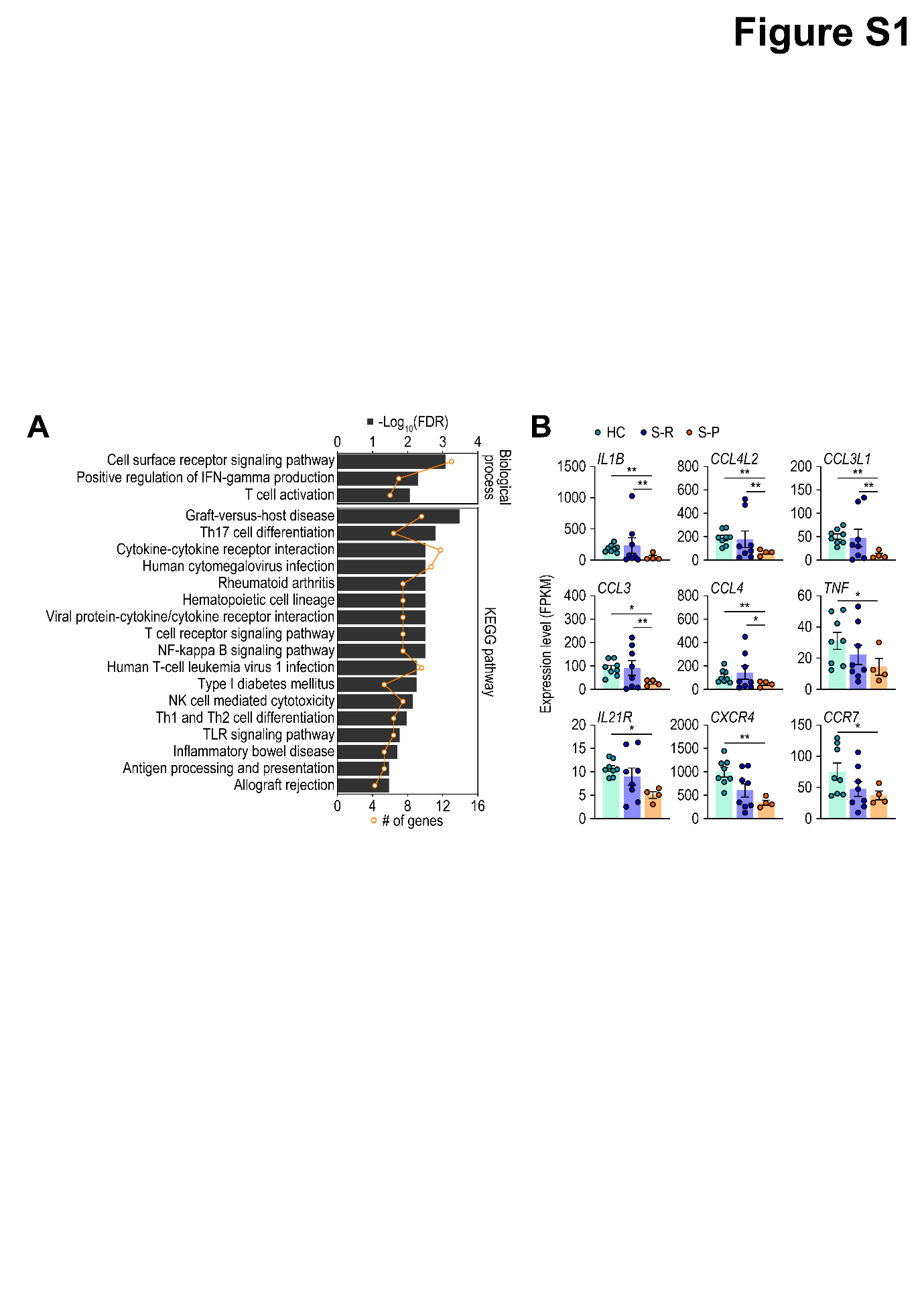


**Figure S1. Immunosuppressive profiles were observed in PBMCs from sepsis patients with a poor prognosis. A** Results of GO and KEGG pathway enrichment analysis based on genes downregulated in SP. **B** The expression of nine genes downregulated in SP that are associated with cytokines or cytokine receptors. Adjusted *p*-values (*q*-value) were estimated by Cuffdiff package implemented in Cufflinks (v.2.1.1). FDR, false discovery rate; KEGG, Kyoto Encyclopedia of Genes and Genomes; FPKM, fragment per kilobase of transcript per million mapped reads; HC, healthy controls; SP, patients with a poor prognosis; SR, patients who had recovered. **p* < 0.05 and ***p* < 0.01.

**
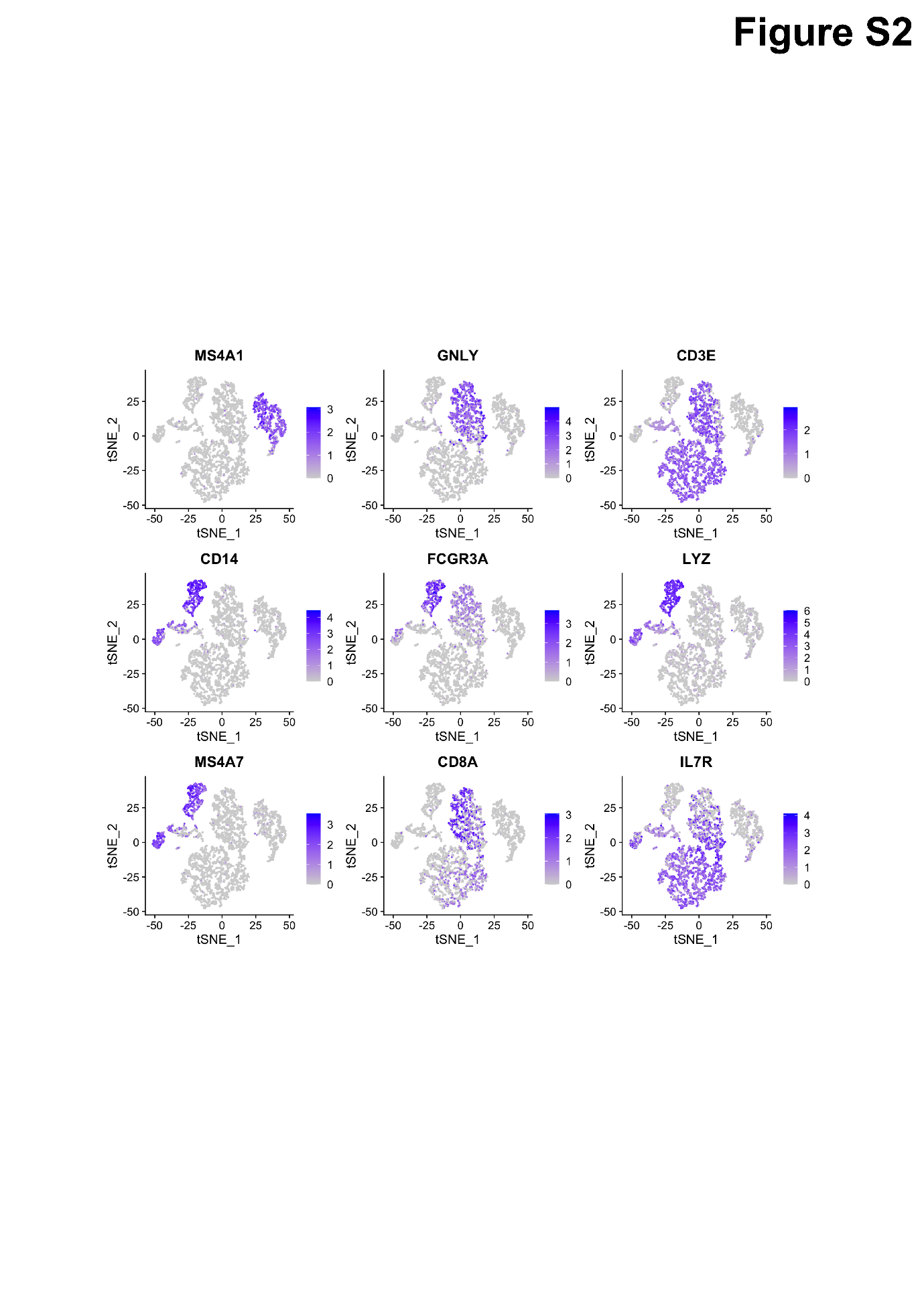
**

**Figure S2. Expression profiles of cell type markers.** CD3E and IL7R (CD4^+^ T cells), CD3E, GNLY, and CD8A (CD8^+^ T and NK cells), MS4A1 (B cells), and CD14, FCGR3A, LYZ, and MS4A7 (Monocytes).

**
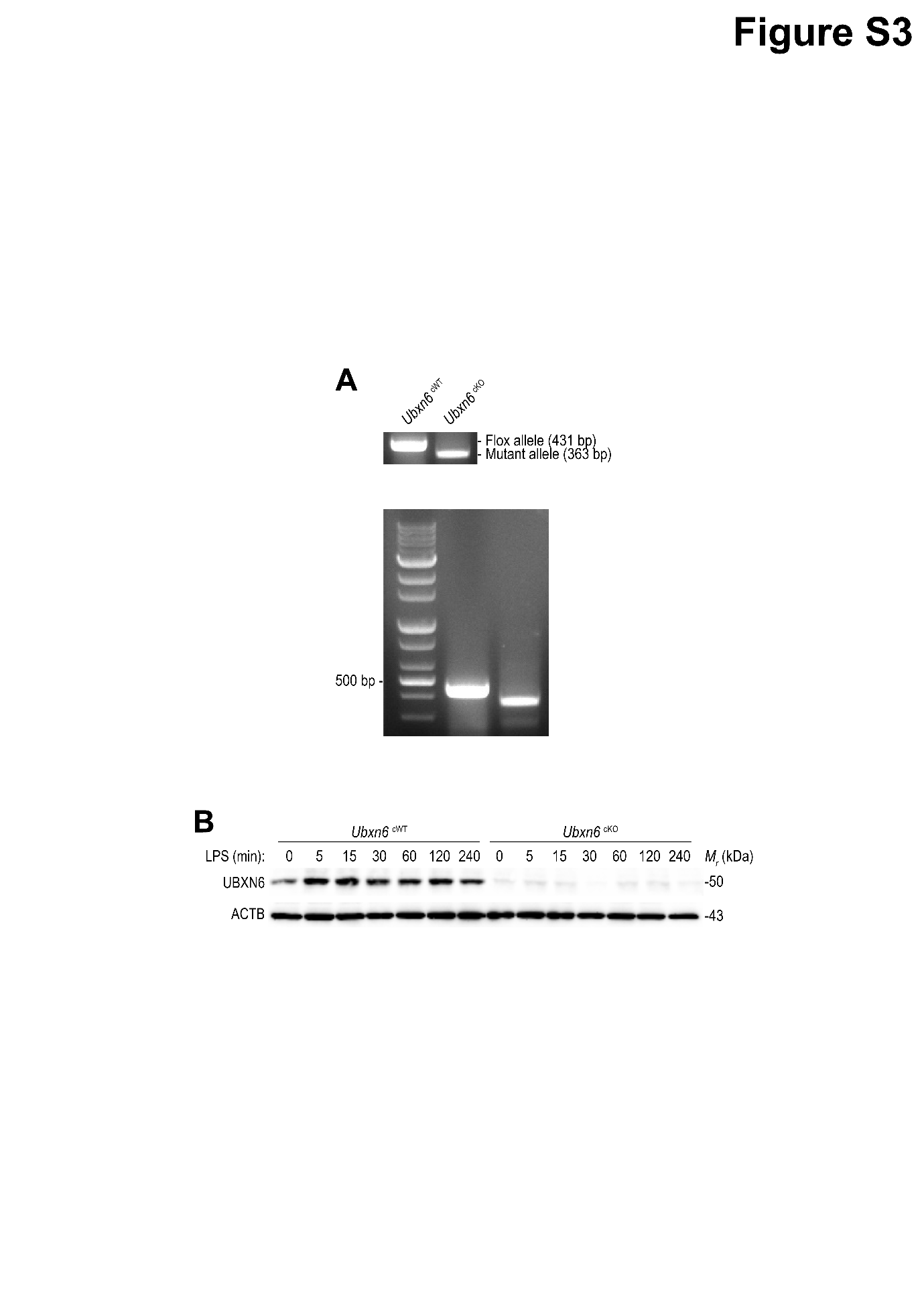
**

**Figure S3. Confirmation of *Ubxn6* knockout in myeloid cells.** **A** Genotype profiles assessed by PCR and electrophoresis. The second lane from *Ubxn6* cWT mice produces only 431 bp amplicon, while the third lane from *Ubxn6* cKO mice produces a 363 bp amplicon. For PCR amplicon size verification, a 1.5 kb DNA ladder is used (first lane). **B** Western blotting of UBXN6 in BMDMs stimulated with LPS (100 ng/mL) for the indicated times. LPS, lipopolysaccharide.

**
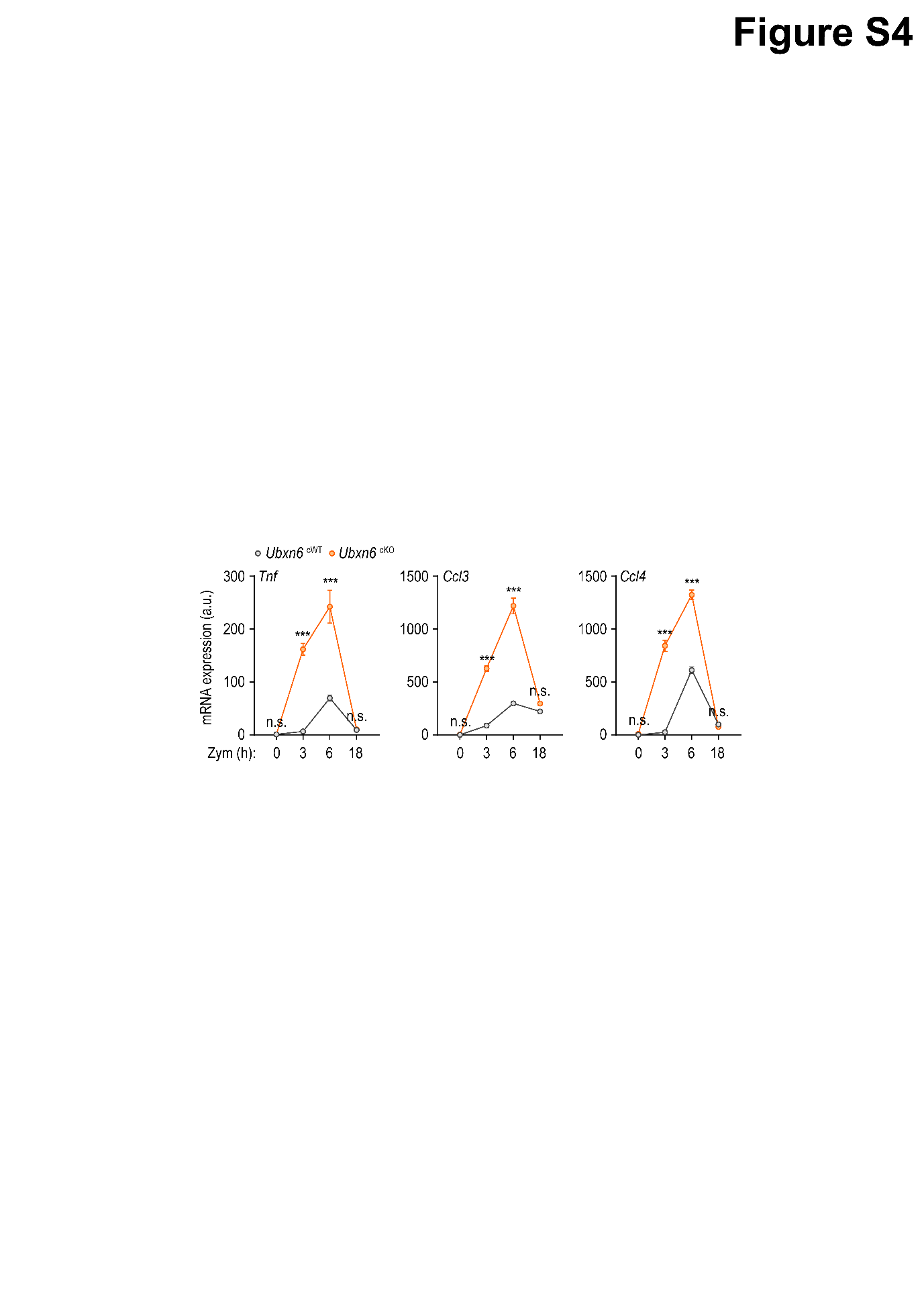
**

**Figure S4. UBXN6 suppresses zymosan-induced induction of proinflammatory cytokines in macrophages.** Relative mRNA expression levels of *Tnf*, *Ccl3*, and *Ccl4* by qRT-PCR analysis in BMDMs treated with zymosan (10 μg/mL) for indicated times. Statistical significance was determined using a one-way ANOVA with Tukey's multiple comparison test. Zym, zymosan; a.u., arbitrary unit; n.s., not significant. Data are presented as means ± SD from at least three independent experiments. ****p* < 0.001.

**
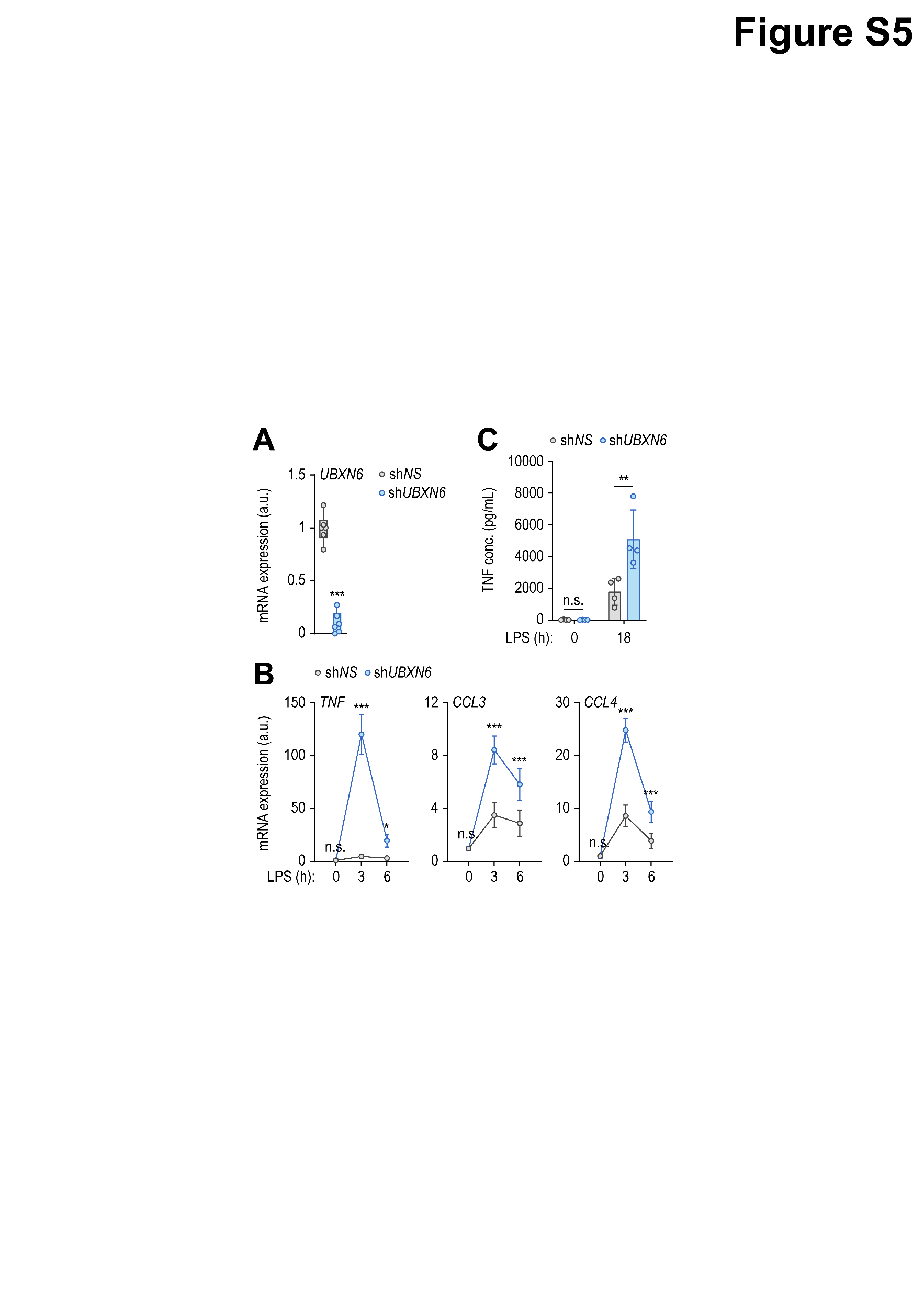
**

**Figure S5. UBXN6 inhibits LPS-induced pro-inflammatory responses in human primary monocytes.** **A** Relative mRNA expression level of *UBXN6* for the knockdown efficiency in human primary monocytes transfected with sh*UBXN6* or sh*NS* for 36 h. **B** The mRNA expression levels of *TNF*, *CCL3*, and *CCL4* after treatment with LPS for the indicated times in human primary monocytes. **C** TNF protein levels after stimulation with or without LPS for 18 h in human primary monocytes. Statistical significance determined using two-tailed Student’s *t* test (**A** and **B**) or one-way ANOVA with Tukey’s multiple comparison test (**C**). LPS, lipopolysaccharide; a.u., arbitrary unit; n.s., not significant. Data are presented as means ± SEM from at least three independent experiments (**A**-**C**). ***p* < 0.01 and ****p* < 0.001.

**
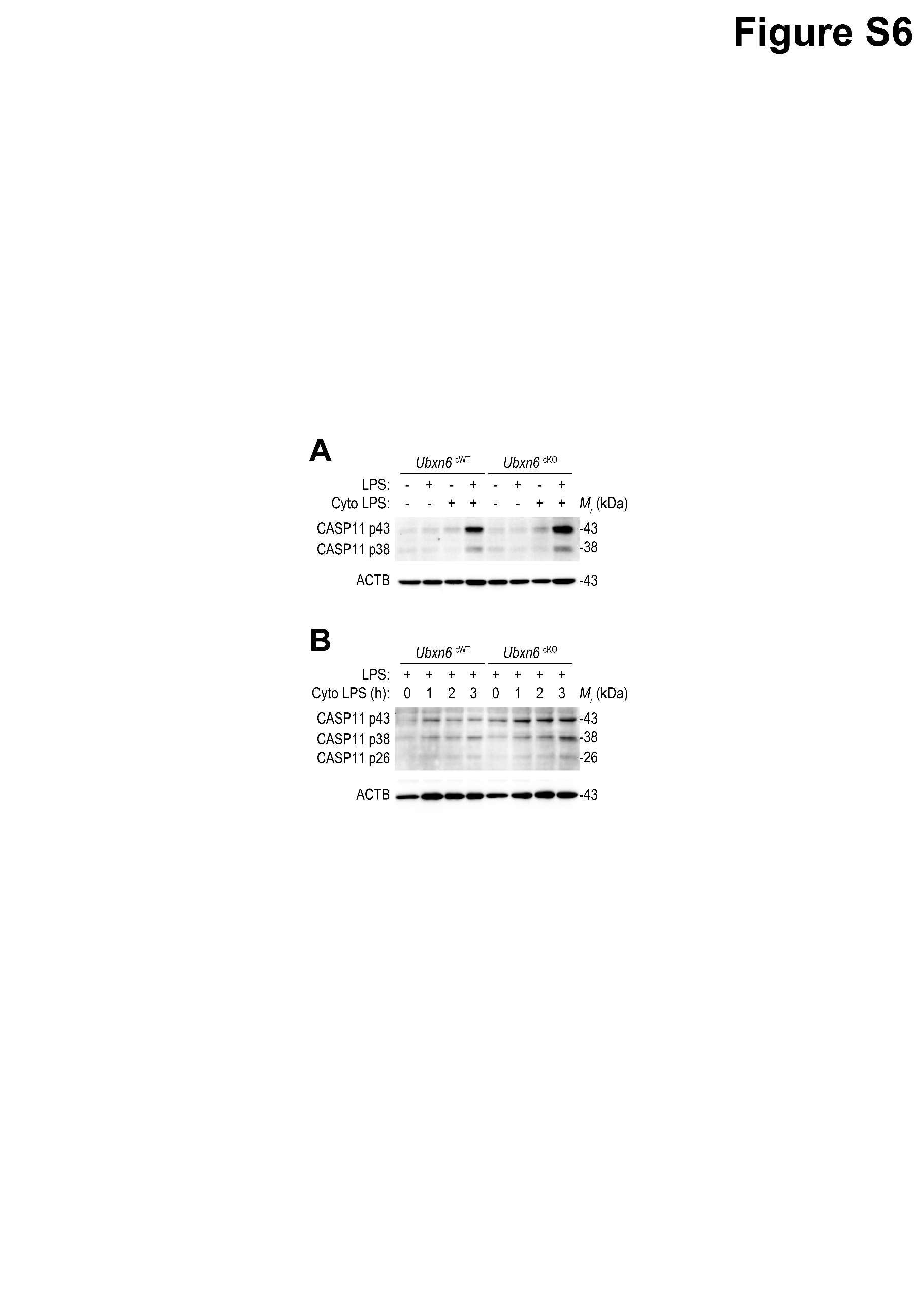
**

**Figure S6. UBXN6 downregulates cytosolic LPS-induced caspase-11 activation in macropahges.** **A** Immunoblots of CASP11 and ACTB (for loading control) in BMDMs. Cells were primed with or without LPS (100 ng/mL) for 4 h and then transfected with or without LPS (2 μg/mL) for 2 h (**A**) or for the indicated times (**B**). ACTB represents loading control. LPS, lipopolysaccharide.

**
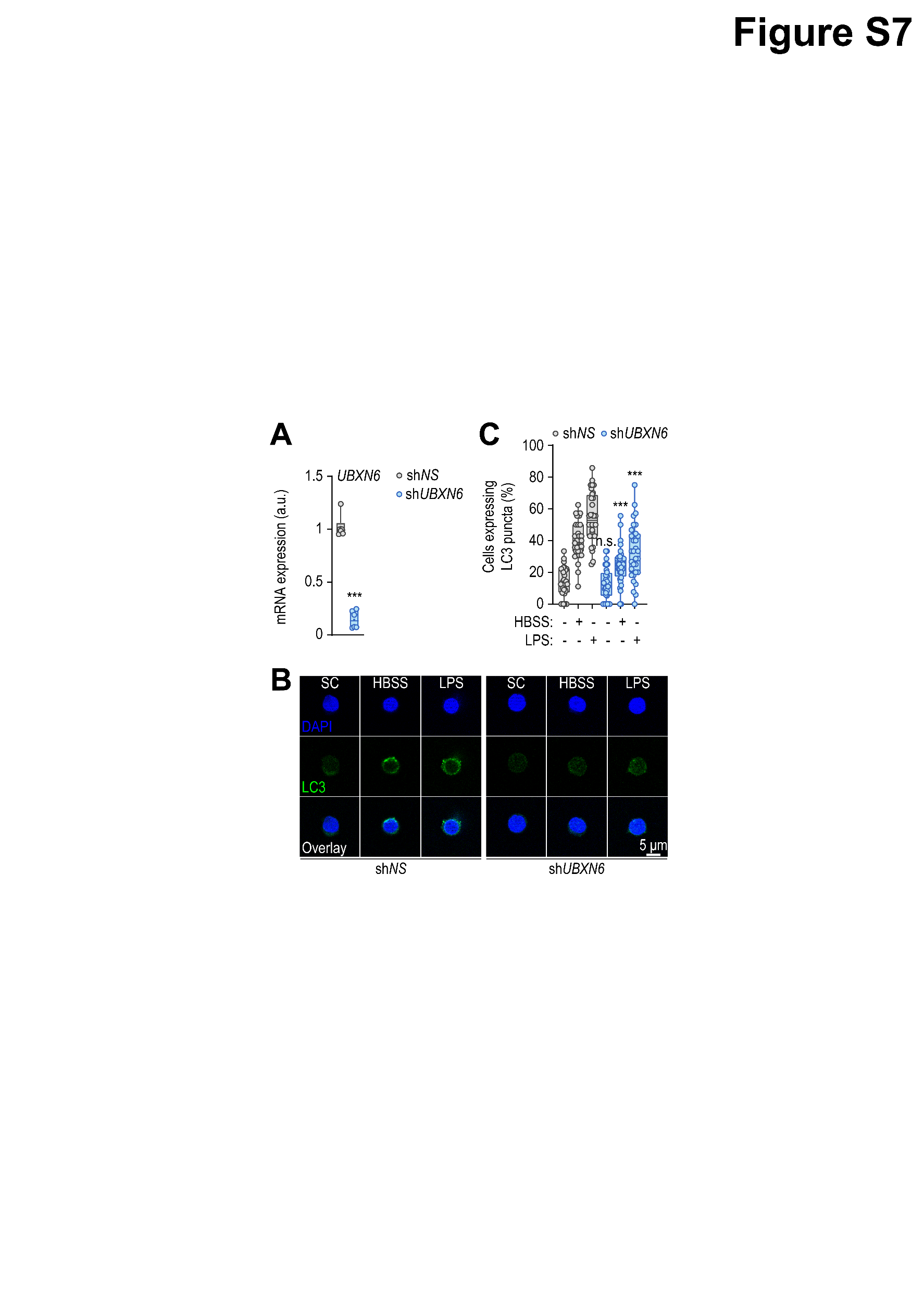
**

**Figure S7. UBXN6 is required for autophagy induction in human primary monocytes in response to LPS stimulation or starvation.** **A** Relative mRNA expression level of *UBXN6* for the knockdown efficiency in human primary monocytes transfected with sh*UBXN6* or sh*NS* for 36 h. **B**, **C** Representative fluorescent microscopy images (**B**) and quantifications (**C**) of LC3 puncta (green) formation after stimulation with LPS (100 ng/mL) for 18 h or starvation with HBSS for 12 h in human primary monocytes. Statistical significance determined using two-tailed Student’s *t* test (**A** and **C**). LPS, lipopolysaccharide; a.u., arbitrary unit; n.s., not significant. Data are presented as means ± SEM from at least three independent experiments (**A** and **C**). ****p* < 0.001.

**
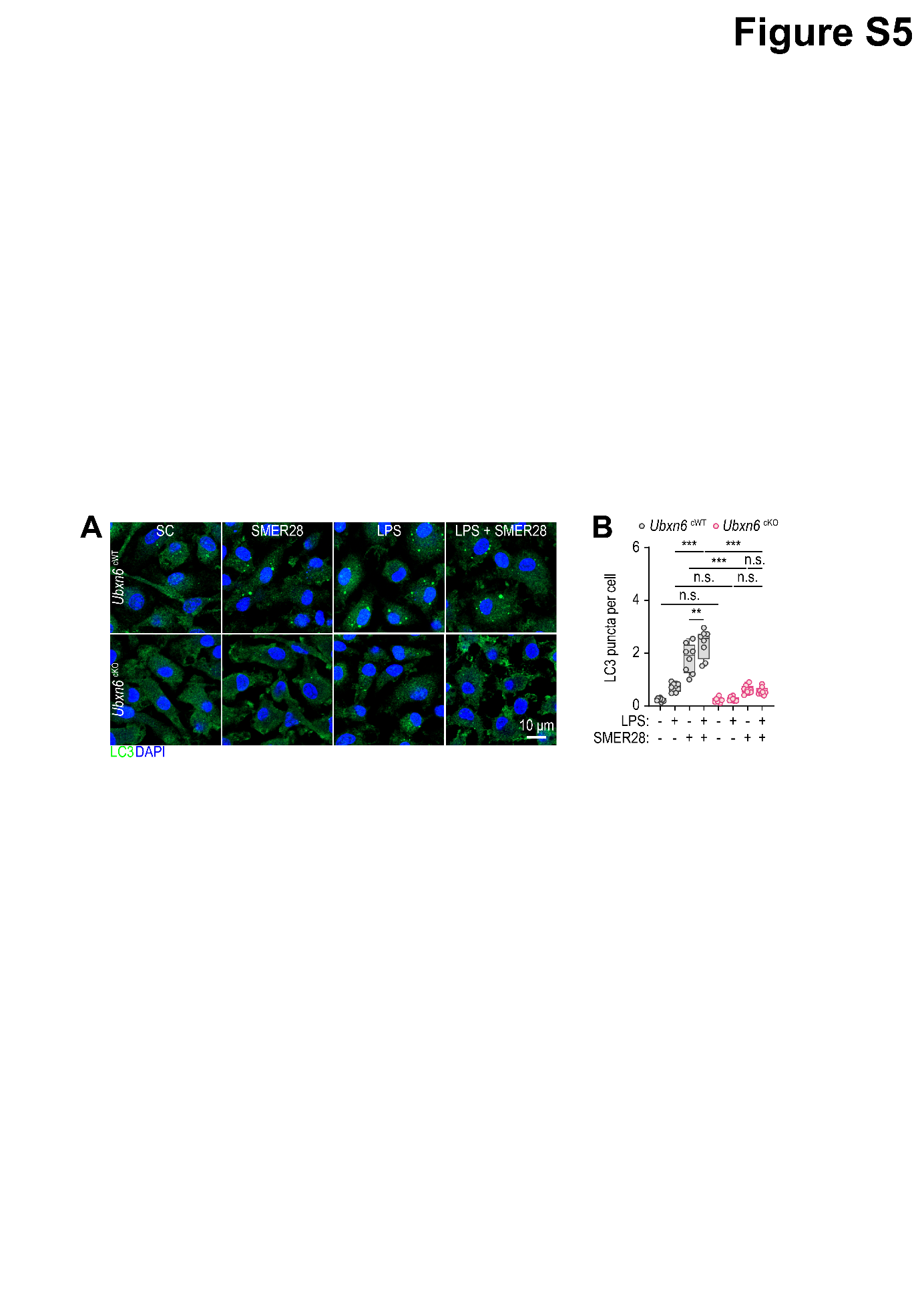
**

**Figure S8. UBXN6 is required to promote VCP/p97-mediated autophagosome formation.** **A**, **B** Representative confocal images (**A**) and quantification (**B**) of LC3 puncta formation in BMDMs. Cells were pre-treated with SMER28 (20 μM) for 1 h before being exposed to LPS (100 ng/mL) for 18 h. One-way ANOVA with Tukey’s multiple comparison test (**B**) was used to determine statistical significance. LPS, lipopolysaccharide; n.s., not significant. Data are presented as means ± SD from at least three independent experiments (**B**). ***p* < 0.01 and ****p* < 0.001.


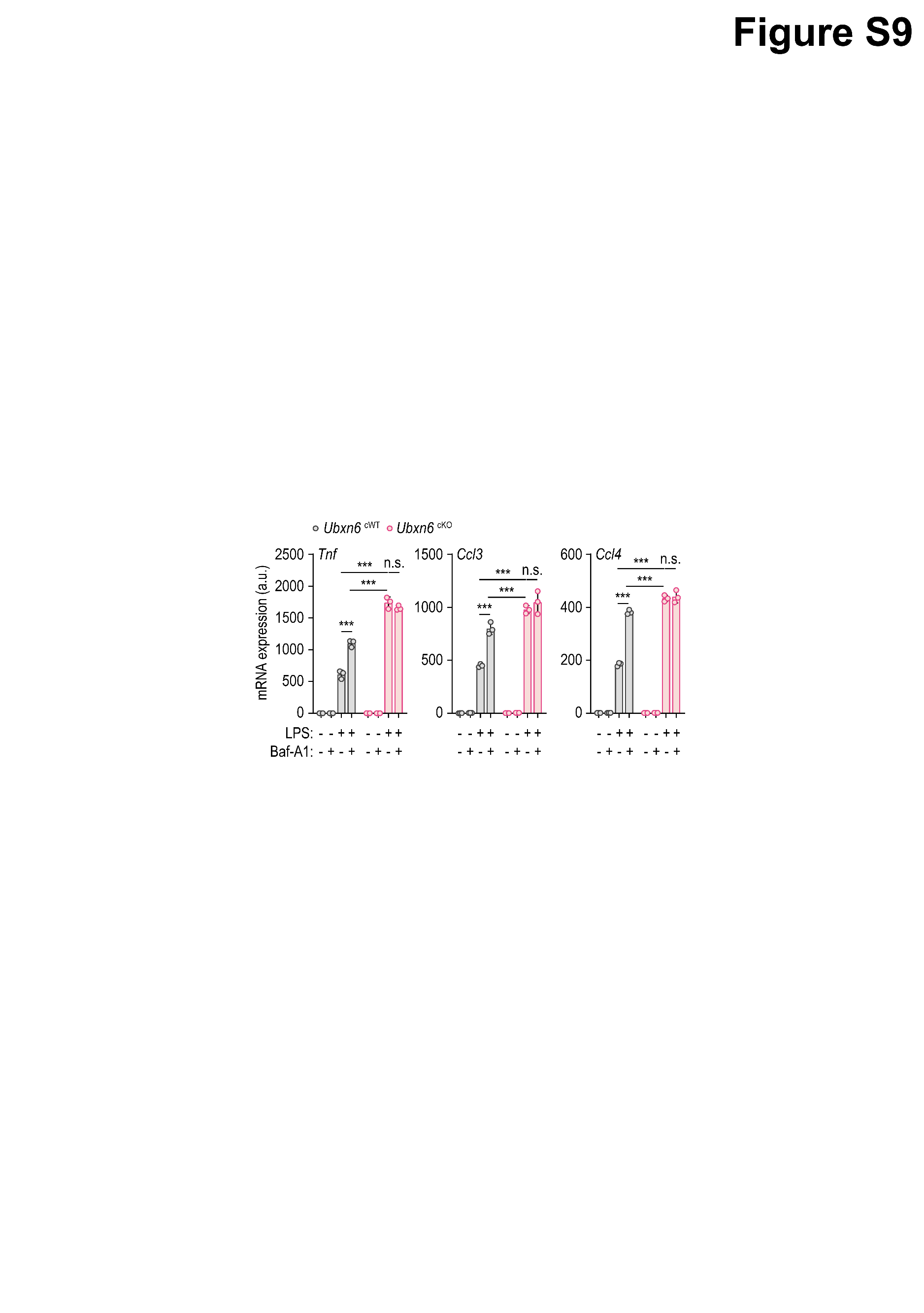


**Figure S9. UBXN6-mediated anti-inflammatory responses are partially dependent on the activation of autophagy.** Relative mRNA expression levels of *Tnf*, *Ccl3*, and *Ccl4* in BMDMs. Cells were pre-treated with or without Baf-A1 (100 nM) for 1 h and then stimulated with or without LPS (100 ng/mL). After 6 h, cells were lysed to conduct qRT-PCR. One-way ANOVA with Tukey’s multiple comparison test was used to determine statistical significance. LPS, lipopolysaccharide; Baf-A1, bafilomycin A1; a.u., arbitrary unit; n.s., not significant. Data are presented as means ± SD from at least three independent experiments. ****p* < 0.001.

**
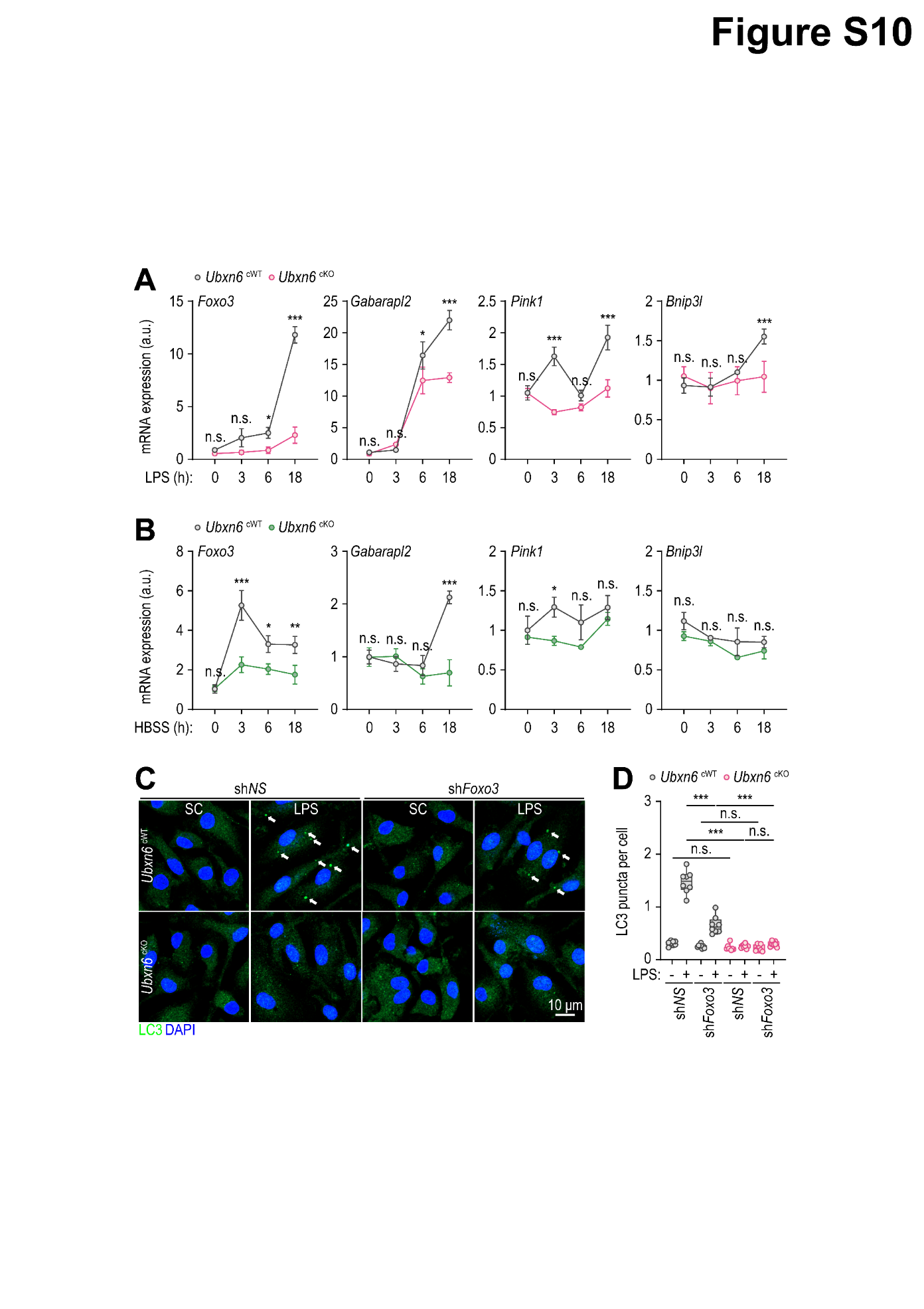
**

**Figure S10. UBXN6-induced autophagy activation is dependent on the FOXO3 signaling pathway in macrophages.** **A**, **B** Relative mRNA expression levels of *Foxo3*, *Gabarapl2*, *Pink1*, and *Bnip3l* in BMDMs stimulated with LPS (100 ng/mL, **A**) or starved with HBSS (**B**) for the indicated times. **C**, **D** Representative images (**C**) and quantification (**D**) of LC3 puncta formation (green, indicated by the white arrows) in BMDMs transfected with sh*Foxo3* or sh*NS* for 36 h and then stimulated with LPS (100 ng/mL) for 18 h. Statistical significance was determined using one-way ANOVA with Tukey's multiple comparison test (**A**, **B**, and **D**). LPS, lipopolysaccharide; a.u., arbitrary unit; n.s., not significant. Data are presented as means ± SD from at least three independent experiments (**A**, **B**, and **D**). **p* < 0.05, ***p* < 0.01, and ****p* < 0.001.

**
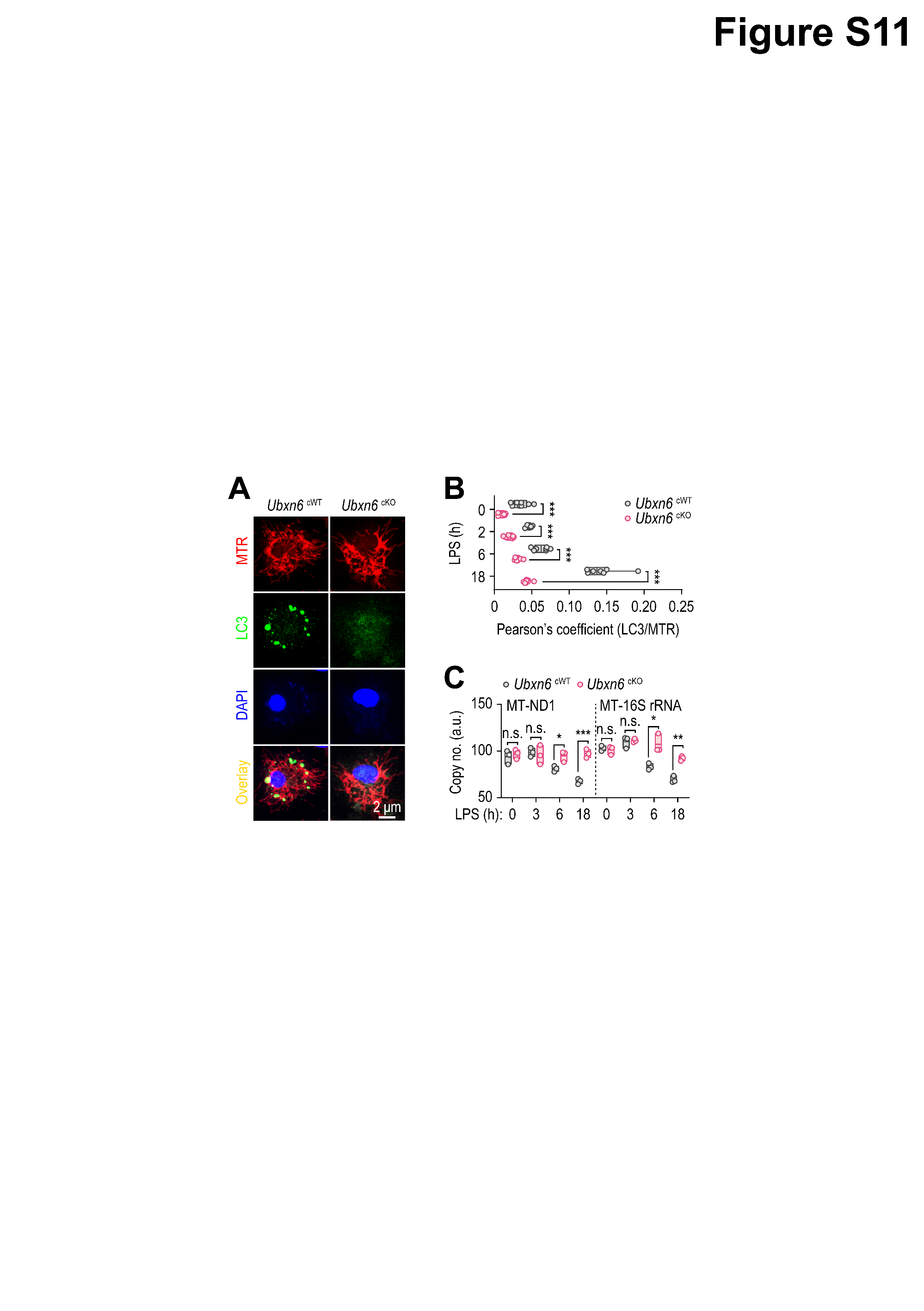
**

**Figure S11. Myeloid UBXN6 enhances LPS-induced mitophagy in macrophages.** **A**, **B** Confocal analysis of mitophagosome activation, MitoTracker Red (Red) staining overlaid with LC3 (green), in BMDMs stimulated with LPS (100 ng/mL) for the indicated times. Representative images are shown in (**A**), and the correlation coefficient between MTR and LC3 is depicted in (**B**). **C** Mitochondrial DNA contents in BMDMs treated with LPS (100 ng/mL) for the indicated times. Statistical significance was determined using one-way ANOVA with Tukey's multiple comparison test (**B**) or two-tailed Student's *t* test (**C**). MTR, MitoTracker Red; LPS, lipopolysaccharide; a.u., arbitrary unit; n.s., not significant. Data are presented as means ± SD from at least three independent experiments (**B** and **C**). **p* < 0.05, ***p* < 0.01, and ****p* < 0.001.

**
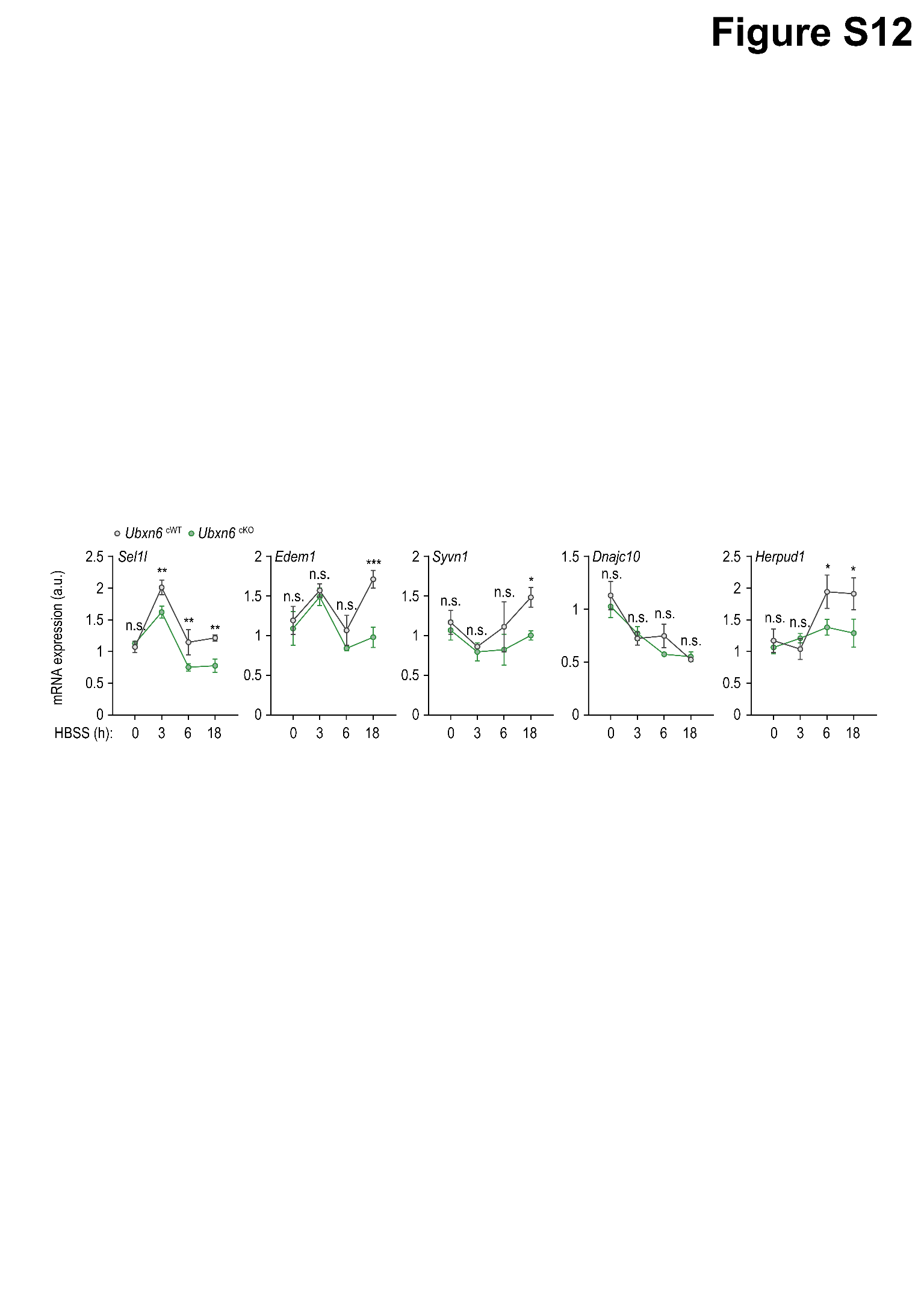
**

**Figure S12. UBXN6 is required for starvation-induced ERAD in macrophages.** The mRNA expression levels of *Sel1l*, *Edem1*, *Syvn1*, *Dnajc10*, and *Herpud1* in BMDMs. The cells were starved with HBSS for the indicated times. Statistical significance was determined using one-way ANOVA with Tukey's multiple comparison test. a.u., arbitrary unit; n.s., not significant. Data are presented as means ± SD from at least three independent experiments. **p* < 0.05, ***p* < 0.01, and ****p* < 0.001.

**
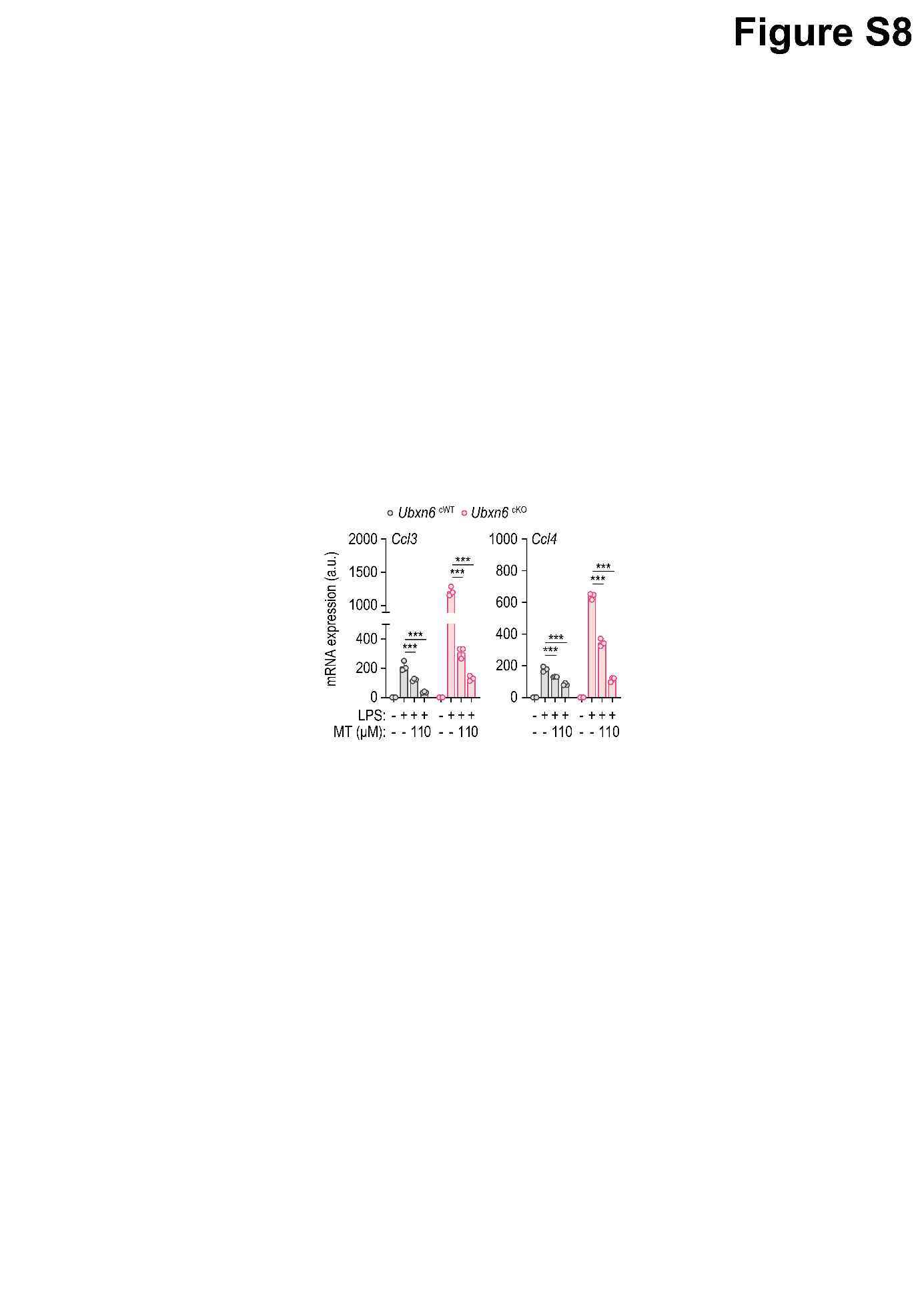
**

**Figure S13. UBXN6-mediated reduction of proinflammatory cytokines in LPS-treated macrophages partially depends on mitochondrial ROS generation.** The relative mRNA expression levels of *Ccl3* and *Ccl4* in BMDMs pre-treated with MitoTEMPO (1 or 10 μM) followed by stimulation with LPS (100 ng/mL) for 6 h. One-way ANOVA with Tukey’s multiple comparison test was used to determine statistical significance. LPS, lipopolysaccharide; a.u., arbitrary unit; MT, MitoTEMPO. Data are present as means ± SD from at least three independent experiments. ****p* < 0.001.


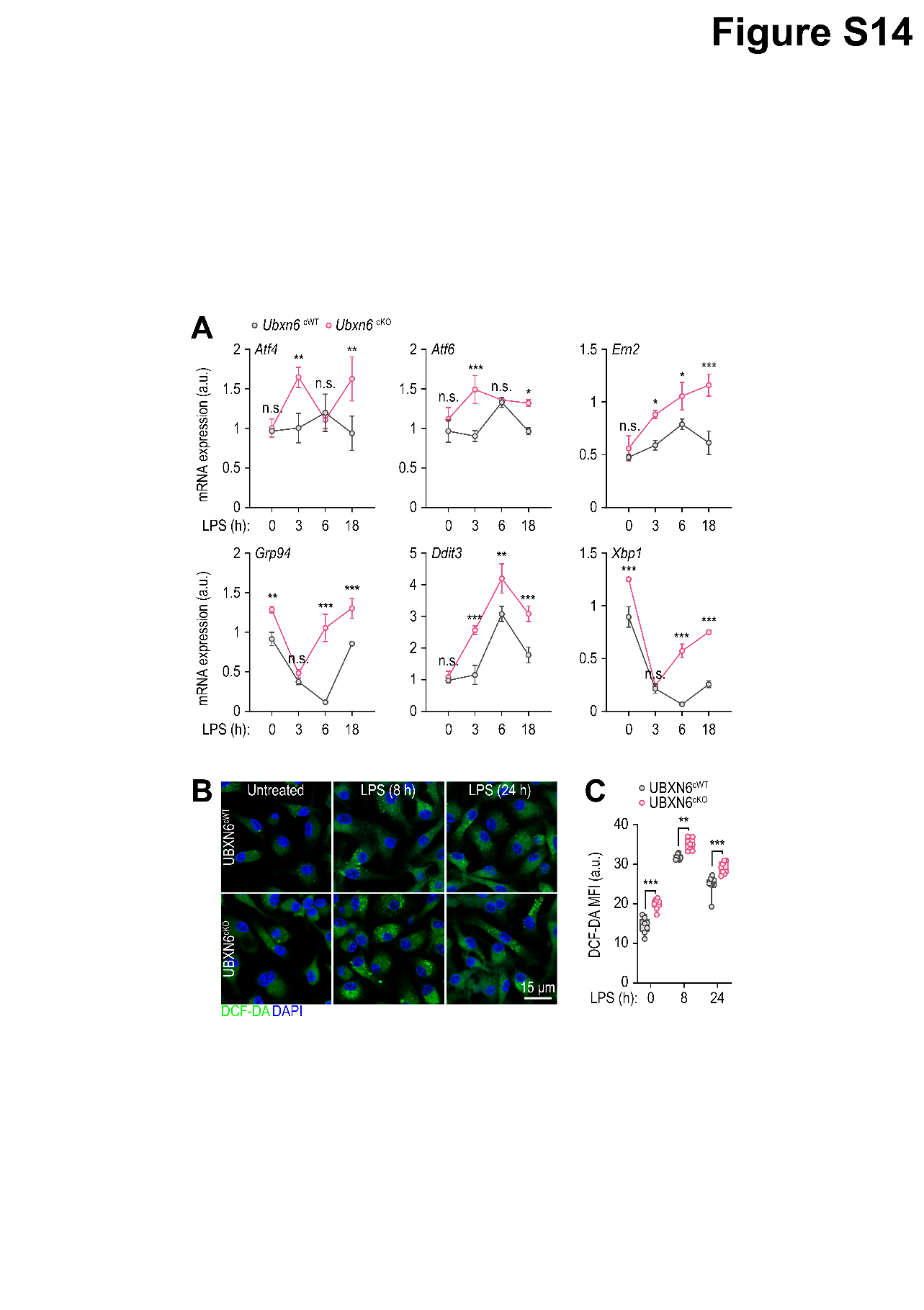


**Figure S14. UBXN6 reduces LPS-induced ER stress-related gene transcription and cellular ROS generation in macrophages.** **A** Relative mRNA expression levels of *Atf4*, *Atf6*, *Ern2*, *Grp94*, *Ddit3*, and *Xbp1* in BMDMs. **B**, **C** Representative immunostaining images (**B**) and quantifications (**C**) of DCF-DA (green, indicating cellular ROS). Cells were treated with LPS (100 ng/mL) for the indicated times and further lysed to conduct qRT-PCR or stained with DCF-DA and DAPI (blue, for nuclei) for confocal analysis. Statistical significance determined using one-way ANOVA with Tukey’s multiple comparison test (**A**) or two-tailed Student’s *t* test (**C**). LPS, lipopolysaccharide; a.u., arbitrary unit; MFI, mean fluorescence intensity; n.s., not significant. Data are presented as means ± SD from at least three independent experiments (**A** and **C**). **p* < 0.05, ***p* < 0.01 and ****p* < 0.001.


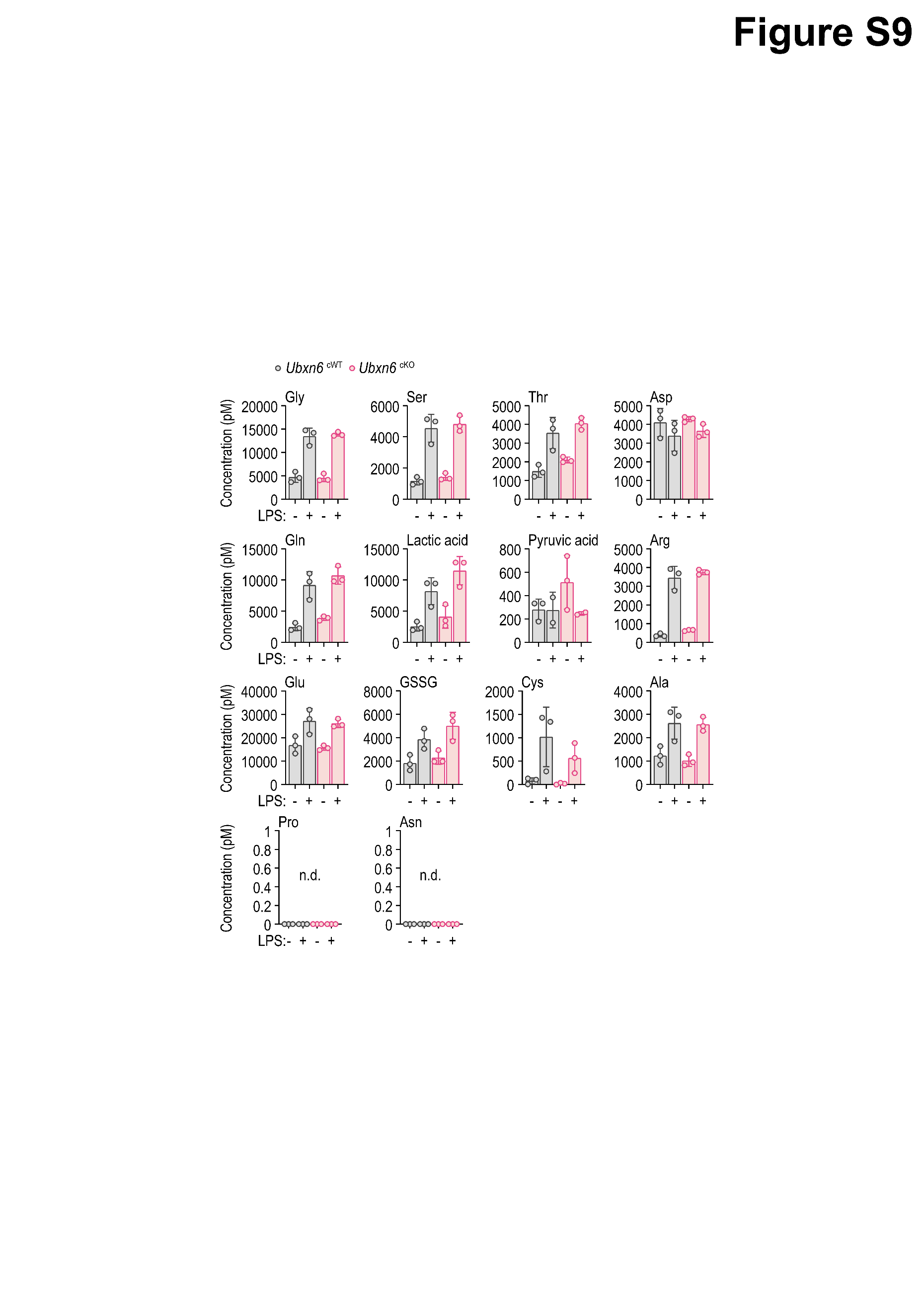


**Figure S15. Myeloid UBXN6 has little effect on the levels of non-essential amino acids, lactic acid, pyruvate acid, and GSSG.** Concentrations of eleven non-essential amino acids, lactic acid, pyruvate acid, and glutathione disulfide in BMDMs stimulated with LPS (100 ng/mL) for 18 h. LPS, lipopolysaccharide; GSSG, glutathione disulfide; n.d., not detected. Data are presented as means ± SD from at least three independent experiments.

**
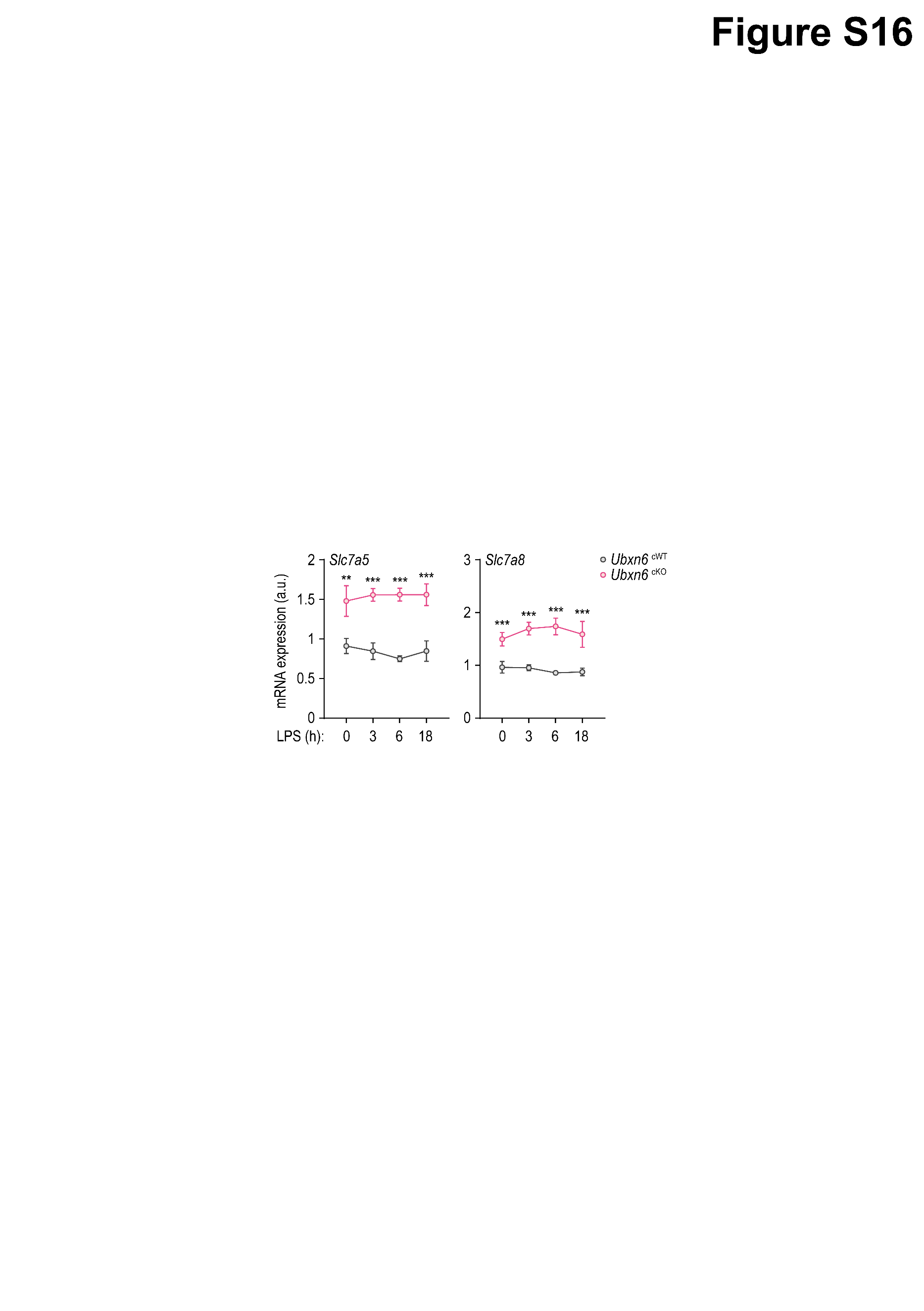
**

**Figure S16. UBXN6 deficiency enhances transcriptional regulation of LATs in macrophages.** The levels of *Slc7a5* and *Slc7a8* mRNA expression in BMDMs. Cells were treated with LPS (100 ng/mL) for the indicated times, and then subjected to conduct qRT-PCR. Statistical significance determined using one-way ANOVA with Tukey’s multiple comparison test. LPS, lipopolysaccharide; a.u., arbitrary unit. Data are presented as means ± SD from at least three independent experiments. ***p* < 0.01 and ****p* < 0.001.

**
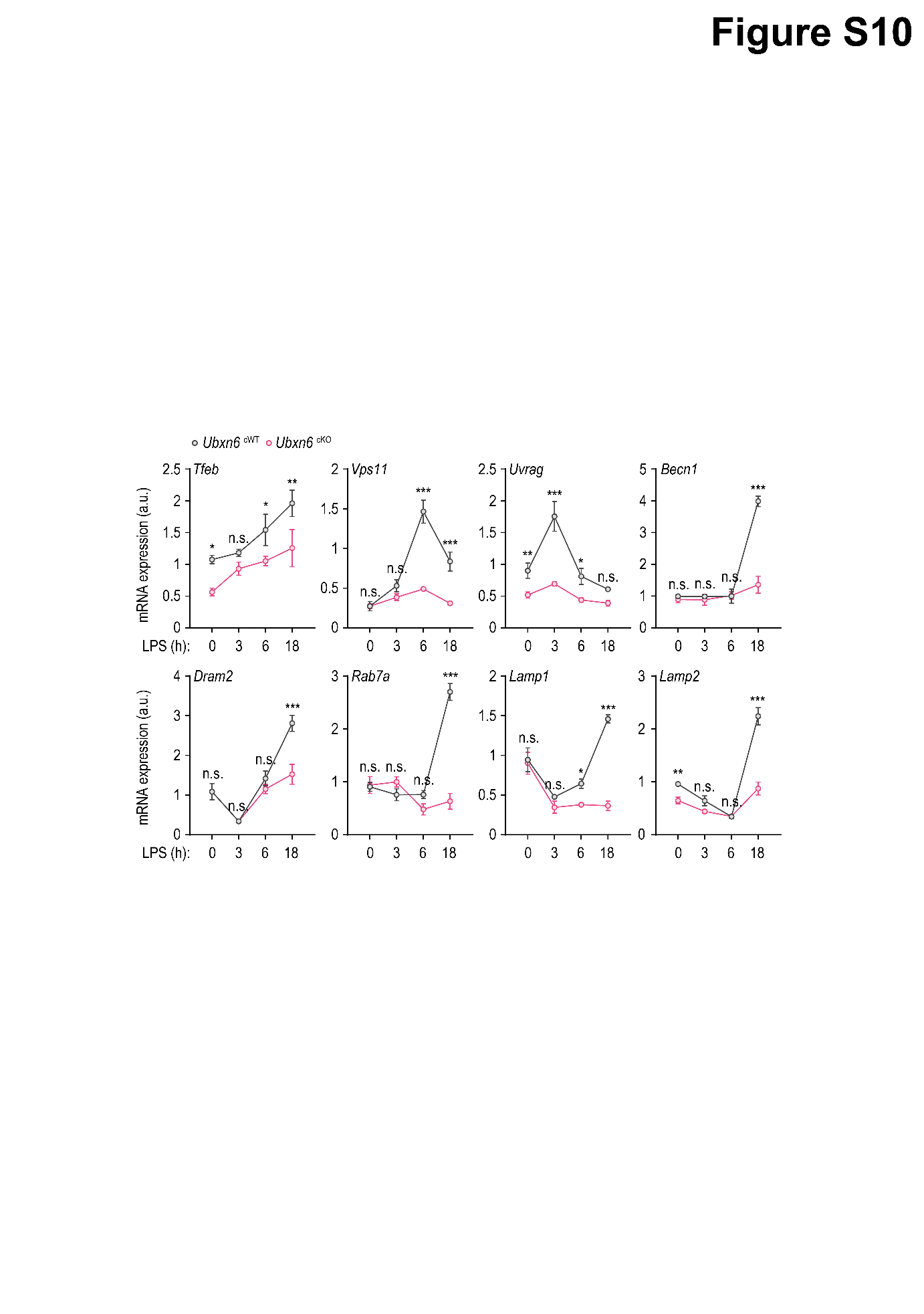
**

**Figure S17. UBXN6 enhances LPS-induced lysosomal biogenesis through TFEB transcriptional activation.** Relative mRNA expression levels of *Tfeb*, *Vps11*, *Uvrag*, *Becn1*, *Dram2*, *Rab7a*, *Lamp1*, and *Lamp2* in BMDMs stimulated with LPS (100 ng/mL) for the indicated times. One-way ANOVA with Tukey’s multiple comparison test was used to determine statistical significance. LPS, lipopolysaccharide; a.u., arbitrary unit; n.s., not significant. Data are presented as means ± SD from at least three independent experiments. **p* < 0.05, ***p* < 0.01 and ****p* < 0.001.

**
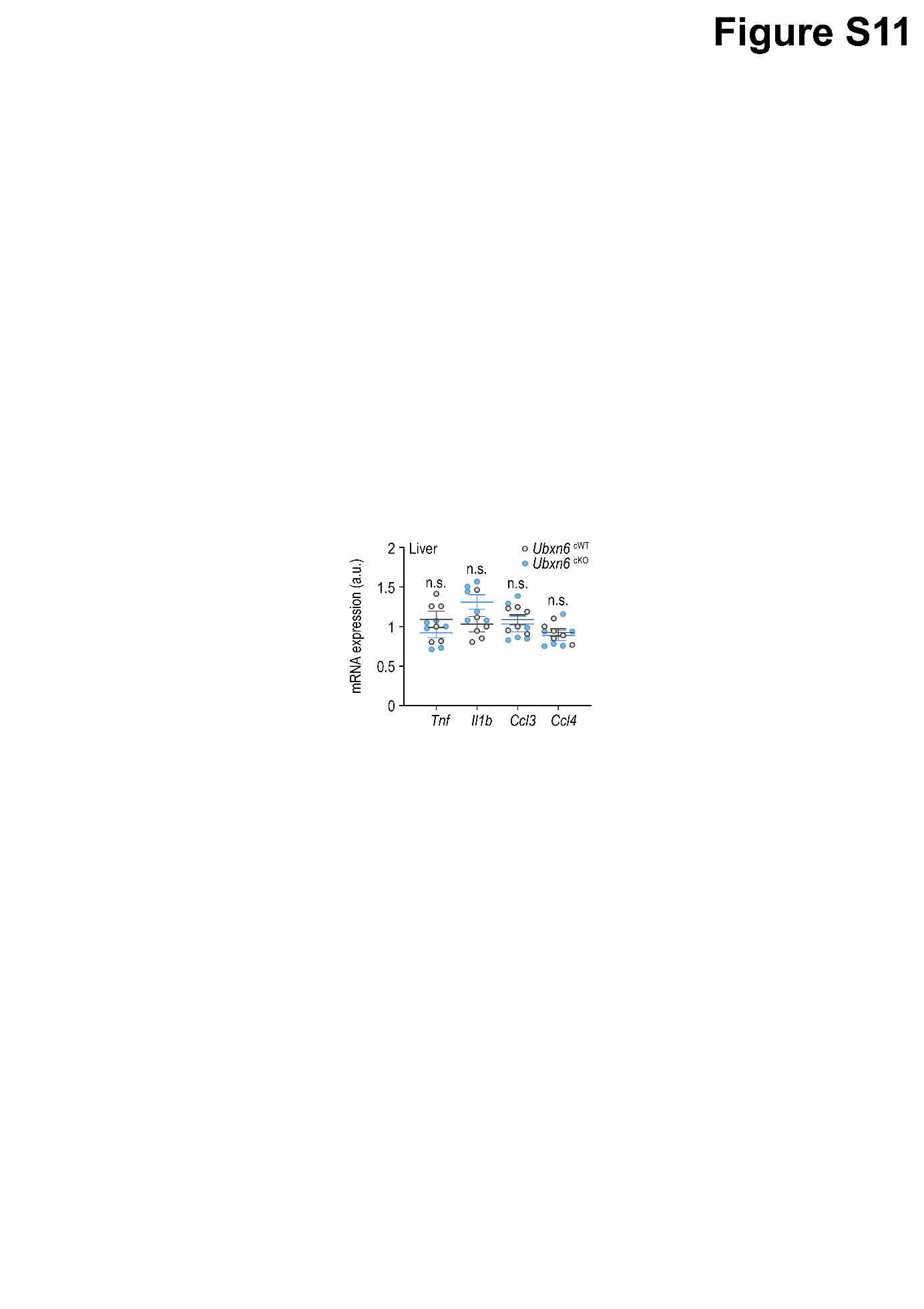
**

**Figure S18. Myeloid cell-derived UBXN6 does not regulate LPS-induced inflammatory responses in the liver.** Relative mRNA levels of *Tnf*, *Il1b*, *Ccl3*, and *Ccl4* in the livers of *Ubxn6* cWT and cKO mice injected with LPS (14 mg/kg) for 6 h. Two-tailed Student’s *t* test was used to determine statistical significance. a.u., arbitrary unit; n.s., not significant. Data are presented as means ± SEM from 3-6 biological repeats.

**
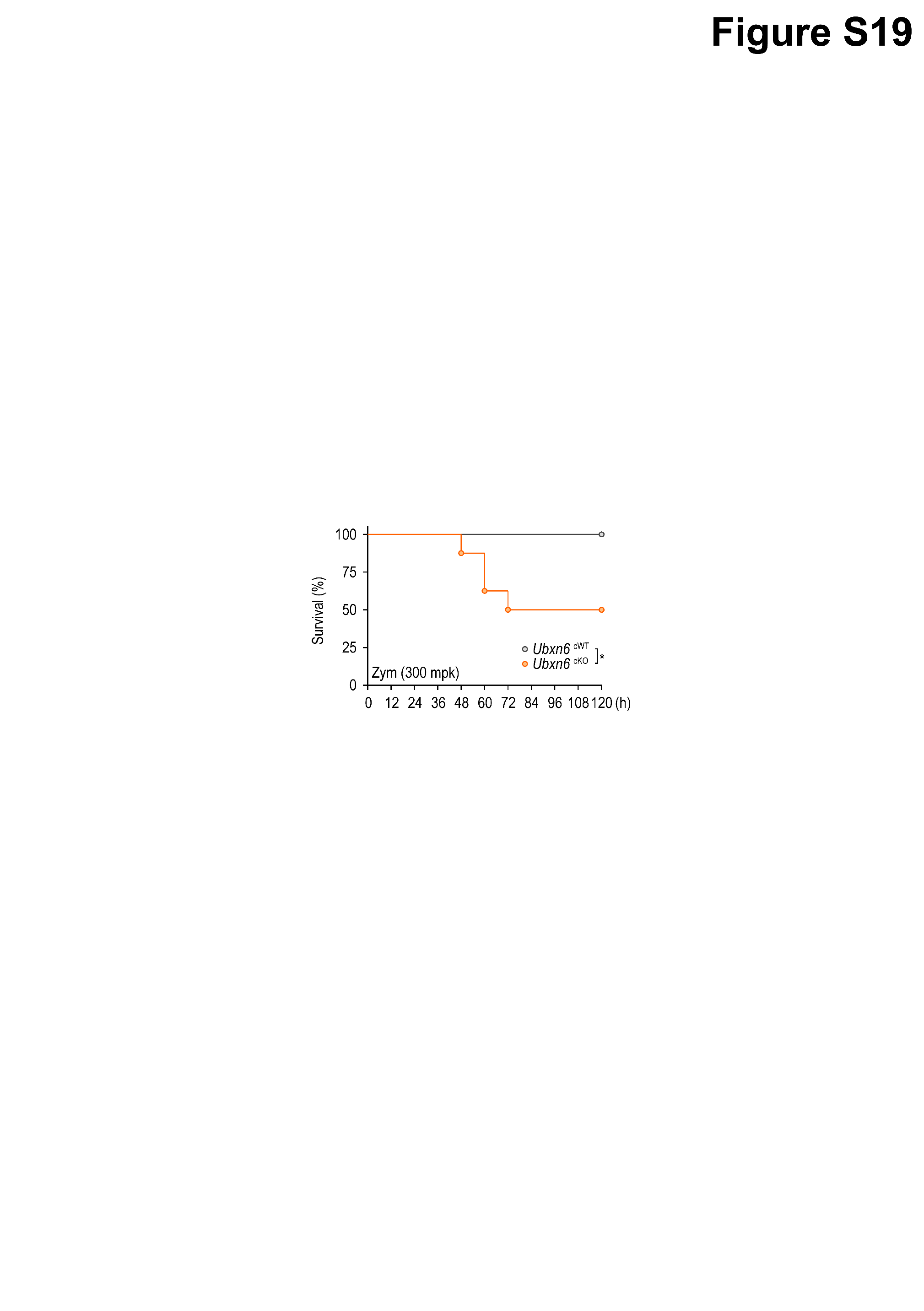
**

**Figure S19. UBXN6 alleviates zymosan-induced mortality *in vivo*.** Survival of *Ubxn6* cWT and cKO mice assessed for 120 h after administration of zymosan (300 mg/kg, n = 8). Statistical significance was determined using the log-rank (Mantel-Cox) test. Zym, zymosan; mpk, milligram per kilogram. **p* < 0.05.

**
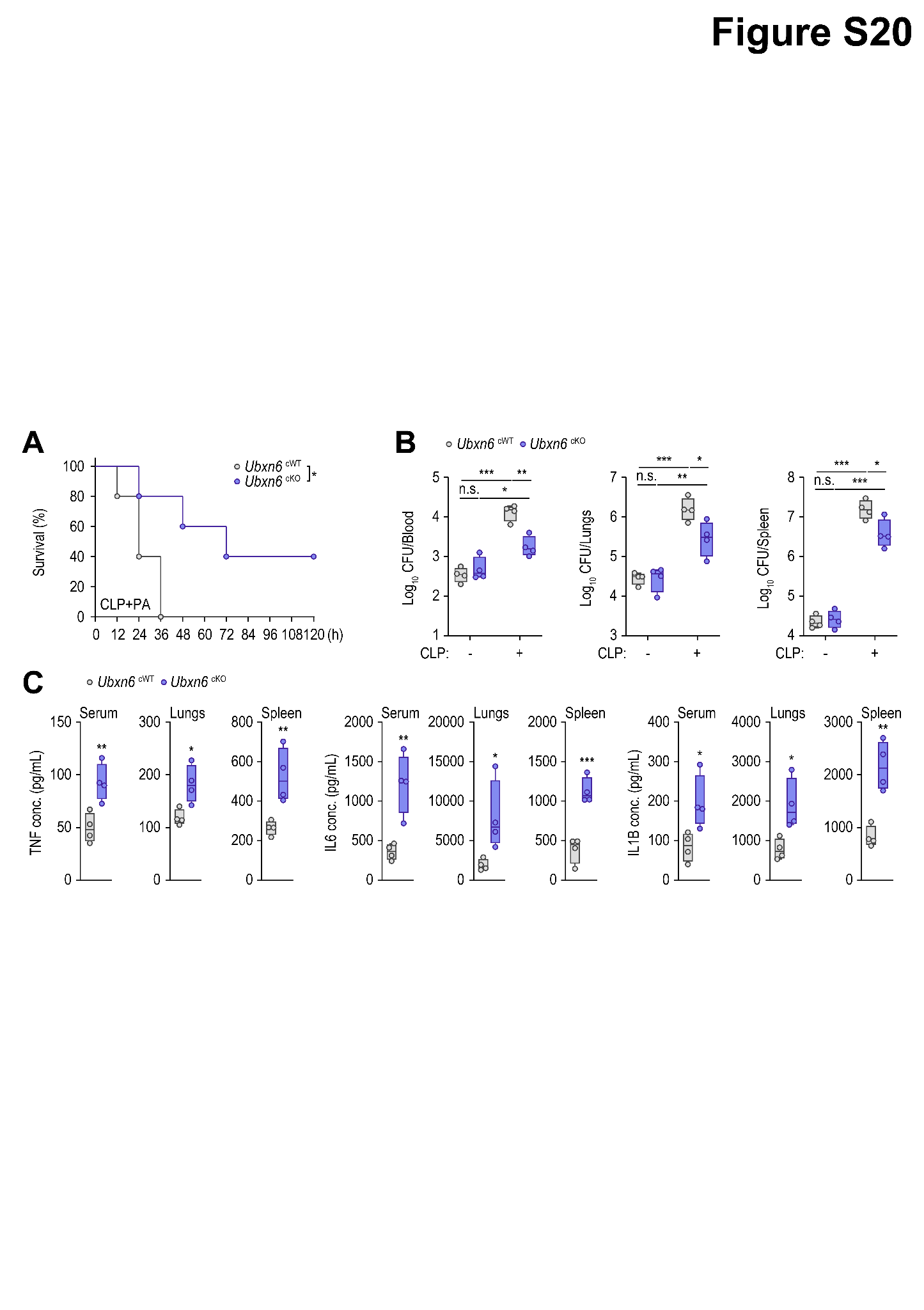
**

**Figure S20. Myeloid UBXN6 is crucial for overcoming immunosuppressive sepsis in mice.** **A** The survival rate of CLP-operated *Ubxn6* cWT and cKO mice followed by PAO1 (3 × 10^6^ CFU/head) intravenous infection (n = 5) which was assessed for 120 h. **B**, **C** Bacterial load (**B**) and protein levels of TNF, IL6, and IL1B (**C**) in the blood, lungs, and spleen of sham- or CLP-operated *Ubxn6* cWT and cKO mice at 4 h after PAO1 infection. Statistical significance determined using the log-rank (Mantel-Cox) test (**A**), one-way ANOVA with Tukey’s multiple comparison test (**B**), or two-tailed Student’s *t* test (**C**). CLP, cecal ligation and puncture; PA, *Pseudomonas aeruginosa*; CFU, colony forming unit; conc., concentration; n.s., not significant. Data are presented as means ± SEM from four biological repeats (**B** and **C**). **p* < 0.05, ***p* < 0.01 and ****p* < 0.001.
