# Supplementary material for: Ubiquitin regulatory X (UBX) domain-containing protein 6 is essential for autophagy induction and inflammation control in macrophages
Source: Cell Mol Immunol. 2024 Oct 23;21(12):1441–58. doi: 10.1038/s41423-024-01222-1 (PMC11606977; doi:10.1038/s41423-024-01222-1)
Supplement: Supplementary file 6 — Uncropped Western blots [file 41423_2024_1222_MOESM6_ESM.pptx]

## Slide 1
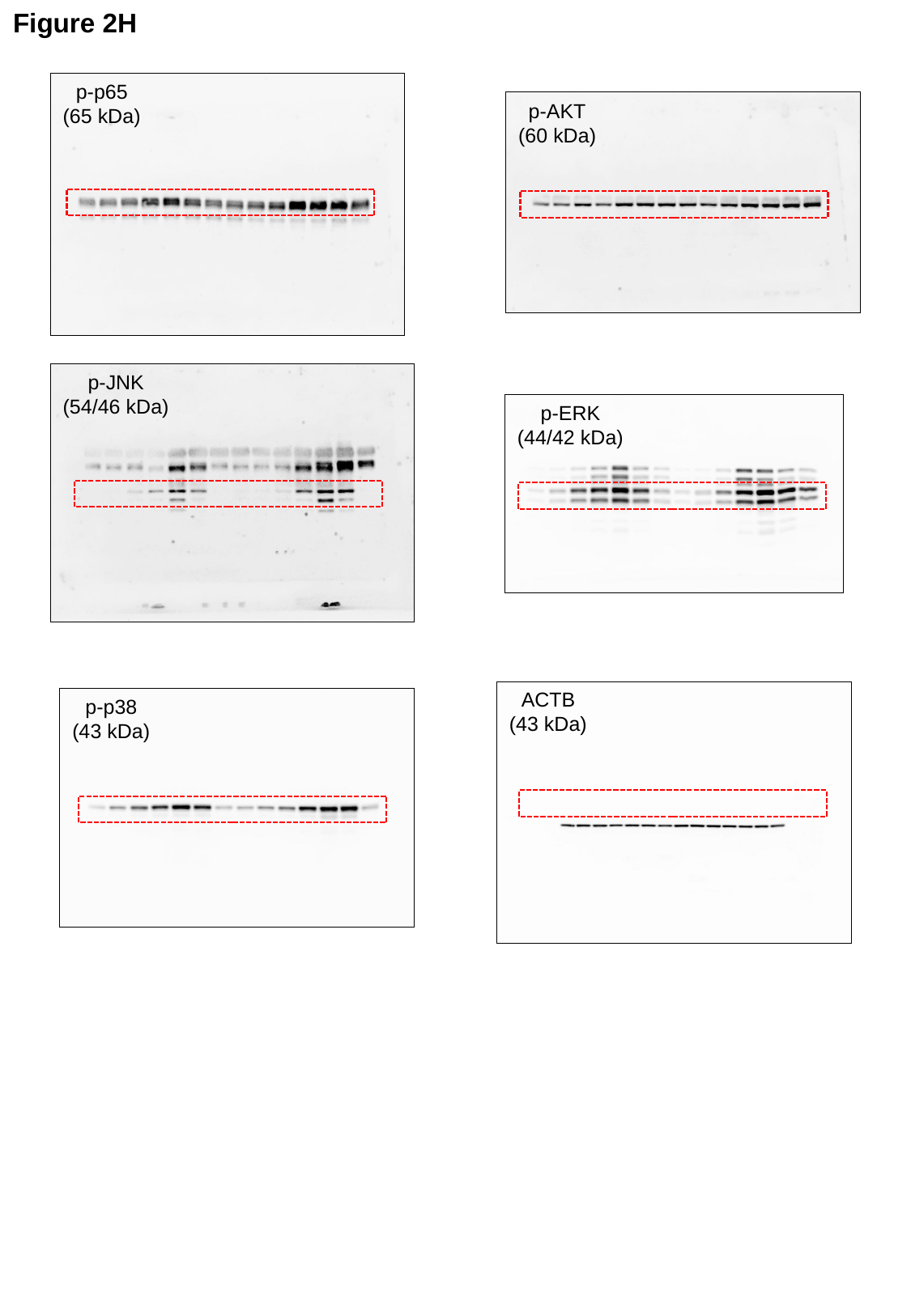

Figure 2H
p-p65
(65 kDa)
p-AKT
(60 kDa)
p-JNK
(54/46 kDa)
p-ERK
(44/42 kDa)
ACTB
(43 kDa)
p-p38
(43 kDa)

## Slide 2
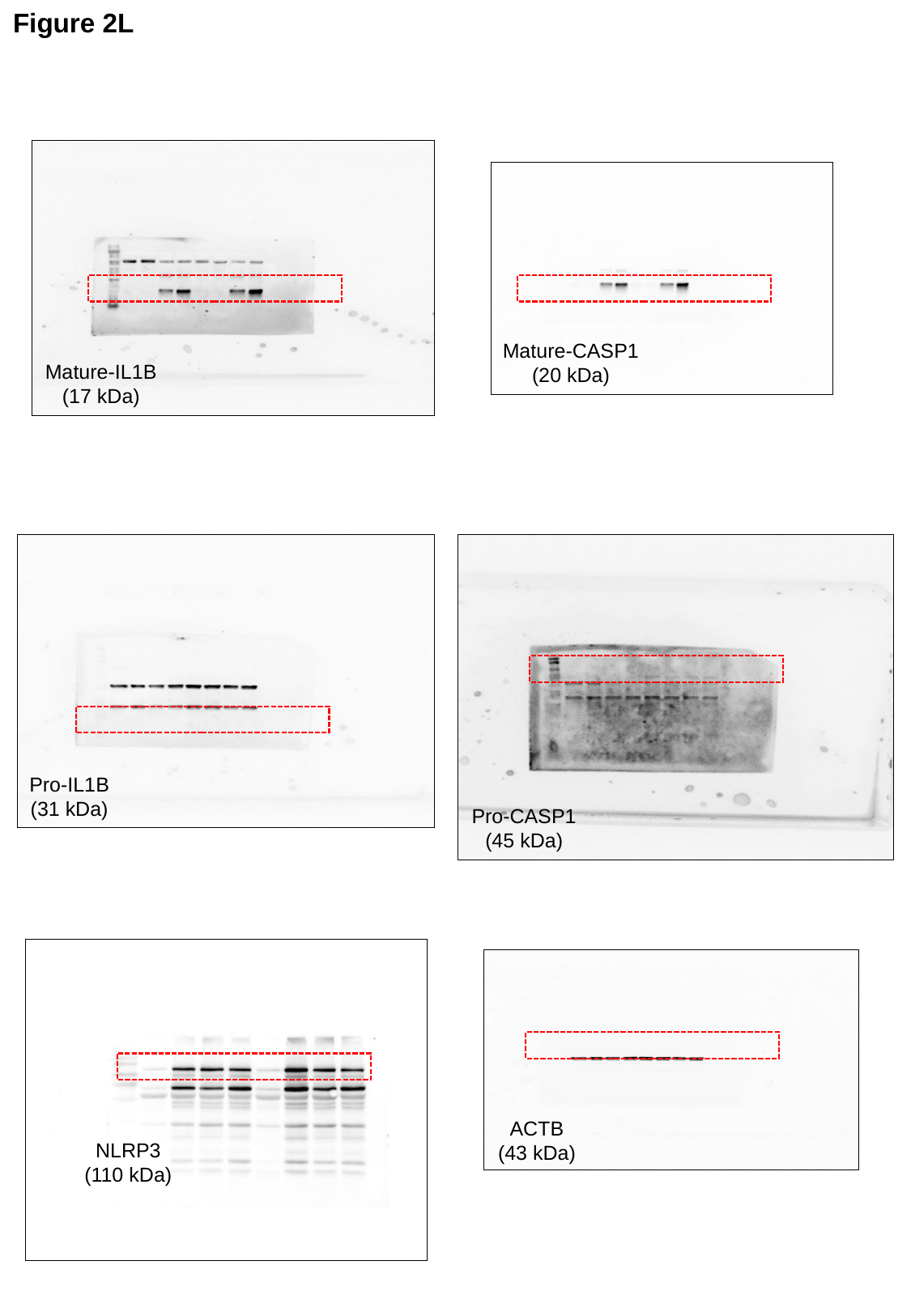

Figure 2L
Mature-CASP1
(20 kDa)
Mature-IL1B
(17 kDa)
Pro-IL1B
(31 kDa)
Pro-CASP1
(45 kDa)
ACTB
(43 kDa)
NLRP3
(110 kDa)

## Slide 3
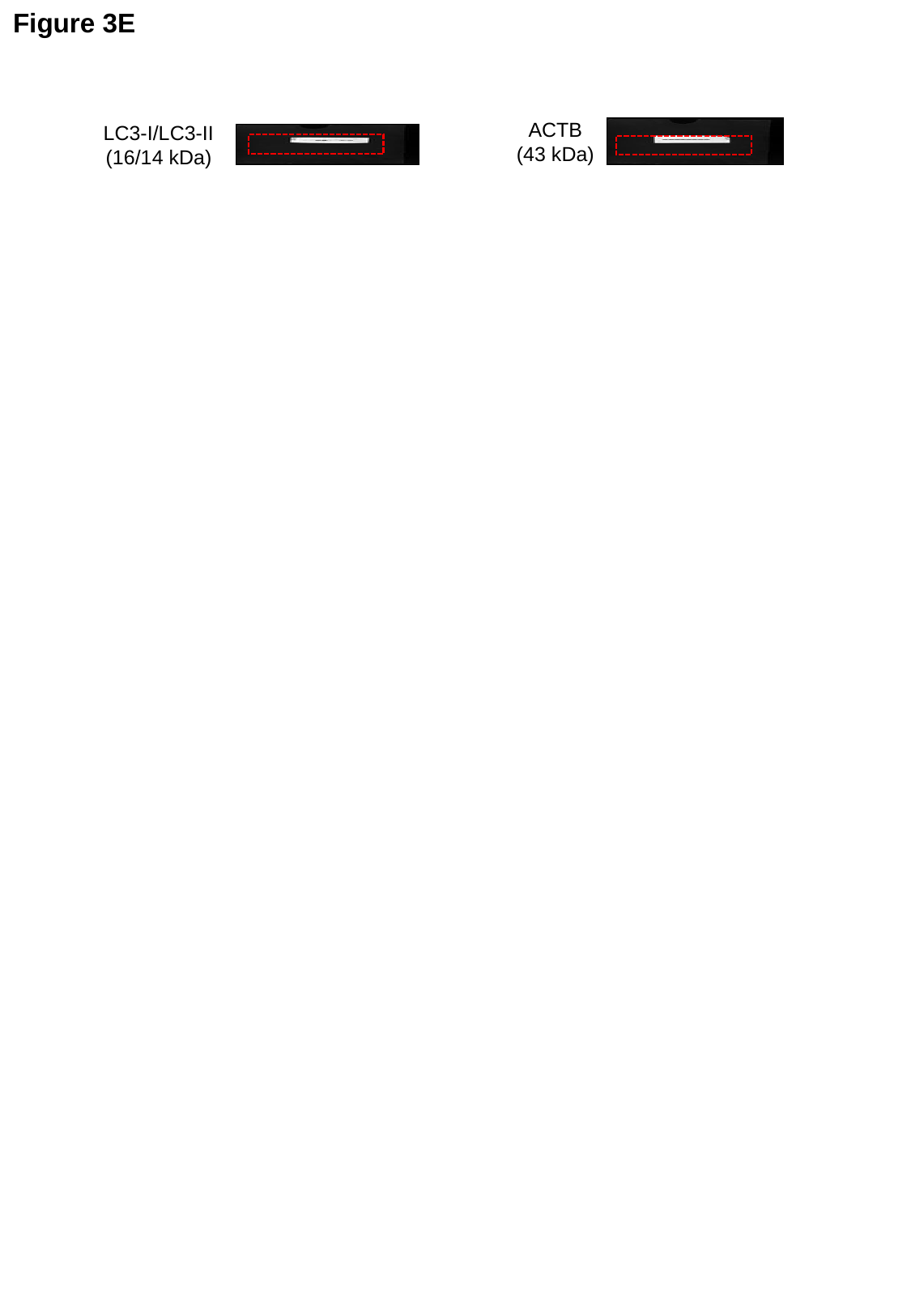

Figure 3E
ACTB
(43 kDa)
LC3-I/LC3-II
(16/14 kDa)

## Slide 4
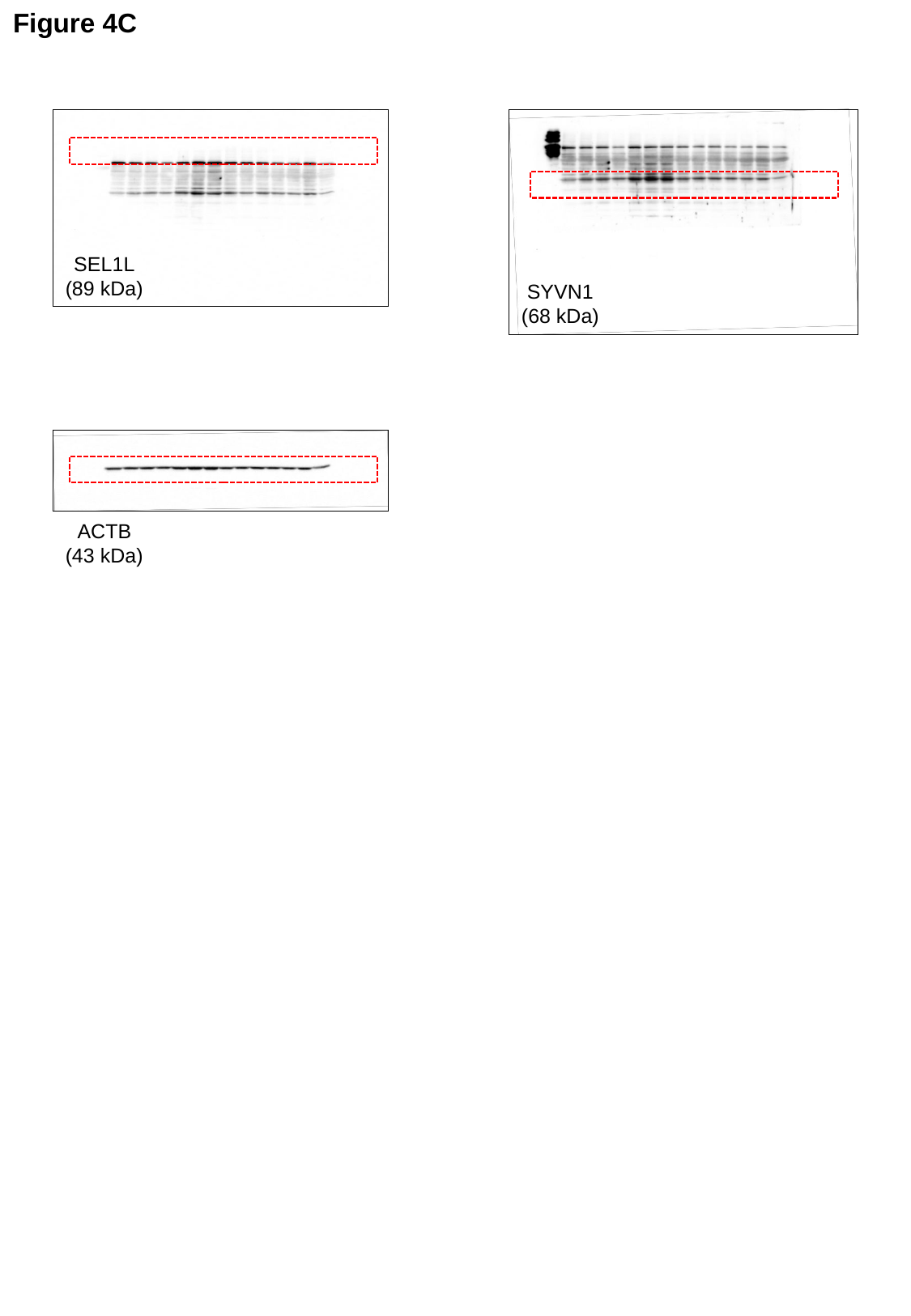

Figure 4C
SEL1L
(89 kDa)
SYVN1
(68 kDa)
ACTB
(43 kDa)

## Slide 5
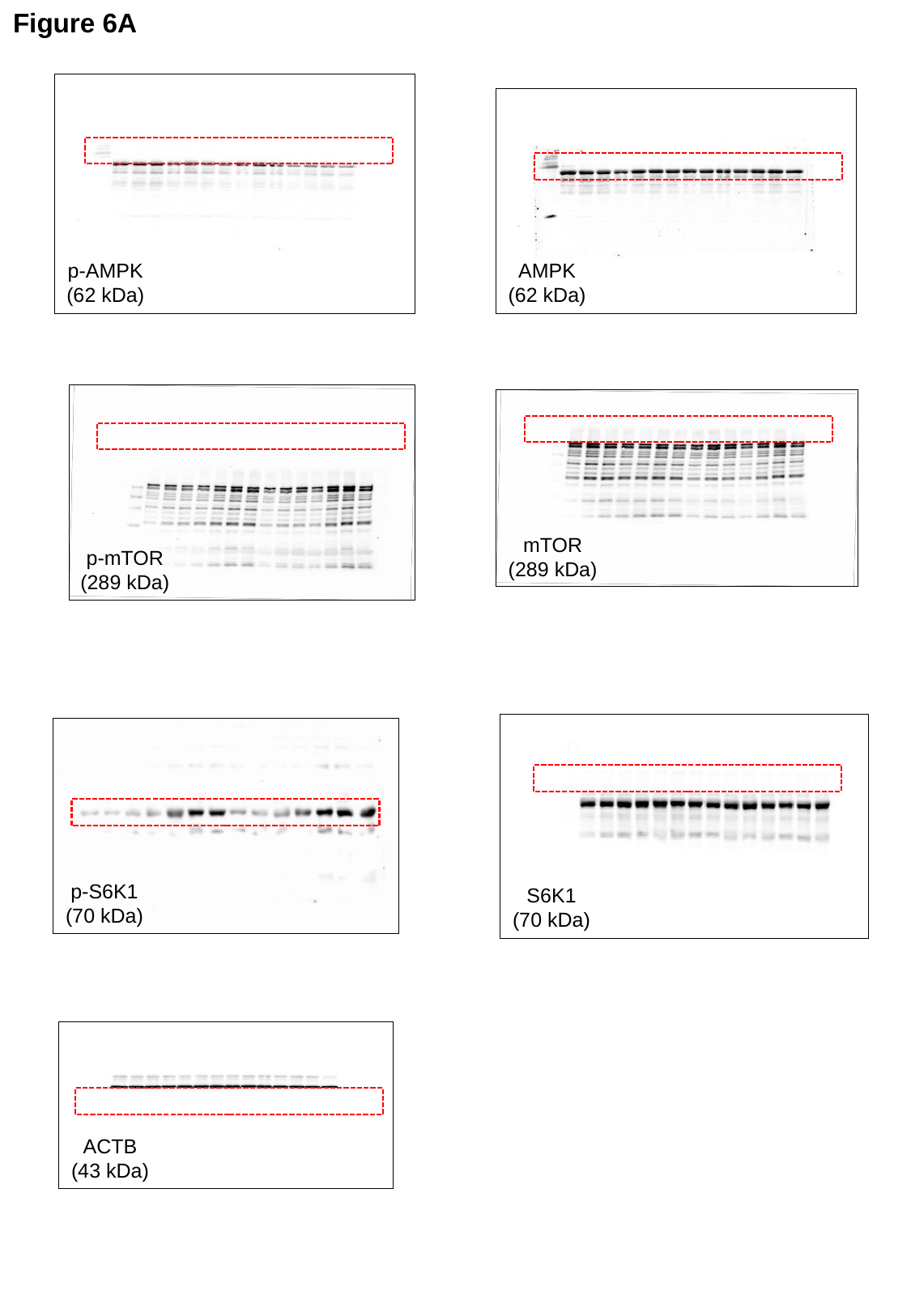

Figure 6A
p-AMPK
(62 kDa)
AMPK
(62 kDa)
mTOR
(289 kDa)
p-mTOR
(289 kDa)
p-S6K1
(70 kDa)
S6K1
(70 kDa)
ACTB
(43 kDa)

## Slide 6
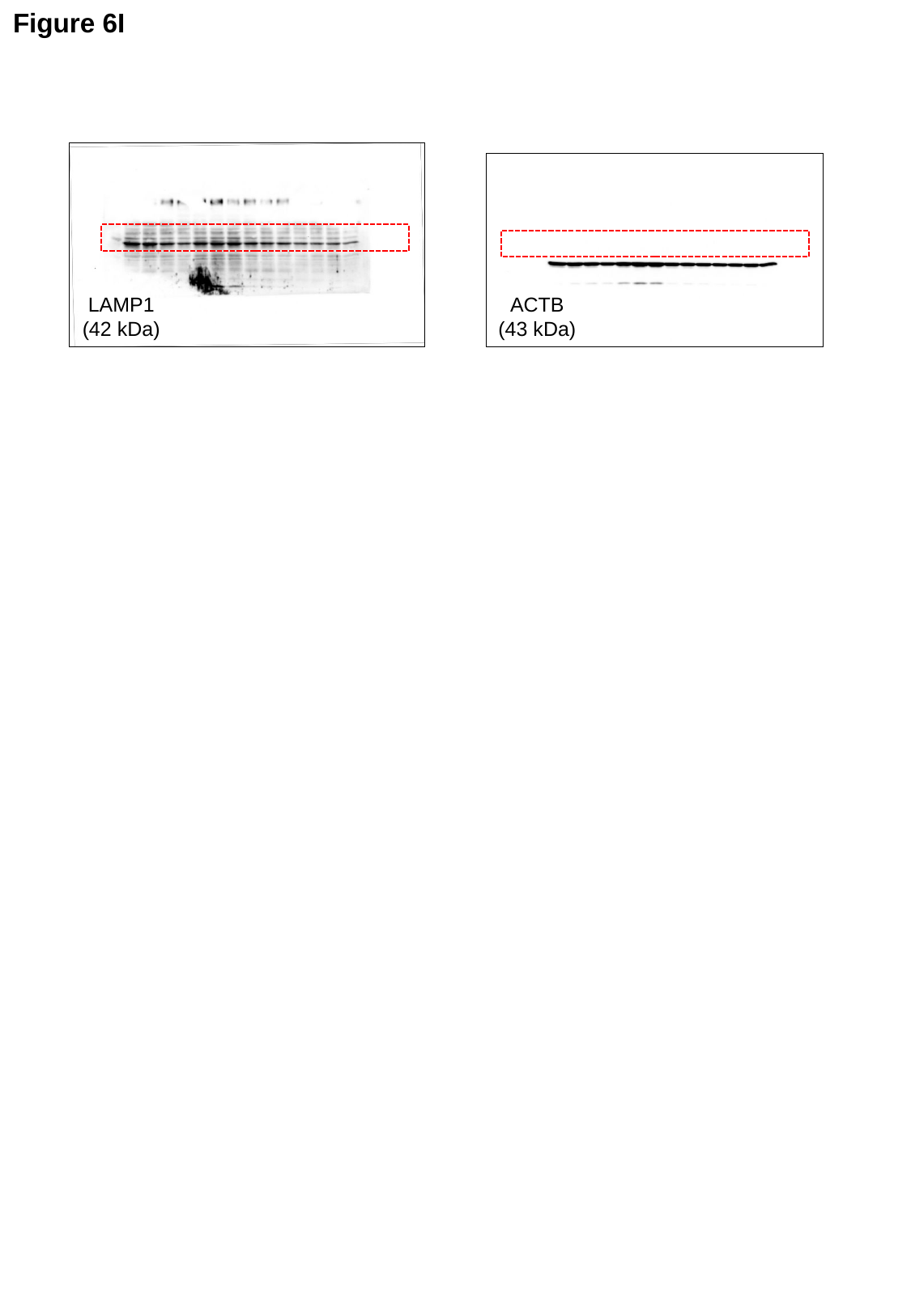

Figure 6I
LAMP1
(42 kDa)
ACTB
(43 kDa)

## Slide 7
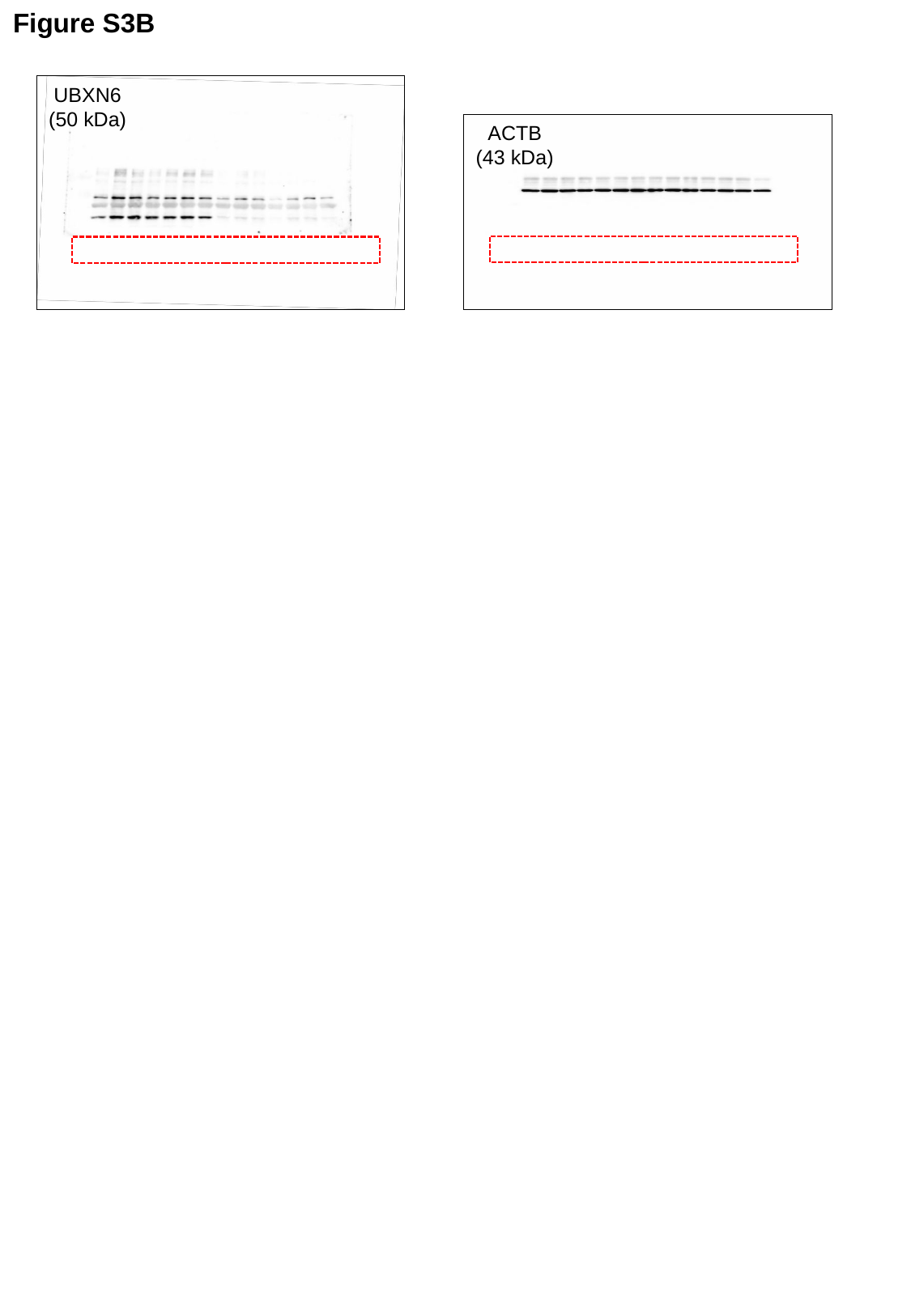

Figure S3B
UBXN6
(50 kDa)
ACTB
(43 kDa)

## Slide 8
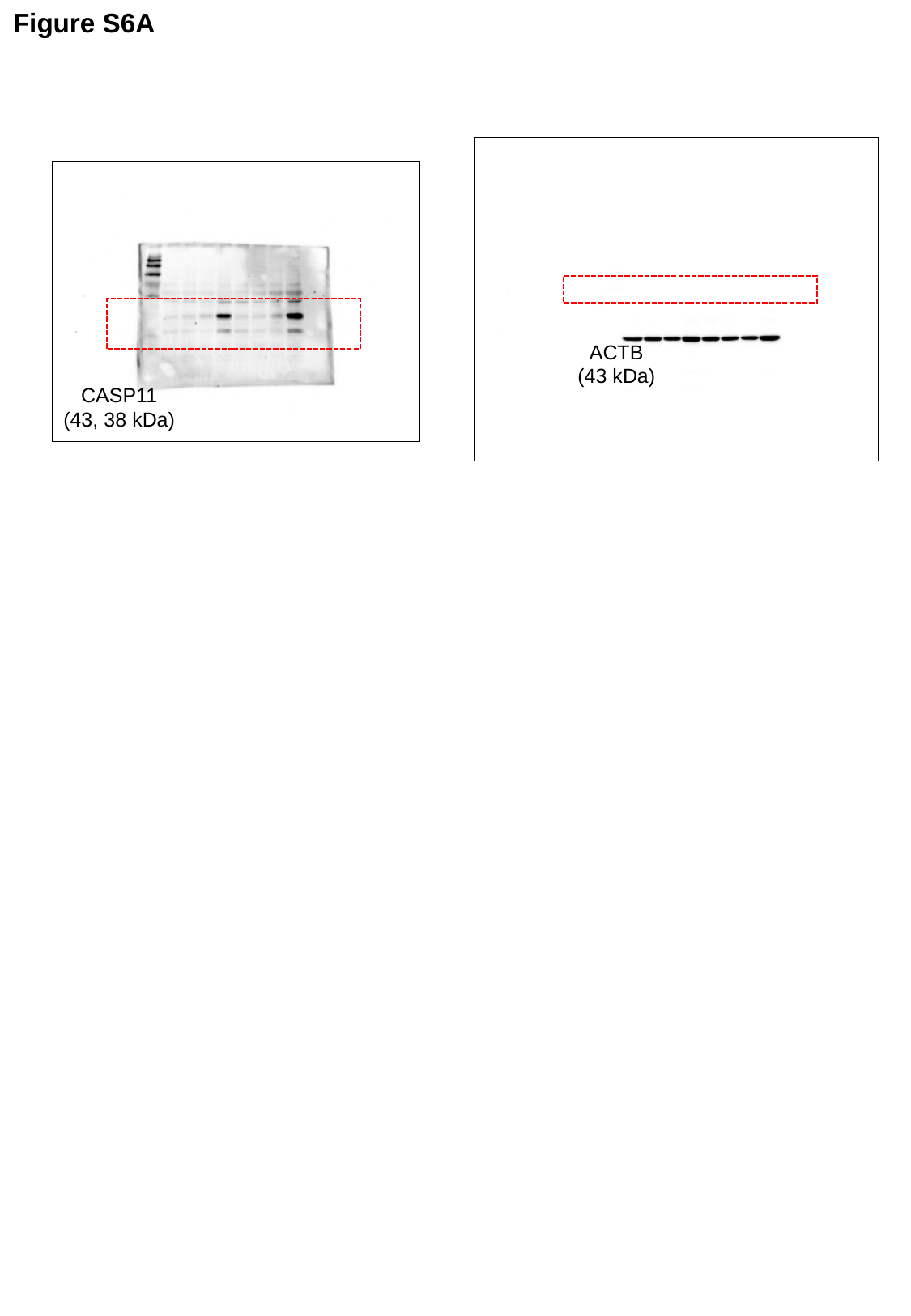

Figure S6A
ACTB
(43 kDa)
CASP11
(43, 38 kDa)

## Slide 9
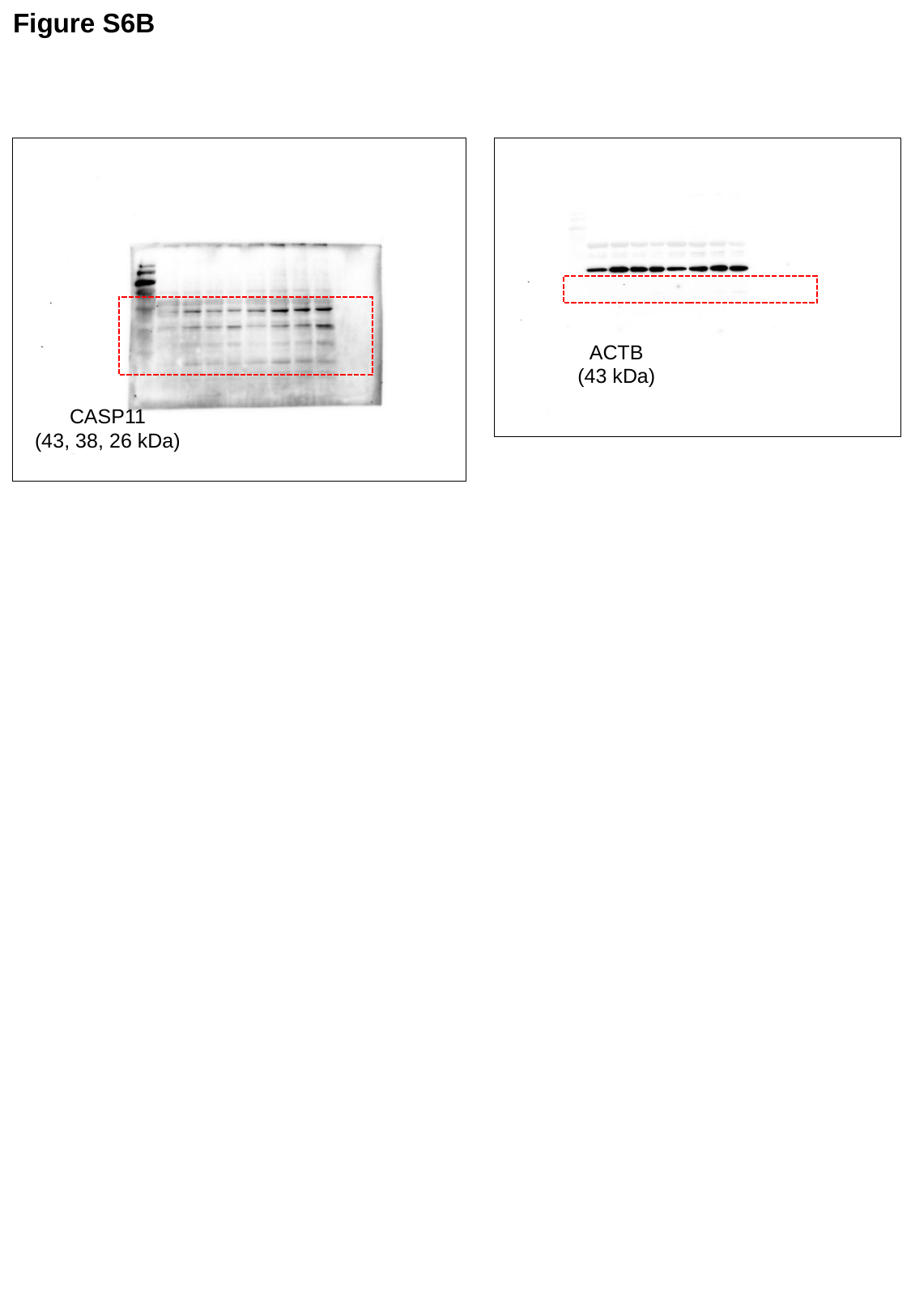

Figure S6B
ACTB
(43 kDa)
CASP11
(43, 38, 26 kDa)
